# Genetic analysis of the “head top shape” quality trait of Chinese cabbage and its association with rosette leaf variation

**DOI:** 10.1038/s41438-021-00541-y

**Published:** 2021-05-01

**Authors:** Xiaoxue Sun, Ying Gao, Yin Lu, Xiaomeng Zhang, Shuangxia Luo, Xing Li, Mengyang Liu, Daling Feng, Aixia Gu, Xueping Chen, Shuxin Xuan, Yanhua Wang, Shuxing Shen, Guusje Bonnema, Jianjun Zhao

**Affiliations:** 1 State Key Laboratory of North China Crop Improvement and Regulation, Key Laboratory of Vegetable Germplasm Innovation and Utilization of Hebei, Collaborative Innovation Center of Vegetable Industry in Hebei, College of Horticulture, Hebei Agricultural University, 071000 Baoding, China; 2 Plant Breeding, Wageningen University and Research, Wageningen, The Netherlands

**Keywords:** Plant morphogenesis, Leaf development

## Abstract

The agricultural and consumer quality of Chinese cabbage is determined by its shape. The shape is defined by the folding of the heading leaves, which defines the head top shape (HTS). The overlapping HTS, in which the heading leaves curve inward and overlap at the top, is the shape preferred by consumers. To understand the genetic regulation of HTS, we generated a large segregating F_2_ population from a cross between pak choi and Chinese cabbage, with phenotypes ranging from nonheading to heading with either outward curving or inward curving overlapping heading leaves. HTS was correlated with plant height, outer/rosette leaf length, and petiole length. A high-density genetic map was constructed. Quantitative trait locus (QTL) analysis resulted in the identification of 22 QTLs for leafy head-related traits, which included five HTS QTLs. Bulked segregant analysis (BSA) was used to confirm HTS QTLs and identify candidate genes based on informative single-nucleotide polymorphisms. Interestingly, the HTS QTLs colocalized with QTLs for plant height, outer/rosette leaf, and petiole length, consistent with the observed phenotypic correlations. Combined QTL analysis and BSA laid a foundation for molecular marker-assisted breeding of Chinese cabbage HTS and directions for further research on the genetic regulation of this trait.

## Introduction

Leafy heads are an important trait of leafy vegetable crops, such as cabbage (*Brassica oleracea*), Chinese cabbage (*Brassica rapa*), and lettuce (*Lactuca sativa*). The leafy head is composed of curving leaves, and these leaves form various head shapes depending on the curvature of the distal end, hereafter referred to as the tops of leaves: leaves with outward curving tops, leaves with inward curving tops that do not overlap, and inward curving leaves with their tops overlapping or forming a spiral^1^. Leaf morphology is determined by leaf cell division and cell elongation rates along the main axes of the leaf^2–4^. The heading leaves curve inward to form a leafy head at the heading stage. The upward curvature of Chinese cabbage heading leaves is facilitated by increased growth of abaxial cells^5^. As a storage organ, the leafy head is an important source of mineral nutrients, crude fiber, and vitamins. Head traits include head weight (HWe), compactness, size, and shape. The Chinese cabbage overlapping head type, in which the heading leaves curve inward and overlap at the top, is the shape that current consumers prefer^6^. However, the molecular mechanism of leafy head formation and regulation of head shape is not fully understood.

The vegetative growth of Chinese cabbage is divided into four stages: seedling, rosette, folding, and heading stages. The leaves in these four stages are different in shape, size, and physiological function^7^. The rosette leaves are large and round with short petioles. These Chinese cabbage rosette leaves provide the basis for plant growth via photosynthesis^8^. A number of recent studies have shown that rosette leaves supply products for leafy head formation and that their size and shape are related to the leafy head shape or size^6,9,10^. In a study by Mao et al.^6^, the correlation between rosette leaves and head shape was demonstrated by using 150 recombinant inbred lines (RILs): round heads with flat rosette leaves, cylindrical heads with rosette leaves with wavy margins, oblong heads with shrinking rosette leaves, and cone-like heads with incurved rosette leaves. In this paper, they also identified that allelic variation in the *TCP4* gene in the *miR319a* recognition sequence modulated head shape by differentially arresting cell division in different leaves. Sun et al.^9^ also showed the correlation between rosette leaf traits, such as leaf length (LL), width, and midvein length, with leafy head traits in a diverse set of 152 Chinese cabbages. Although the phenotypic correlation between rosette leaves and leafy heads has been described, it is unclear how rosette leaves influence leafy head growth and how this is regulated at the molecular level.

Genetic information from quantitative trait loci (QTLs), sequencing‐based bulked segregant analysis (Seq‐BSA), and RNA-sequencing approaches provides clues for understanding Chinese cabbage leaf and leafy head traits. QTLs have been identified for Chinese cabbage heading traits, such as head diameter, head height, and HWe, which are all important components of head yield and quality^9,11–13^. Seq-BSA revealed genomic regions enriched with candidate genes related to plant hormones associated with Chinese cabbage heading type and leafy head formation^14,15^. In addition, several studies have described gene expression profiles during Chinese cabbage development and constructed rosette and inner leaf gene expression networks^16–19^. Although these studies provide insight into the general pathways involved in the formation of leafy heads in Chinese cabbage, they do not explain the genetic control of the relation between rosette leaf growth and leafy head formation.

In this study, we phenotyped an F_2_ population of *B. rapa* derived from a cross between heading Chinese cabbage and nonheading pak choi and analyzed the association between rosette/heading leaves and several heading traits, including the leafy head top shape (HTS). In addition, a combined approach of BSA of pools with contrasting head traits and QTL analysis was used to study rosette leaf growth and the HTS trait at the genetic level. Several colocalizing genomic regions for rosette leaf traits and HTS were identified. Further investigation of these regions resulted in candidate genes for the HTS trait in Chinese cabbage.

## Results

### Phenotype analysis

At the heading stage, the leafy HTS was evaluated for all 1307 F_2_ plants. This resulted in 228 plants in class 4 (inward curving leaves that overlap at the top of the leafy head), 389 plants in class 3 (inward curving leaves that do not overlap at the top of the leafy head), 591 plants in class 2 (with outward curving outer heading leaves), and 99 plants in class 1 (that did not form a leafy head). Based on these four classes of the HTS trait, it was hypothesized that the qualitative trait HTS was controlled by two major genes with recessive epistasis (2MG-RecessiveI) based on SEgregation Analysis (SEA) software analysis (Table S1A). HTS was also scored in intermediate classes, which resulted in the following scores: 1 (37 plants), 1^+^ (60 plants), 2^−^ (63 plants), 2 (521 plants), 2^+^ (7 plants), 3^−^ (47 plants), 3 (233 plants), 3^+^ (109 plants), 4^−^ (28 plants), and 4 (200 plants). The scores of the parental genotypes were PC-101 = “1”, CC-48 = “2”, and F_1_ = “3”, Based on these ten phenotypic classes, it was hypothesized that the qualitative trait HTS is controlled by four major genes with additive and epistatic effects (4MG-AI) based on SEA software analysis (TableS1B). From this population, 104 plants were selected to be phenotyped for additional leaf and leafy head traits. Seventeen plants that did not form a leafy head (N: nonheading) were selected, while from the plants that formed a head, we selected equal numbers of the most obviously different heading types, namely, the OC (the shape of the leaf on the top of the head is outward curving) and O (the shape of the leaf on the top of the head is inward curving with an overlap) HTSs.

**Fig. 1:**
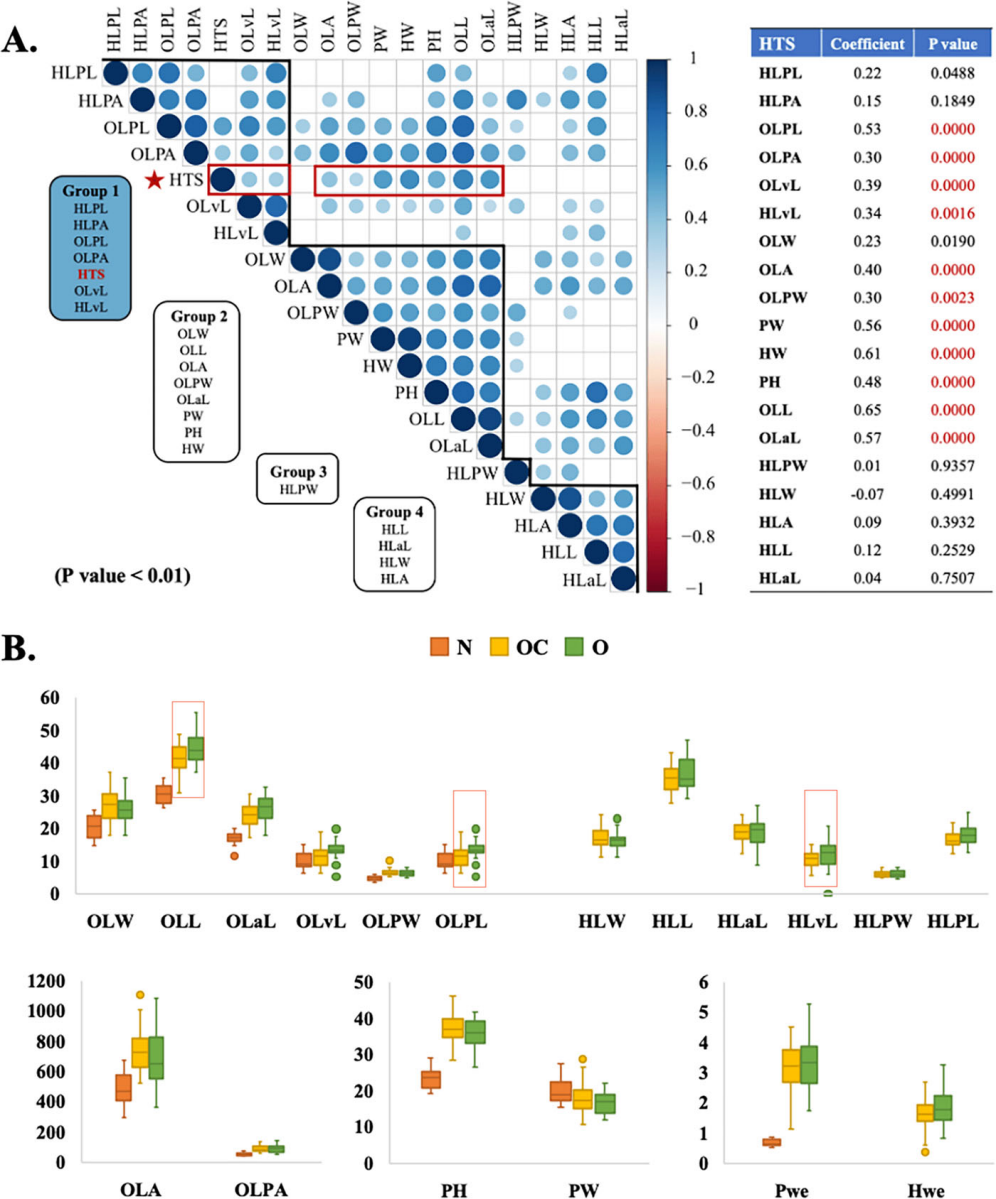
Leaf and leafy head trait analysis of 104 F_2_ plants from the CC-48 × PC-101 cross. **A** Pearson’s correlation analysis for 21 traits in 104 F_2_ plants. The Hclust method was “complete” during analysis. Rectangles around the chart of correlations are based on the results of hierarchical clustering; a *p* value > 0.01 is shown with a blank in the left figure; HTS as the main trait is marked with red. The correlation coefficient and *p* value between HTS and the other traits are shown in the right table. The highest correlation coefficient value is highlighted in red. **B** Box plots of leaf and leafy head traits for nonheading plants (N) and heading plants (OC and O). Traits that are significantly different between O and OC plants are marked with red squares

The HTS and the other 20 traits could be divided into three clusters based on their correlation coefficients (Fig. 1A). Significant phenotypic correlations were found between HTS and all outer/rosette leaf traits (outer leaf width (OLW), outer LL (OLL), outer leaf area length (OLaL), outer leaf midvein length (OLvL), outer leaf area (OLA), outer leaf petiole length (OLPL), outer leaf petiole width, and outer leaf petiole area), plant height (PH), plant width (PW), plant weight (PWe), and HWe at the 0.05 level. Among them, HTS and OLL had the highest correlation coefficient of 0.65. However, HTS was not correlated with most of the head leaf traits (heading leaf width, heading LL, heading leaf area length, heading leaf area, heading leaf petiole width, and heading leaf petiole area), except for head leaf petiole length (HLPL) at the 0.05 level and head leaf midvein length (HLvL) at the 0.01 level.

In nonheading plants (N), the outer/rosette leaf dimensions (O) leaf width (LW), LL, leaf blade length (LaL), leaf midvein length (LvL), LA, LPL, leaf petiole width (LPW), and leaf petiole area (LPA), PH, and PWe values were lower than those of both groups of heading plants (both OC: outward curving heading plants and O: overlapping heading plants) (Fig. 1B). These differences between nonheading plants and heading plants were significant, except for the OLvL. The heading OC and O plants differed only in two outer/rosette leaf traits (OLL and OLPL) and one heading leaf trait (HLvL) and showed no significant difference in other outer or heading leaf traits (Table S2).

### QTL analysis

#### Construction of the linkage map

The F_2_-104 genetic map consisted of ten linkage groups and was constructed using 3194 bin markers (Fig. S1). A recombination bin map and heat map of the recombination fraction were generated to evaluate and verify the quality of the linkage map (Fig. S2). The total map length was 1522.89 cM, with an average intermarker distance of 0.48 cM. The length of each linkage group ranged from 280.91 cM for A03 to 88.49 cM for A02, and the number of markers ranged from 571 markers on A03 to 171 markers on A01 (Table S3). The syntenic map of adjacent markers demonstrated that the distribution of single-nucleotide polymorphism (SNP) markers on the genetic map corresponds well with the reference physical map (Fig. S3).

#### QTL regions and QTL colocalization

QTL analysis of leaf and leafy head traits was performed for the F_2_-104 population using interval mapping (IM) and composite IM (CIM) methods (Table 1).

**Table 1:** Quantitative trait loci found in the F_2_ population using IM and CIM methods

LOD threshold	IM QTL	LG	Position (cM)	Adjacent markers	LOD	Exp (%)	CIM QTL	Position (cM)	Adjacent markers	LOD	Exp (%)
HTS 3.5447	HTSQ1A4	A04	155.0	A04-15478117/A04-15979628	4.833	6.1	CIM HTSQA4	148.5	A04-15478117/A04-15979628	5.923	16.8
	HTSQ2A4	A04	165.8	A04-16048222/A04-17510914	4.570	1.7					
	HTSQ3A4	A04	177.9	A04-16269682/A04-18344243	4.335	0.5					
	HTSQ4A4	A04	192.8	A04-17632945/A04-19248106	4.115	2.4					
	HTSQ5A5	A05	142.0	A05-21990254/A05-22738372	3.660	2.5	CIM HTSQA5	176.2	A05-23285233/A05-24488014	4.795	17.6
	HTSQ6A5	A05	171.3	A05-23186326/A05-24488014	4.888	1.1					
	HTSQ7A5	A05	177.7	A05-23285233/A05-24488014	4.895	4.0					
OLL 3.4663	OLLQ1A5	A05	130.3	A05-20924718/A05-22407474	3.699	0.1	CIM OLLQA5	143.0	A05-22314735/A05-22421784	6.237	21.6
	OLLQ2A5	A05	144.0	A05-21990254/A05-22953127	5.500	4.6					
	OLLQ3A5	A05	152.0	A05-22330408/A05-23285134	4.479	1.3					
OLPL 3.4556	OLPLQ1A5	A05	144.2	A05-21990254/A05-22953127	5.389	6.6	CIM OLPLQA5	144.12	A05-22314735/A05-22659381	4.679	21.5
	OLPLQ2A5	A05	153.6	A05-22330408/A05-23285233	3.977	1.5					
HLPL 3.6145	HLPLQ1A9	A09	37.4	A09-5581456/A09-8022134	3.787	0.1	—	—	—	—	—
	HLPLQ2A9	A09	46.1	A09-6343345/A09-8022357	4.254	0.7					
	HLPLQ3A9	A09	58.0	A09-8015506/A09-8679816	4.056	0.5					
HLvL 3.5543	HLvLQ1A6	A06	32.8	A06-4629015/A06-7988042	4.27	2.8	—	—	—	—	—
	HLvLQ2A6	A06	60.0	A06-8698682/A06-16848734	3.87	0.9					
PH 3.4356	PHQ1A4	A04	143.8	A04-14635707/A04-15484529	4.622	4.0	CIM PHQA4	148.4	A04-17550649/A04-17730212	5.923	4.8
	PHQ2A4	A04	156.0	A04-15478117/A04-16269668	3.906	1.3					
	PHQ3A4	A04	168.0	A04-15735957/A04-17038551	3.879	0.9					
	PHQ4A4	A04	182.3	A04-17510914/A04-17730212	4.446	3.0					
HWe 3.6155	HWeQ1A8	A08	21.2	A08-1846413/A08-4735716	3.969	16.3	—	—	—	—	—

By the IM analysis method, four QTLs (HTSQ1, 2, 3, and 4) for HTS were detected on A04, and three QTLs (HTSQ5, 6, and 7) were detected on A05. HTSQ1A4 and HTSQ7A5 had the highest logarithm of the odds (LOD) scores (4.833 and 4.895) and explained ~6.1% and 4% of the variation, respectively. For leaf-related traits, three QTLs for OLL (OLLQ1, 2, and 3) and two QTLs for OLPL (OLPLQ1 and 2) were detected on A05. Three QTLs for HLPL (HLPLQ1, 2, and 3) were detected on A09, and two QTLs for head leaf vein length (HLvLQ1 and 2) were detected on A06. For leafy head traits, a total of four QTLs (PHQ1, 2, 3, and 4) for the PH traits were detected on A04, with LOD values ranging from 3.879 to 4.622, and one QTL for the HWe (HWeQ1A8) trait was detected on A08. By the CIM analysis method, two QTLs for HTS, namely, CIM_HTSQA4 and CIM_HTSQA5, were detected on A04 and A05, which explained ~16.9% and 17.6%, respectively. Together, these findings accounted for 34.5% of all the phenotypic variation in the HTS trait. For the OLL, OLPL, and PH traits, single QTLs were detected on A05 (CIM_OLLQA5 and CIM_OLPLQA5) and A04 (CIM_PHQA4). The genes in these QTL regions are shown in Table S4.

**Fig. 2:**
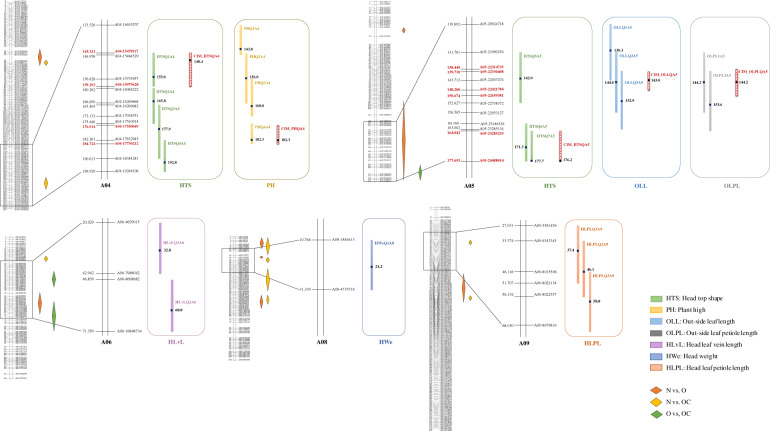
Quantitative trait locus hotspots for the F_2_-104 population from the CC-48 × PC-101 cross. Zoomed-in depiction of QTL hotspots of HTS (green), PH (yellow), OLL (light blue), OLPL (gray), HLvL (purple), HWe (dark blue), and HLPL (orange) on A04, A05, A06, A08, and A09 by the IM analysis method. QTL hotspots determined by the CIM analysis method are highlighted by the red color bar. SNPs with different (*p* < 0.05) allelic polymorphisms between different phenotype bulk enrichment regions in linkage groups are marked by color diamonds

For all mapped QTLs of the Chinese cabbage leaf and leafy head traits, overlapping genomic regions were identified between HTS and PH and the outer/rosette leaf traits OLL and OLPL (Fig. 2). HTS trait QTLs were located on both A04 and A05. The head-type shape traits HTSQ1, HTSQ2, HTSQ3, and HTSQ4 and PH traits PHQ1, PHQ2, PHQ3, and PHQ4 were located at the bottom part of A04 (145.321–184.724 cM). The bottom part of A05 (131.781–164.042 cM) showed overlapping QTLs for HTS, OLL, and OLPL: HTSQ5, HTSQ7, OLLQ1, OLLQ2, OLLQ3, OLPLQ1, and OLPLQ2.

### Candidate genes selected by BSA

We generated three independent plant DNA pools, each representing different head-type shapes. In the bulks, the plants were all barcoded, and SNP markers with different allele frequencies between bulks with different phenotypes were selected (*p* < 0.05).

**Fig. 3:**
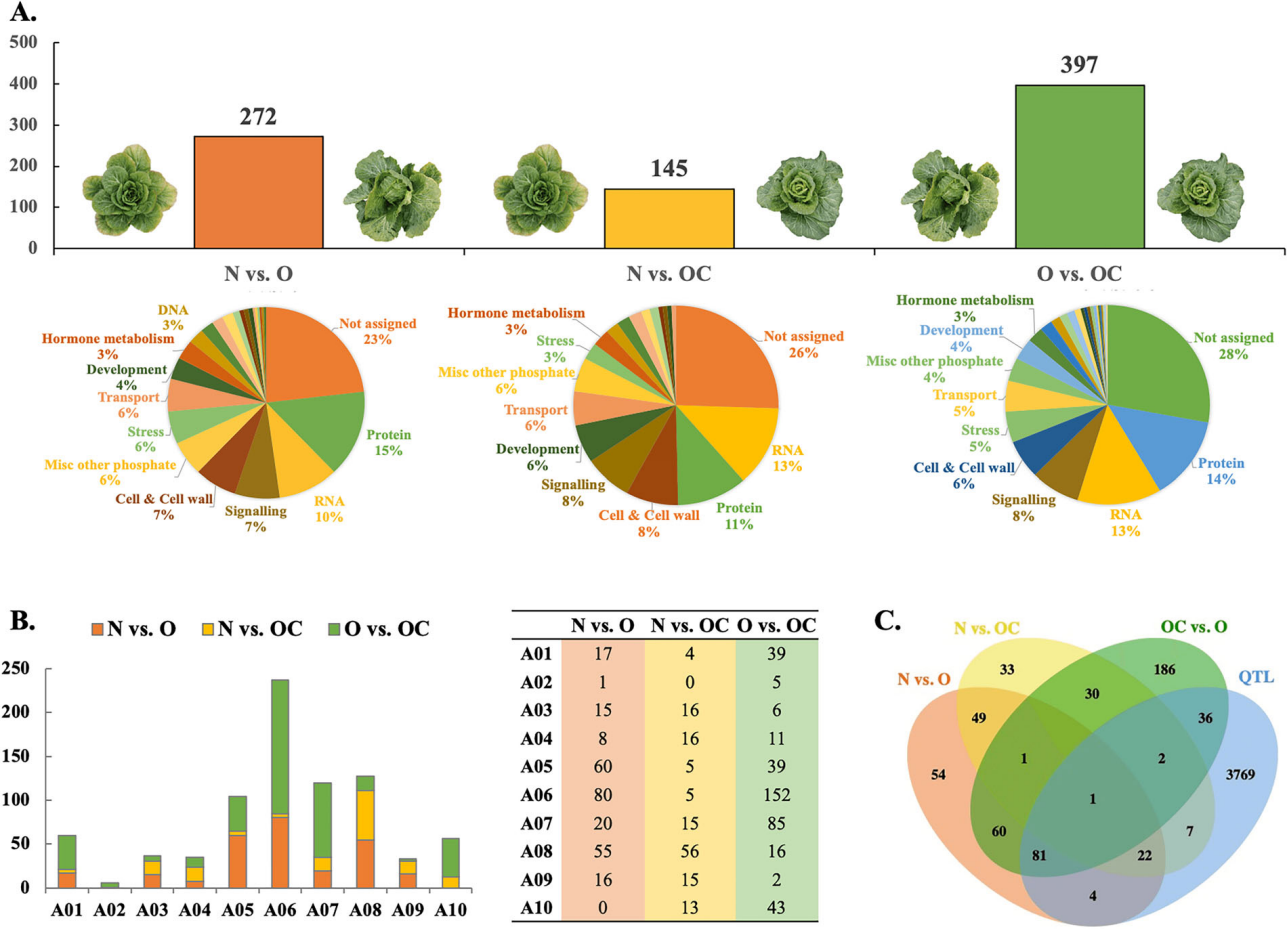
Genes with significantly different SNP allele frequencies between different pools (N, OC, and O). **A** The number of candidate genes and the percentage/number of genes that belong to MapMan functional categories in the three group comparisons (“N vs. O,” “N vs. OC,” and “O vs. OC”). **B** The linkage group distribution of candidate genes with significantly different SNP allele frequencies between different pools (N, OC, and O). **C** A four-way comparison of the total number of genes detected in the three BSA group comparisons and QTL regions for HTS, OLL, OLPL, HLvL, PH, and HWe

We focused on SNPs that had significantly different allele frequencies among pools in upstream, downstream, intergenic, intron, and exon regions of genes. This included 272 genes in the “N vs. O” pool comparison, 145 genes in the “N vs. OC” comparison, and 398 genes in the “O vs. OC” comparison (Fig. 3A and Table S5). In the “N vs. O” comparison, most candidate genes were mapped to regions on A05 (60 genes), A06 (80 genes), and A08 (55 genes); in the “N vs. OC” comparison, the genes were distributed on A08 (56 genes); and in the “O vs. OC” comparison, they were distributed on A06 (152 genes) and A08 (85 genes) (Fig. 3B). We investigated the MapMan functional categories to which these genes belonged (Fig. 3A). In addition to the common top three categories (not assigned, protein, and RNA), the cell and cell wall and signaling categories were highly represented in these three group comparisons. In the cell and cell wall functional category, the majority of the genes were involved in the cell organization subcategory. In the signaling category, the majority of the genes were involved in the receptor kinase subcategory. In total, 566 candidate genes were included in these three pooled comparisons (Table S5). Only two genes, namely, *Bra001163* (embryo defective 2016: EMB2016) in the development category and *Bra016948* (glutamate synthase 2: GLU2) in the N-metabolism category, were identified in all three comparisons (Fig. 3C).

As expected, some of the candidate genes with significant allele frequency differences in the “N vs. O,” “N vs. OC,” and “O vs. OC” comparisons colocalized with QTL regions on A04, A05, A06, A08, and A09 (Fig. 2). For the HTS trait, 15 candidate genes detected by BSA were located in the CIM_QTL regions on A04 and A05. According to the annotation information, five genes (*Bra017274*, *Bra029597*, *Bra029713*, *Bra039431*, and *Bra039444*) were in the protein category, four genes (*Bra039420*, *Bra039421*, *Bra039462*, and *Bra039446*) were in the cell and cell wall category, and two genes (*Bra020751* and *Bra039445*) were in the RNA category. Among them, *Bra017274* (*F-box family protein*; A04:15735732..15736841), *Bra029597* (*RING/FYVE/PHD zinc finger superfamily protein*; A05:23809459..23812937), and *Bra039421* (*PEM: plant invertase/pectin methylesterase inhibitor superfamily*; A05:24250991..24253260) included SNPs causing amino acid changes (Table S5). Seventy-three common genes were identified in the “N vs. O” and “N vs. OC” comparisons (Fig. 3C), and 59% (43) of the genes were mapped to A08. Among these 43 genes, 16 colocalized with the HWeQ1A8 QTL. The cell organization gene *Bra014031* (A08:4344956..4347618) and cell wall degradation gene *Bra014180* (A08:2743942..2745200) with SNPs causing amino acid changes were among these 16 genes in HWeQ1A8.

## Discussion

The leafy head of Chinese cabbage is an important agronomic trait associated with both yield and quality. Compared to heading Chinese cabbage, nonheading plants (such as pak choi) are generally smaller and produce less biomass (lower PWe or total leafy yield). For heading Chinese cabbage, the head shapes include round, oblong, cylindrical, and cone-like head shapes^6^. HTS refers to the shape of the top of the leafy head, which is affected by the shape, overlap, and curvature of the outer heading leaves. Currently, with the improvement in people’s living standards, Chinese consumers, especially from Northern China, prefer leafy heads of the overlap head-type shape. For this reason, it is an important agronomic trait for breeders to screen, and it is interesting for researchers to study because it involves variation in leaf curvature. Recently, several papers have been published about QTL mapping of the Chinese cabbage leafy head. Most of these studies analyzed leafy head formation (head or nonhead), the heading degree (nonheading, intermediate heading or heading), and the heading shape index (head width diameter/length diameter)^10,11,15^. Only one paper investigated HTS, based on a cross between two heading Chinese cabbages^14^. Due to the different parental genotypes used in their mapping study, a different candidate region on A06 was detected. This study provides new insights into the HTS phenotype by combining phenotypic observations and genetic analyses using a large F_2_ progeny from a cross between Chinese cabbage and pak choi.

### Head-type shape is associated with the length of both the leaf blade and petiole/midvein of the outer rosette leaves

HTS was judged based on the heading leaf top shape at the heading stage. The F_2_ population was segregated into various HTS types, and we tried to fit a genetic model to the segregation data of the leafy head shape based on the HTS phenotypes of the 1307 F_2_ plants. However, regardless of whether we scored the HTS into four or ten classes, we could not clearly select the most likely genetic model. This was likely caused by the fact that the scored phenotype is a combination of traits that are all under the control of different genetic loci^10,20^. To reveal genetic regulation, we should identify and score the subtraits that make up the final head shape-type trait. These subtraits include the curvature of the petiole, angle of the midvein to the soil, head size and weight, LL, and curvature of the distal end of the leaf blade.

We phenotyped many leaf traits, for both the outer rosette leaves and the outer leafy head leaves, at the heading stage. Interestingly, we showed that the traits related to the shape and size of the outer rosette leaves were correlated with HTS. Surprisingly, these same traits of the heading leaf itself were not correlated with HTS. In addition, when we compared the leaf traits of the overlapping “O” HTS group with those of the outward curving “OC” HTS group, significant differences were detected between their outer/rosette leaf and petiole traits (LL and LPL), while the shapes of their heading leaves did not differ. Normally, in *Arabidopsis*, outer (old) rosette leaf senescence proceeds during plant growth to recycle nutrients that become available for the actively growing parts^21,22^. However, in Chinese cabbage, rosette leaves remain active until the leafy heads mature, likely performing photosynthesis in the fast-growing leafy head^18^. Both this study and previous studies showed a correlation between the size and shape of rosette leaves and leafy head shape^6,9^. HTS is clearly associated with outer rosette LL and leaf petiole length (LPL) (this study). These results indicate that the outer leaves not only provide the products for leafy head growth but also affect the shape of the head.

At the QTL level, we detected QTLs for rosette leaf-, outer heading leaf-, and leafy head-related traits in this study. We did not detect the same QTLs for rosette LL and width or rosette LPL compared to the previous QTL study^9^. In addition to environmental variation, in these studies, the timing of phenotypic data collection differed: we measured the rosette leaf traits at the heading stage in this study, but phenotyped the growing rosette leaves at rosette stages (30, 34, 37, 41, 44, and 48 days after sowing (DAS)) in a previous QTL study. In a study by Sun et al.^9^, QTLs for rosette LPL also varied over time (DAS), and generally, LOD scores were lower when measured at later stages. The phenotyping of the leafy heads was performed at the same developmental stage in both studies, the heading stage. In both studies, QTLs for the leafy head trait “weight” (HWe) were detected at the top of A08 (Fig. S4). Another QTL study using 150 RILs derived from a cross between heading and nonheading Chinese cabbage also identified QTLs for HWe in linkage group A08: 35.2–62.3 cM in 2010 and 27–34.5 cM in 2012^10^. In the smaller populations of the previous study, the HTS trait was not scored^9^. In the present study, the F_2_ population was larger, which allowed us to study a subpopulation with balanced representation of the extreme phenotypes in more detail. In addition, a high-density genetic map was constructed for this subpopulation, and QTL analysis was combined with a BSA approach. This made it possible to highlight candidate genes with SNPs in the HWeQ1A8 QTL region.

The colocalized QTLs indicate the causal relationships among the traits^23^. Colocalized QTLs for pod-related traits (pod length, pod width, and hundred-pod weight) have been identified on A05 and contribute to phenotypic variation for these pod-related traits^24^. In rice, colocalization between PH- and flowering time-related QTLs was identified, and the major gene *Ghd7*, which has pleiotropic effects on PH and heading date, was identified^23,25^. Here, we detected two QTLs for the HTS trait. Colocalization of HTS with outer/rosette LL and rosette LPL (OLL/OLPL) was identified on A05, and that of HTS with PH was identified on A04. These results were consistent with the phenotypic results: there were strong correlations among outer/rosette LL, LPL, and HTS traits, and significant differences existed in the OLPL, OLvL, and HLvL traits between “O” and “OC” HTS plants. This phenomenon was not unexpected and indicated pleiotropic effects of single genes or tight linkage. We identified an HTS QTL on A05. Interestingly, in an independent study on an F_2_ population derived from a cross between two Chinese cabbages with different HTSs, BSA of the “O” and “OC” types identified significant SNPs on A05 (21700337..24709181) overlapping with the QTL region for HTS identified in this study (Fig. S5; unpublished data). The colocalized QTLs provide a good foundation for fine mapping and for researching HTS and related traits at the molecular level.

### Molecular pathways and candidate genes for the HTS trait

HTS is a complex trait that is controlled by endogenous and environmental factors. In this study, we constructed three pools, namely, one nonheading plant pool and two pools of plants with different HTSs, for BSA to identify genomic regions containing genetic loci affecting these traits. In this study, the number of selected candidate genes was higher in the “O vs. OC” comparison than in the “N vs. O/OC” comparison, and most of the candidate genes were colocalized with QTLs for head shape and leaf traits. The expression patterns of these candidate genes were checked in three different genotypes, namely, a nonheading pak choi and two heading Chinese cabbages (one with “OC” HTS and the other with “O” HTS), at the seedling, rosette, and heading stages (Table S7). All candidate genes were expressed in leaves; however, only a few candidate genes were differentially expressed between these genotypes.

Leaf growth is strongly associated with leafy head formation in Chinese cabbage and includes leaf primordium initiation, leaf polarity formation, cell division, cell expansion, and cell differentiation phases^26^. During these phases, the arrangement of cells is indicative of changes in leaf shape^2,4^. Our BSA results showed many candidate genes in the cell wall and cell cycle category. In particular, *Bra039421* was mapped to the HTS QTL_CIM on A05. The SNP (A/C) located in exon 1 (540 bp) of *Bra039421* resulted in an amino acid change from “Gln” to “His.” According to classic cell theory, changes in cellular behavior are responsible for mutant morphology^27^. Thus, the genes in these QTLs may have functions that play roles in HTS formation in Chinese cabbage. Interestingly, genes common between the “N vs. OC” and “N vs. O” comparisons were mainly localized on A08 in the HWe QTL. Heading Chinese cabbage plants were consistently heavier than nonheading plants. We identified several candidate genes for this heading/nonheading comparison (N vs. OC/O), which were related to total PWe. *Bra014180* in the cell wall degradation functional category encodes USPL1 (*AT1G49320* in *Arabidopsis*) targeted to protein storage vacuoles, which is particularly interesting because it is closely related to seed development, protein storage vacuoles, and lipid vesicle morphology and behaves like a storage protein^28^. In addition, *Bra014180* with an SNP (A/G) on exon 2 (444 bp) caused an amino acid change (Ile/Met) and displayed higher transcript abundance in pak choi than in two Chinese cabbage varieties at early heading stages, with fold change >1.5 and adjusted *p* value ≤ 0.01.

In conclusion, we phenotyped both leaf and leafy head traits of an F_2_ population from a cross between a nonheading pak choi and a heading Chinese cabbage and identified correlations between HTS and other rosette leaf traits. These correlations between rosette leaf traits and head-type shape were confirmed by the colocalized QTLs for these traits. This finding is a good foundation for further research on the genetic regulation of the HTS trait in Chinese cabbage and provides tools for molecular marker-assisted selection of leafy head shape in Chinese cabbage. In addition, by combining QTL and BSA results, we identified a few genes with interesting annotations as candidate genes for quantitative trait loci for leafy head formation. These results will help us understand the genetic control of head shape traits in cabbage.

## Materials and methods

### Plant materials

**Fig. 4:**
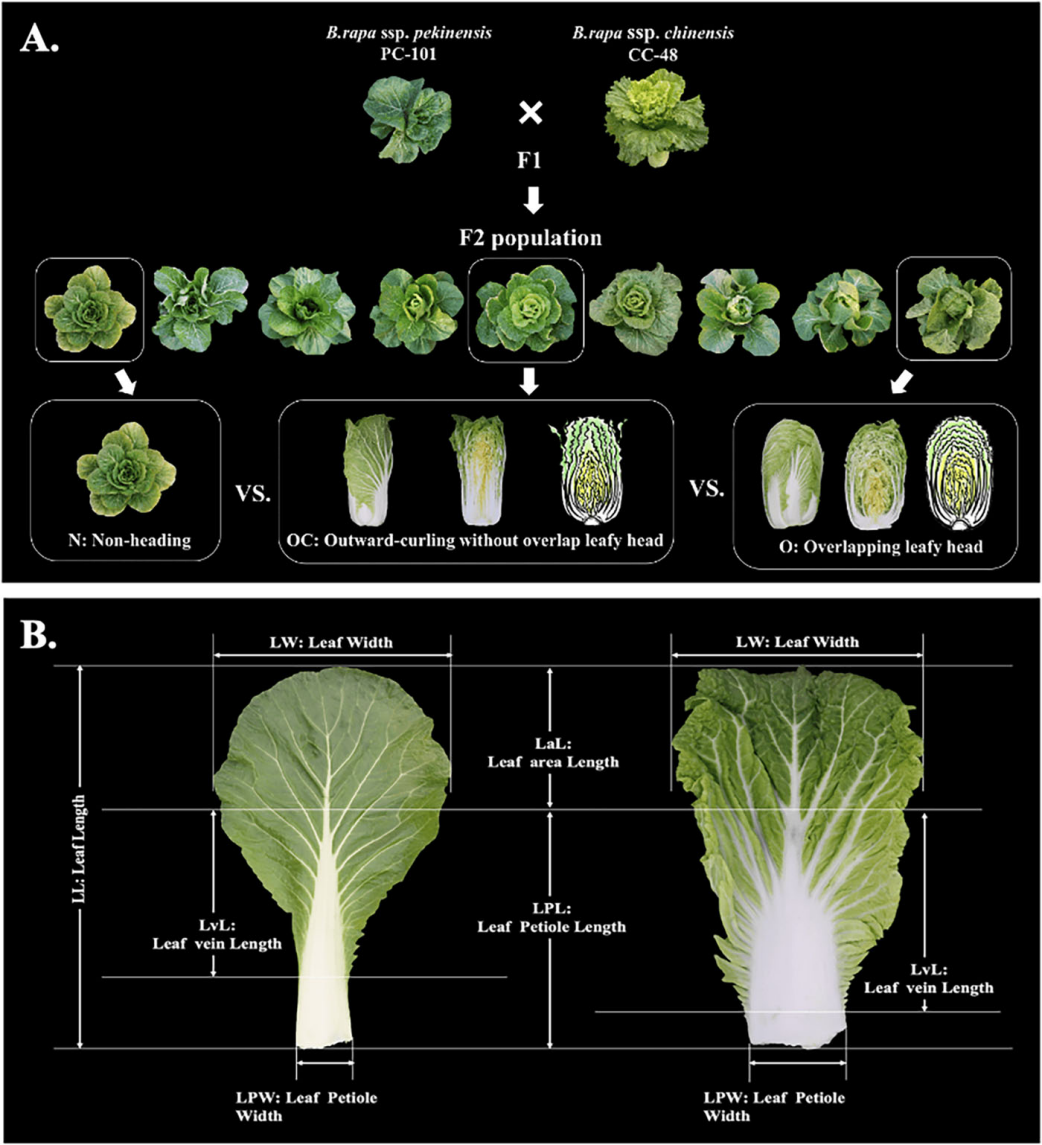
Construction and phenotype of the Chiense cabbage F_2_ population. **A** The strategy for constructing the Chinese cabbage F_2_ population and the phenotype variation of the leafy head top shape (HTS) trait. N nonheading type, OC leafy head with outward curving top leaf without overlap, and O leafy head with overlapping top leaf. **B** Description of the measurements of leaf traits

An F_2_ population was derived from a cross between a heading Chinese cabbage (CC-48: CGN06867, origin Soviet Union) and a nonheading pak choi (PC-101: CGN13926, origin China) (Fig. 4A). The F_2_ population (*n* = 1307) plus their parental lines were sown in seeding soil in a greenhouse in September 2017, and 2 weeks later, the plants were transplanted to an open field at Hebei Agriculture University (Baoding, Hebei, China) and grown under short-day conditions until December 2017.

### Phenotyping and phenotypic data analysis

HTS, as the main trait to be studied in this research, was classified on a 1–4 scale (1. nonheading shape; 2. the shape of the leaf on the top of the head is outward curving; 3. the shape of the leaf on the top of the head is inward curving without overlaps at the top, which we identified as the intermediate phenotype between class 2 and 4; 4. the shape of the leaf on the top of the head is inward curving and overlaps at the top) by visual observation at the heading stage for the F_2_ plants (*n* = 1307) (Fig. 4A).

In addition, another 20 leaf and leafy head traits were evaluated at the same time for 104 F_2_ plants that represented the different HTS classes (Fig. 4B and Table 2). For leaf traits, the LL, LaL, LvL, LW, leaf area, LPL (here, we measured the sum of leaf petiole and white midvein length), LPW, and LPA were measured for the largest outer leaf (O) and for the largest and most outward curving heading leaf (H). For plant traits, PH and PW were evaluated. For leafy head traits, we determined the total PWe (including outer and heading leaves) and HWe (after discarding the loose outer leaves).

**Table 2:** Schematic diagram and description of the evaluated phenotypic traits

Traits	Abbreviation	Description
Outer leaf length	OLL	Length of the largest outer leaf (cm)
Outer leaf area length	OLaL	Length of the largest outer leaf area (cm)
Outer leaf midvein length	OLvL	Length of the largest outer leaf midvein (cm)
Outer leaf width	OLW	Width of the largest outer leaf (cm)
Outer leaf area	OLA	Area of the largest outer leaf (cm^2^)
Outer leaf petiole length	OLPL	Length of the largest outer leaf petiole (cm)
Outer leaf petiole width	OLPW	Width of the largest outer leaf petiole (cm)
Outer leaf petiole area	OLPA	Area of the largest outer leaf petiole (cm^2^)
Heading leaf length	HLL	Length of the largest curling heading leaf (cm)
Heading leaf area length	HLaL	Length of the largest curling heading leaf area (cm)
Heading leaf midvein length	HLvL	Length of the largest curling heading leaf midvein (cm)
Heading leaf width	HLW	Width of the largest curling heading leaf (cm)
Heading leaf area	HLA	Area of the largest curling heading leaf (cm^2^)
Heading leaf petiole length	HLPL	Length of the largest curling heading leaf petiole (cm)
Heading leaf petiole width	HLPW	Width of the largest curling heading leaf petiole (cm)
Heading leaf petiole area	HLPA	Area of the largest curling heading leaf petiole (cm^2^)
Plant height	PH	Length of the plant with external outer leaves (cm)
Plant width	PW	Width of the plant with external outer leaves (cm)
Plant weight	PWe	Length of the leafy head without external leaves (cm)
Head weight	HWe	Weight of the leafy head without external leaves (g)
Head top shape	HTS	Overlap degree of the top of heading leaf

The inheritance model analysis for a plant quantitative trait (HTS) was analyzed by SEA software^29^. The phenotypic data were analyzed by Statistical Package for the Social Sciences (SPSS, IBM) software, including the mean, minimum, maximum, standard deviation, variance, and *t* test, using *p* < 0.05. The correlation coefficients between traits were analyzed using an R package (“corrplot” package: http://www.R-project.org) with the Pearson’s correlation coefficient.

### Genotyping and SNP discovery

Genomic DNA from individual plants was extracted from young leaves of parents and their F_2_ progeny plants (*n* = 104) by the CTAB method. The genotyping-by-sequencing (GBS) method was used to generate ApeKI-associated DNA fragments for sequencing on the Illumina HiSeq2000 platform at the Gene Denovo Company, China, and SNP genotyping and evaluation were then performed^30^. The “Chiifu” genome V1.5 was used as the reference sequence for the alignment of sequenced reads by the BWA software^31^. A total of 258,443 genetic variations (234,421 SNPs and 24,022 indels), with indels representing 9% of the total, were identified, and 71.8% of these SNPs resided in intergenic regions. Among the intergenic SNPs discovered, 11.3% were within 5 kb upstream, and 10.5% were within 5 kb downstream, of an open-reading frame. In addition, 14.9% of the SNPs were observed in exonic regions and 13.2% in introns. For the SNPs located in the coding region (exon), 56.9% were synonymous (silent) mutations, and 40.4% resulted in a change in amino acids (nonsynonymous substitutions) or stop codons. For the SNPs located in introns, 162 SNPs were observed at intron splicing sites that potentially alter the function of these genes.

The 234,421 SNPs were filtered for those that showed polymorphisms between parent lines (CC-48 and PC-101) and had allele frequencies >60% in the F_2_ population. After filtering, a set of 16,570 SNPs were discovered through GBS, of which 8998 were “aa × bb”, 1165 were “hk × hk”, 2612 were “lm × ll”, and 3795 were “nn × np” segregation types^32^. As the F_2_ population was derived from a cross between homozygous parents (DH lines), 8898 “aa × bb” parental markers that formed 5973 bins were used as input data for “JoinMap” to construct the genetic linkage map. After creating population nodes, “ML mapping” was used to assign the markers into linkage groups, and “Kosambi’s” mapping function was used to construct genetic maps.

### Identification of QTLs and BSA to identify candidate genes for leafy head traits

#### Genetic map construction and QTL mapping

A total of 8998 polymorphic homozygous SNPs in both parents (aa × bb) were used to generate a genetic map of the F_2_ population. SNPs in the F_2_ population were only used if the parental source of the alleles could be unambiguously assigned. The F_2_ plants (*n* = 104) were genotyped for a set of 8998 markers covering the ten linkage groups. A subset of 3194 specific SNP markers was used to construct the F_2_ genetic linkage map in this study. Genetic mapping was performed using an R package^33,34^. Based on the genetic map, QTL regions for the phenotyped traits were identified by IM analysis with the R/qtl package^35^. The LOD critical values (Table S6) for accepting the presence of potential QTLs were determined by permutation analyses (*p* < 0.05)^36^.

#### Sequencing‐based bulked segregant analysis

In addition, a BSA approach was used to identify genomic regions linked to the leaf blade top curvature trait. Four leafy head phenotypes were distinguished: no leafy head, with leaves that do not form a leafy head and are fully curved outward (N: 17 plants). Three groups of 30 plants with different heading types were selected (outward curve (OC): 30; intermediate phenotype: 30; and overlapping (O): 30). For BSA, we divided 77 F_2_ plants into three bulks (N: 17 nonheading plants, with the two most different heading phenotypes; OC: 30 outward curving heading plants; O: 30 overlapping heading plants) corresponding to the different leafy head shapes. These three bulks were genotyped with the 8998 selected SNP markers. Markers showing allelic polymorphisms between different phenotype bulks for leafy head shape were selected based on allele frequency. We selected subsets of polymorphic SNPs by comparing different bulks (N vs. O, N vs. OC, and O vs. OC).

## Supplementary information

Figure S5

Table S1

Table S2

Table S3

Table S4

Table S5

Table S6

Table S7

Figure S1

Figure S2

Figure S3

Figure S4
