# Supplementary figures and images for: Genetic analysis of the “head top shape” quality trait of Chinese cabbage and its association with rosette leaf variation
Source: Hortic Res. 2021 May 1;8:106. doi: 10.1038/s41438-021-00541-y (PMC8087666; doi:10.1038/s41438-021-00541-y)

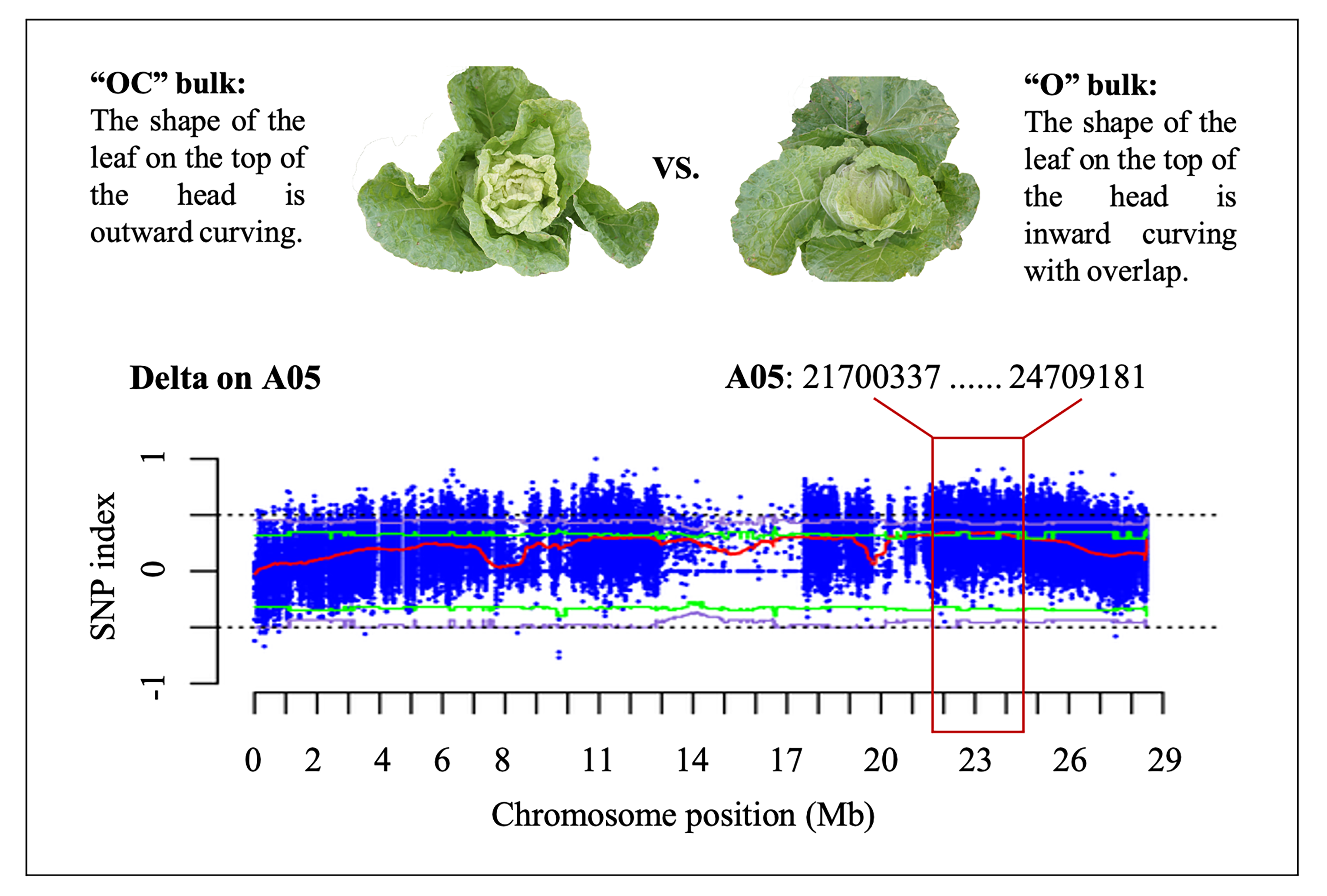

Supplement: Supplementary file 1 — Figure S5 [file 41438_2021_541_MOESM1_ESM.png]

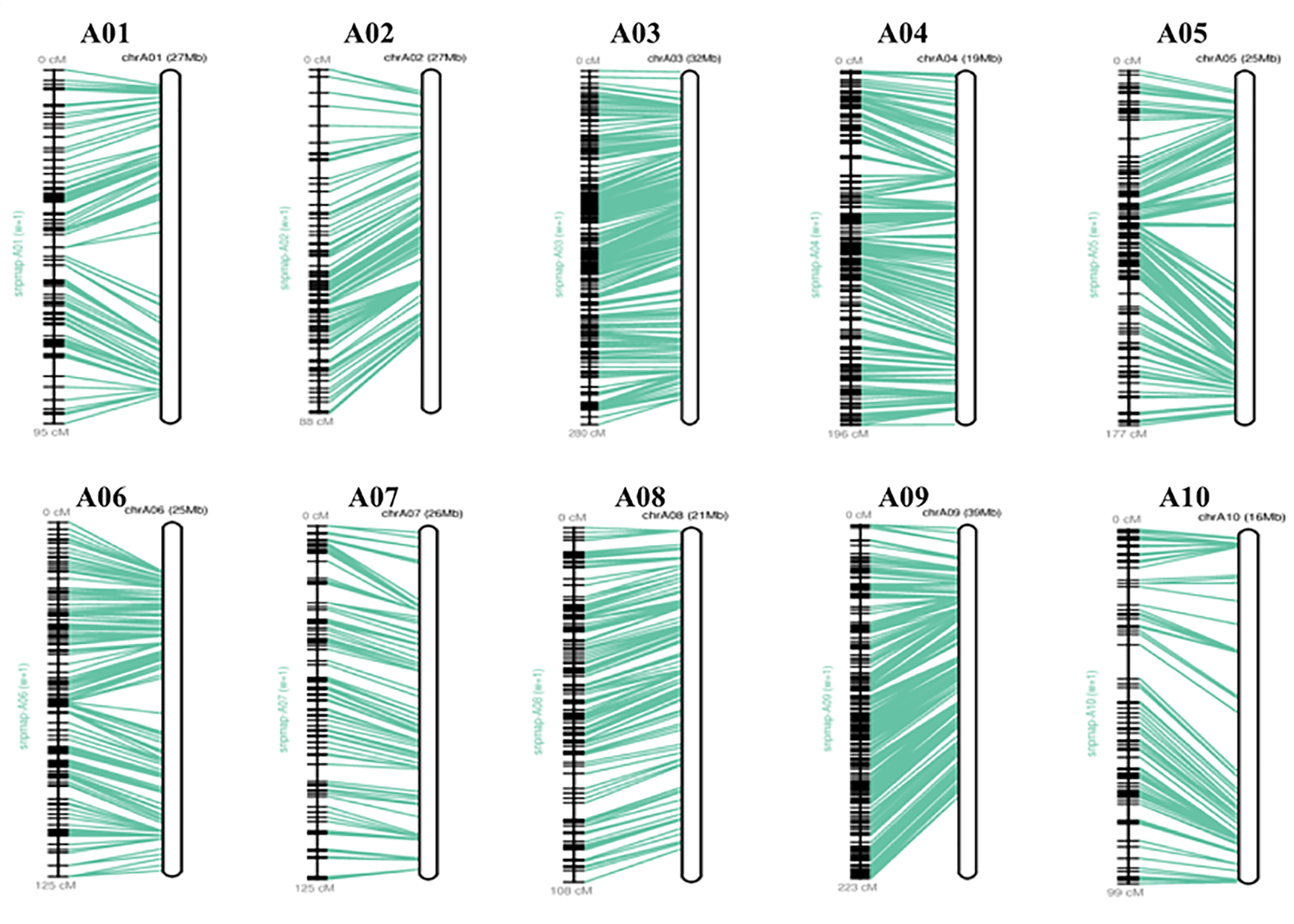

Supplement: Supplementary file 9 — Figure S1 [file 41438_2021_541_MOESM9_ESM.png]

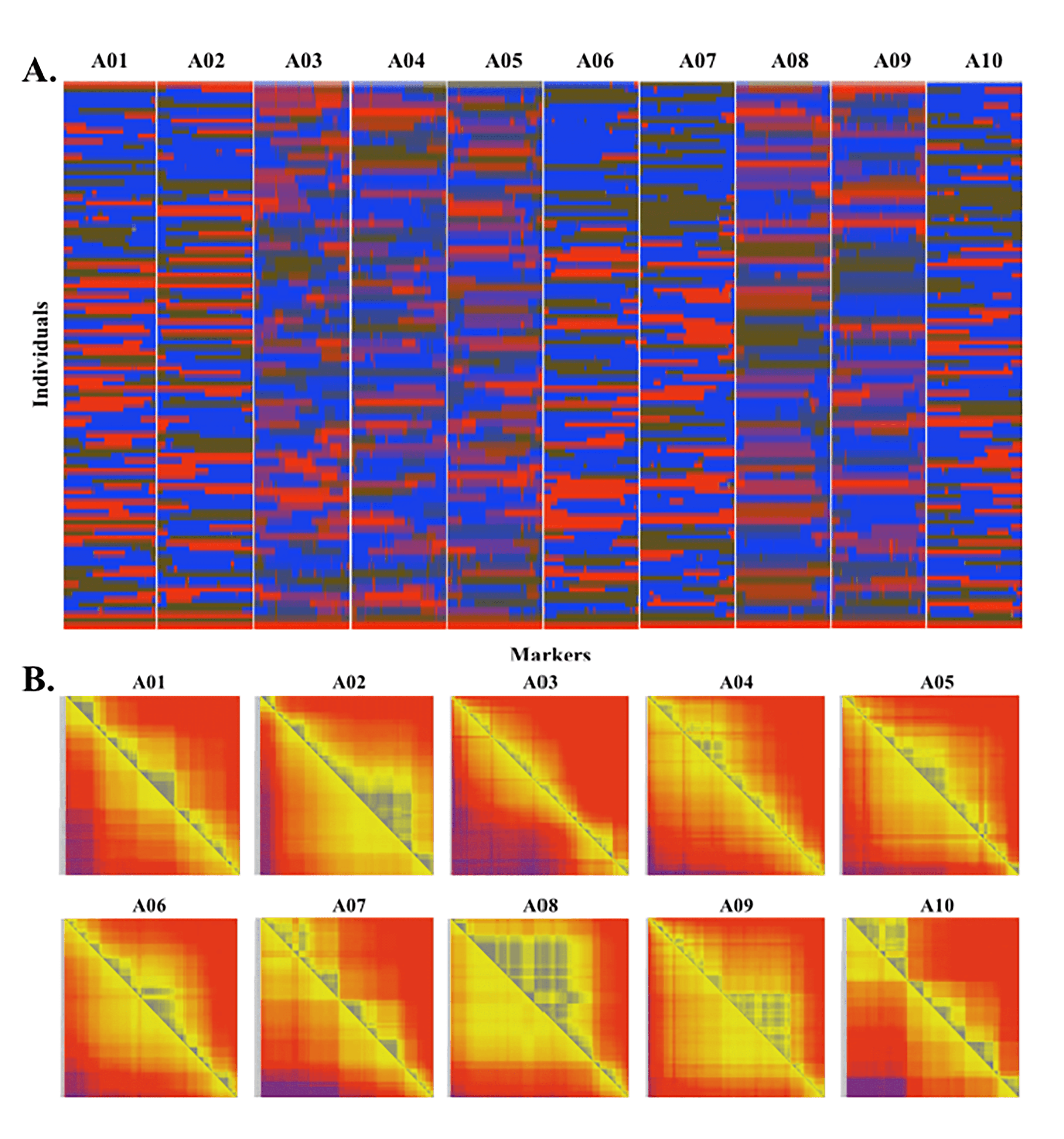

Supplement: Supplementary file 10 — Figure S2 [file 41438_2021_541_MOESM10_ESM.png]

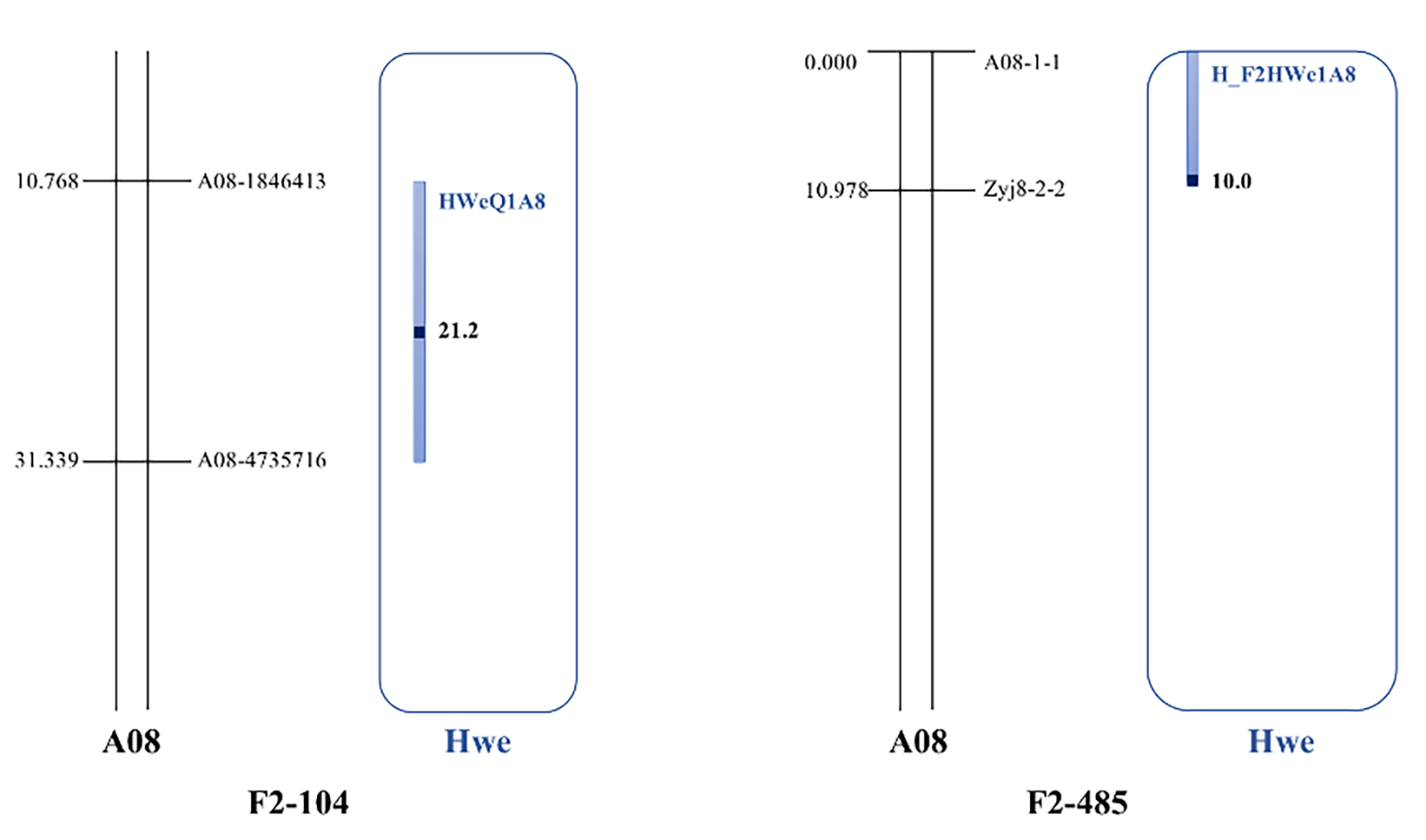

Supplement: Supplementary file 12 — Figure S4 [file 41438_2021_541_MOESM12_ESM.png]
