# Supplementary material for: Genetic analysis of the “head top shape” quality trait of Chinese cabbage and its association with rosette leaf variation
Source: Hortic Res. 2021 May 1;8:106. doi: 10.1038/s41438-021-00541-y (PMC8087666; doi:10.1038/s41438-021-00541-y)
Supplement: Supplementary file 2 — Table S1 [file 41438_2021_541_MOESM2_ESM.pdf]

Table S1. Genetic regulation analysis for HTS by SEA software.

Model	Log_Max_likelihood_Value	AIC	mean[1]	mean[2]	mean[3]	mean[4]	mean[5]	mean[6]	mean[7]	mean[8]	mean[9]	mean[10]	mean[11]	mean[12]	mean[13]	mean[14]	mean[15]	mean[16]
0MG	-1662.509	3329.0183	2.5708															
1MG-A	-1662.505	3331.0102	2.6421	2.4994														
2MG-AI	46252.646	-92495.291	4	3	2	1												
2MG-A	-1662.496	3332.9924	2.7125	2.6183	2.5233	2.4291												
2MG-EA	-1662.505	3331.0106	2.6893	2.5708	2.4523													
2MG-DominanceI	-1662.506	3333.0114	2.6461	2.5084	2.4825													
**2MG-RecessiveI**	**-1376.901**	**2761.8021**	**3.9992**	**2.9999**	**1.8565**													
2MG-Additive	-1609.918	3227.8361	3.6395	2.3752	1.8186													
2MG-Complementa	-1612.272	3230.543	3.6004	2.1778														
2MG-Duplicate	-1662.514	3331.0286	2.5931	2.5039														
2MG-Inhibiting	-1662.514	3331.0286	2.5931	2.5039														
3MG-AI	46252.646	-92487.291	4	4	3	2	3	2	2	1								
3MG-A	-1662.506	3335.0128	2.7007	2.6558	2.5873	2.5423	2.5992	2.5543	2.4858	2.4408								
3MG-CEA	-1662.508	3331.0161	2.7132	2.6183	2.5233	2.4283												
3MG-PEA	-1662.505	3333.0101	2.7245	2.6536	2.6062	2.5353	2.4879	2.417										
4MG-AI	16439.917	-32839.833	1.0001	4	1	1	1.0001	3	1	2	2	2	-0.9999	2	2	1	-0.9999	3
4MG-A	-1662.507	3337.0138	2.5538	2.6331	2.6041	2.6484	2.5532	2.5089	2.6035	2.5353	2.5532	2.509	2.6035	2.5354	2.5757	2.5314	2.626	2.5578
4MG-CEA	-1662.507	3331.0144	2.7366	2.6537	2.5708	2.4879	2.405											
4MG-EEA	-1662.506	3333.0119	2.7241	2.6417	2.6829	2.612	2.5708	2.5295	2.4587	2.4999	2.4174							
4MG-EEEA	-1642.112	3292.2246	3.6014	3.758	3.0089	2.8523	2.2598	2.1031	1.5106	1.7151								

Model	Var(Residual+Polygene	Proportion[1	Proportion[2	Proportion[3	Proportion[4	Proportion[5	Proportion[6	Proportion[7	Proportion[8	Proportion[9	Proportion[10	Proportion[11	Proportion[12	Proportion[13	Proportion[14	Proportion[15	Proportion[16
0MG	0.7459	1															
1MG-A	0.7403	0.5	0.5														
2MG-AI	0	0.1744	0.2976	0.4522	0.0757												
2MG-A	0.7342	0.25	0.25	0.25	0.25												
2MG-EA	0.7384	0.25	0.5	0.25													
2MG-DominanceI	0.7396	0.5	0.25	0.25													
**2MG-RecessiveI**	**0.0653**	**0.1745**	**0.2977**	**0.5278**													
2MG-Additive	0.2857	0.2636	0.4892	0.2473													
2MG-Complementary	0.3408	0.2762	0.7238														
2MG-Duplicate	0.7439	0.75	0.25														
2MG-Inhibiting	0.7439	0.75	0.25														
3MG-AI	0	0.0872	0.0872	0.1488	0.1507	0.1488	0.1507	0.1507	0.0757								
3MG-A	0.7391	0.125	0.125	0.125	0.125	0.125	0.125	0.125	0.125								
3MG-CEA	0.7371	0.125	0.375	0.375	0.125												
3MG-PEA	0.7371	0.125	0.125	0.25	0.25	0.125	0.125										
4MG-AI	0	0	0.1744	0	0.0271	0	0.1524	0	0.064	0.0932	0.0443	0	0.1228	0.1279	0.0485	0	0.1453
4MG-A	0.7436	0.0625	0.0625	0.0625	0.0625	0.0625	0.0625	0.0625	0.0625	0.0625	0.0625	0.0625	0.0625	0.0625	0.0625	0.0625	0.0625
4MG-CEA	0.7385	0.0625	0.25	0.375	0.25	0.0625											
4MG-EEA	0.7382	0.0625	0.0625	0.125	0.125	0.25	0.125	0.125	0.0625	0.0625							
4MG-EEEA	0.3531	0.066	0.0665	0.1754	0.1751	0.1935	0.1976	0.0613	0.0646								

Model	m	da	db	dc	dd	iab(i*)	iac	iad	ibc	ibd	icd	iabc
0MG												
1MG-A	2.5708	0.0714										
2MG-AI	2.5	1	0.5			0						
2MG-A	2.5708	0.0946	0.0471									
2MG-EA	2.5708	0.0593	0.0593									
2MG-DominanceI	2.5708	0.0753	0.0129									
**2MG-RecessiveI**	**2.678**	**0.8215**	**0.4997**									
2MG-Additive	2.5521	0.4552				0.177						
2MG-Complementary	2.8891					0.7113						
2MG-Duplicate	2.5485					0.0446						
2MG-Inhibiting	2.5485					-0.0446						
3MG-AI	2.625	0.625	0.375	0.625		-0.125	0.125		-0.125			-0.125
3MG-A	2.5708	0.0567	0.0225	0.0508								
3MG-CEA	2.5708	0.0475	0.0475	0.0475								
3MG-PEA	2.5708	0.0592	0.0592	0.0355								
4MG-AI	1.5	0.25	0	0.5	0.25	0	0	-0.75	0.25	-0.25	-0.75	
4MG-A	2.5708	0.0093	0.0093	-0.0185	-0.0067							
4MG-CEA	2.5708	0.0414	0.0414	0.0414	0.0414							
4MG-EEA	2.5708	0.0561	0.0561	0.0206	0.0206							
4MG-EEEA	2.6012	0.3475	0.3475	0.3475	-0.0332							

Model	Major-Gene Var	Heritability(Major-	U1	P(U1)	U2	P(U2)	U3	P(U3)	nW	P(nW)	Dn	P(Dn)
0MG			1.8956	0.1686	0.2784	0.5977	10.3796	0.0013	15.3068	0.044	0.049	0.0036
1MG-A	0.0057	0.0076	1.8988	0.1682	0.2746	0.6002	10.5011	0.0012	15.3131	0.044	0.0489	0.0037
2MG-AI	0.7459	1	0.129	0.7195	1.4864	0.2228	39.2859	0	13.709	0.0362	0.0872	0
2MG-A	0.0117	0.0157	1.8985	0.1682	0.2747	0.6002	10.496	0.0012	15.3128	0.044	0.0489	0.0037
2MG-EA	0.0076	0.0102	1.8988	0.1682	0.2746	0.6002	10.5012	0.0012	15.3131	0.044	0.0489	0.0037
2MG-DominanceI	0.0063	0.0085	1.8989	0.1682	0.2747	0.6002	10.501	0.0012	15.3131	0.044	0.0489	0.0037
**2MG-RecessiveI**	**0.6807**	**0.9125**	**14.9006**	**0.0001**	**2.2537**	**0.1333**	**80.0189**	**0**	**16.9797**	**0.052**	**0.087**	**0**
2MG-Additive	0.4602	0.617	0.0558	0.8132	0.0038	0.9508	1.35	0.2453	14.4304	0.0397	0.0665	0
2MG-Complementary	0.4052	0.5432	0.0085	0.9266	0.0417	0.8382	1.3771	0.2406	14.4677	0.0399	0.0688	0
2MG-Duplicate	0.0021	0.0028	1.8996	0.1681	0.2748	0.6001	10.5046	0.0012	15.3134	0.044	0.0489	0.0037
2MG-Inhibiting	0.0021	0.0028	1.8996	0.1681	0.2748	0.6001	10.5046	0.0012	15.3134	0.044	0.0489	0.0037
3MG-AI	0.7459	1	0.129	0.7195	1.4864	0.2228	39.2859	0	13.709	0.0362	0.0872	0
3MG-A	0.0069	0.0092	1.8988	0.1682	0.2746	0.6002	10.5018	0.0012	15.3132	0.044	0.0489	0.0037
3MG-CEA	0.0088	0.0119	1.9076	0.1672	0.2646	0.607	10.8348	0.001	15.3303	0.0441	0.0487	0.0039
3MG-PEA	0.0088	0.0118	1.8988	0.1682	0.2746	0.6002	10.5011	0.0012	15.3131	0.044	0.0489	0.0037
4MG-AI	0.7459	1	0.0003	0.9866	1.5032	0.2202	24.6944	0	13.5675	0.0355	0.0861	0
4MG-A	0.0023	0.0031	1.8984	0.1683	0.2748	0.6001	10.4937	0.0012	15.3127	0.044	0.0489	0.0037
4MG-CEA	0.0074	0.01	1.8988	0.1682	0.2746	0.6002	10.5023	0.0012	15.3132	0.044	0.0489	0.0037
4MG-EEA	0.0077	0.0103	1.8988	0.1682	0.2746	0.6002	10.5016	0.0012	15.3131	0.044	0.0489	0.0037
4MG-EEEA	0.3928	0.5266	0.9348	0.3336	0.3008	0.5834	2.4053	0.1209	14.6242	0.0407	0.0546	0.0008

Model	Log_Max_likelihood_Value	AIC	mean[1]	mean[2]	mean[3]	mean[4]	mean[5]	mean[6]	mean[7]	mean[8]	mean[9]	mean[10]	mean[11]	mean[12]	mean[13]	mean[14]	mean[15]	mean[16]
0MG	-3124.665	6253.3292	6.6968															
1MG-A	-3124.66	6255.3203	6.9158	6.4778														
2MG-AI	-2511.957	5033.9148	10.8789	8.1595	4.9053	2.0517												
2MG-A	-3124.65	6257.3009	7.1317	6.8427	6.5509	6.2619												
2MG-EA	-3124.66	6255.3208	7.0604	6.6968	6.3332													
2MG-DominanceI	-3124.659	6257.3184	6.9417	6.4967	6.4071													
2MG-RecessiveI	-2963.927	5935.8532	10.6203	8.1372	4.5166													
2MG-Additive	-3049.666	6107.3311	9.7638	5.8194	4.2596													
2MG-Complementa	-3049.842	6105.6848	9.6924	5.2537														
2MG-Duplicate	-3124.671	6255.3421	6.7811	6.4438														
2MG-Inhibiting	-3124.671	6255.3421	6.7811	6.4438														
3MG-AI	-2511.937	5041.8739	10.8757	10.8756	4.9045	4.9045	8.1593	4.9045	4.9045	2.0451								
3MG-A	-3124.662	6259.3232	7.096	6.958	6.7474	6.6095	6.7841	6.6462	6.4356	6.2976								
3MG-CEA	-3124.663	6255.3263	7.1338	6.8424	6.5511	6.2598												
3MG-PEA	-3124.66	6257.3203	7.1686	6.9511	6.8056	6.588	6.4425	6.225										
**4MG-AI**	**-1728.946**	**3497.8923**	**10.4637**	**10.9529**	**8.0507**	**8.9178**	**7.9189**	**7.1332**	**2.9281**	**5.0701**	**4.9135**	**5.0856**	**3.8755**	**5.0596**	**5.0617**	**3.8543**	**1.3412**	**3.9048**
4MG-A	-3124.652	6261.3049	7.007	6.9561	7.007	7.1235	6.947	6.8305	6.947	6.9951	6.4683	6.3518	6.4683	6.5164	6.3998	6.2834	6.3998	6.448
4MG-CEA	-3124.663	6255.325	7.2054	6.9511	6.6968	6.4425	6.1881											
4MG-EEA	-3124.661	6257.3222	7.1678	6.9145	7.0412	6.8234	6.6968	6.5702	6.3524	6.479	6.2258							
4MG-EEEA	-3103.623	6215.2451	9.8667	10.34	8.0233	7.5501	5.7067	5.2335	3.3901	4.2107								

Model	Var(Residual+Polygene)	Proportion[1	Proportion[2	Proportion[3	Proportion[4	Proportion[5	Proportion[6	Proportion[7	Proportion[8	Proportion[9	Proportion[10	Proportion[1	Proportion[1	Proportion[1	Proportion[14	Proportion[15	Proportion[16
0MG	7.0145	1															
1MG-A	6.9612	0.5	0.5														
2MG-AI	0.2417	0.174	0.2976	0.4526	0.0758												
2MG-A	6.9039	0.25	0.25	0.25	0.25												
2MG-EA	6.943	0.25	0.5	0.25													
2MG-DominanceI	6.9482	0.5	0.25	0.25													
2MG-RecessiveI	0.9151	0.1949	0.2736	0.5315													
2MG-Additive	2.3448	0.3133	0.4568	0.2299													
2MG-Complementary	2.6863	0.3251	0.6749														
2MG-Duplicate	6.9878	0.75	0.25														
2MG-Inhibiting	6.9878	0.75	0.25														
3MG-AI	0.2438	0.0871	0.0871	0.1133	0.1133	0.2971	0.1133	0.1133	0.0753								
3MG-A	6.9497	0.125	0.125	0.125	0.125	0.125	0.125	0.125	0.125								
3MG-CEA	6.9317	0.125	0.375	0.375	0.125												
3MG-PEA	6.9314	0.125	0.125	0.25	0.25	0.125	0.125										
**4MG-AI**	**0.0245**	**0.0211**	**0.1527**	**0.0929**	**0.0835**	**0.0856**	**0.036**	**0.0322**	**0.0818**	**0.0764**	**0.079**	**0.0159**	**0.0837**	**0.0833**	**0.0142**	**0.0436**	**0.0182**
4MG-A	6.9255	0.0625	0.0625	0.0625	0.0625	0.0625	0.0625	0.0625	0.0625	0.0625	0.0625	0.0625	0.0625	0.0625	0.0625	0.0625	0.0625
4MG-CEA	6.9445	0.0625	0.25	0.375	0.25	0.0625											
4MG-EEA	6.9418	0.0625	0.0625	0.125	0.125	0.25	0.125	0.125	0.0625	0.0625							
4MG-EEEA	3.3095	0.0674	0.0663	0.1777	0.1751	0.1915	0.1959	0.0612	0.0649								

Model	m	da	db	dc	dd	iab(i*)	iac	iad	ibc	ibd	icd	iabc
0MG												
1MG-A	6.6968	0.219										
2MG-AI	6.4988	3.0204	1.3932			-0.0335						
2MG-A	6.6968	0.2904	0.1445									
2MG-EA	6.6968	0.1818	0.1818									
2MG-DominanceI	6.6968	0.2449	0.0448									
2MG-RecessiveI	6.9477	2.431	1.2416									
2MG-Additive	6.4155	1.376				0.5961						
2MG-Complementary	7.4731					2.2193						
2MG-Duplicate	6.6125					0.1687						
2MG-Inhibiting	6.6125					-0.1687						
3MG-AI	6.4467	2.2571	0.7643	1.4434		0.0494	0.7285		-0.7643			-0.0494
3MG-A	6.6968	0.1743	0.069	0.1559								
3MG-CEA	6.6968	0.1457	0.1457	0.1457								
3MG-PEA	6.6968	0.1815	0.1815	0.1088								
**4MG-AI**	**5.9082**	**1.7712**	**1.2567**	**1.0147**	**0.5055**	**0.6602**	**0.423**	**-0.0923**	**-0.3257**	**-0.3318**	**-0.3391**	
4MG-A	6.6968	0.2798	0.0405	-0.0413	0.0413							
4MG-CEA	6.6968	0.1272	0.1272	0.1272	0.1272							
4MG-EEA	6.6968	0.1722	0.1722	0.0633	0.0633							
4MG-EEEA	6.7901	1.0613	1.0613	1.0613	-0.0749							

Model	Major-Gene Var	Heritability(Major-Gene)	U1	P(U1)	U2	P(U2)	U3	P(U3)	nW	P(nW)	Dn	P(Dn)
0MG			1.3171	0.2511	0.0001	0.994	19.4917	0	10.9289	0.023	0.0521	0.0016
1MG-A	0.0533	0.0076	1.3195	0.2507	0	0.9972	19.6684	0	10.9357	0.023	0.052	0.0016
2MG-AI	6.7728	0.9655	0.404	0.525	0.0162	0.8986	8.8275	0.003	8.2724	0.0116	0.0701	0
2MG-A	0.1106	0.0158	1.3193	0.2507	0	0.9971	19.6608	0	10.9353	0.023	0.052	0.0016
2MG-EA	0.0715	0.0102	1.3195	0.2507	0	0.9972	19.6685	0	10.9357	0.023	0.052	0.0016
2MG-DominanceI	0.0664	0.0095	1.3197	0.2507	0	0.9971	19.6673	0	10.9357	0.023	0.052	0.0016
2MG-RecessiveI	6.0994	0.8695	5.7099	0.0169	0.8386	0.3598	31.2662	0	9.4174	0.0163	0.0678	0
2MG-Additive	4.6697	0.6657	0.0236	0.8779	0.0041	0.9492	0.1156	0.7339	9.1329	0.0151	0.0659	0
2MG-Complementary	4.3282	0.617	0.001	0.9749	0.006	0.9383	0.0352	0.8511	9.0762	0.0148	0.0692	0
2MG-Duplicate	0.0267	0.0038	1.3206	0.2505	0	0.997	19.6755	0	10.9362	0.023	0.052	0.0016
2MG-Inhibiting	0.0267	0.0038	1.3206	0.2505	0	0.997	19.6755	0	10.9362	0.023	0.052	0.0016
3MG-AI	6.7708	0.9653	0.412	0.5209	0.0141	0.9054	8.7692	0.0031	8.2685	0.0116	0.0698	0
3MG-A	0.0648	0.0092	1.3195	0.2507	0	0.9972	19.6695	0	10.9358	0.023	0.052	0.0016
3MG-CEA	0.0828	0.0118	1.3261	0.2495	0	0.9945	20.1369	0	10.9538	0.0231	0.0519	0.0017
3MG-PEA	0.0831	0.0118	1.3195	0.2507	0	0.9972	19.6683	0	10.9357	0.023	0.052	0.0016
**4MG-AI**	**6.99**	**0.9965**	**1.473**	**0.2249**	**0.7415**	**0.3892**	**1.5781**	**0.209**	**8.8536**	**0.0139**	**0.0583**	**0.0003**
4MG-A	0.0891	0.0127	1.3192	0.2507	0	0.9972	19.6622	0	10.9354	0.023	0.052	0.0016
4MG-CEA	0.0701	0.01	1.3196	0.2507	0	0.9972	19.6702	0	10.9358	0.023	0.052	0.0016
4MG-EEA	0.0727	0.0104	1.3195	0.2507	0	0.9972	19.6691	0	10.9357	0.023	0.052	0.0016
4MG-EEEA	3.705	0.5282	0.5787	0.4468	0.0121	0.9123	6.2782	0.0122	10.0776	0.0191	0.0583	0.0003
