# Supplementary material for: Genetic analysis of the “head top shape” quality trait of Chinese cabbage and its association with rosette leaf variation
Source: Hortic Res. 2021 May 1;8:106. doi: 10.1038/s41438-021-00541-y (PMC8087666; doi:10.1038/s41438-021-00541-y)
Supplement: Supplementary file 3 — Table S2 [file 41438_2021_541_MOESM3_ESM.pdf]

Table S2. Paired samples t-test for leaf and leafy head traits

Trait		Average value	SD	t	Sig.
**OLW**	N - OC	-6.627133	6.806325	-3.771	0.002
	N - O	-6.4742	4.579209	-5.476	0
	OC - O	0.152933	8.463213	0.07	0.945
**OLL**	N - OC	-10.218133	6.436652	-6.148	0
	N - O	-14.814333	3.853687	-14.889	0
	OC - O	-4.5962	7.385006	-2.41	0.03
**OLaL**	N - OC	-7.190133	4.788035	-5.816	0
	N - O	-9.14	4.052362	-8.735	0
	OC - O	-1.949867	7.032998	-1.074	0.301
**OLvL**	N - OC	-0.80912	4.00591	-0.833	0.417
	N - O	-4.07465	3.15604	-5.323	0.000
	OC - O	-2.26673	3.32180	-3.738	0.001
**OLPW**	N - OC	-1.8062	0.784163	-8.921	0
	N - O	-1.712467	0.879103	-7.544	0
	OC - O	0.093733	1.073401	0.338	0.74
**OLPL**	N - OC	-0.8954	4.25793	-0.814	0.429
	N - O	-3.934467	3.341851	-4.56	0
	OC - O	-3.039067	2.843966	-4.139	0.001
**HLW**	OC - O	-0.153467	3.238955	-0.184	0.857
**HLL**	OC - O	-2.425667	5.924732	-1.586	0.135
**HLaL**	OC - O	-1.457667	7.726027	-0.731	0.477
**HLvL**	OC-O	-2.33616	3.64262	-3.207	0.004
**HLPW**	OC - O	-0.746933	2.938563	-0.984	0.342
**HLPL**	OC - O	-1.515933	5.017147	-1.17	0.261
**OLA**	N - OC	-309.94127	160.515892	-7.478	0
	N - O	-199.5614	237.410162	-3.256	0.006
	OC - O	110.379867	271.27918	1.576	0.137
**OLPA**	N - OC	-34.855333	19.997284	-6.751	0
	N - O	-42.696333	22.315404	-7.41	0
	OC - O	-7.841	31.220402	-0.973	0.347
**PH**	N - OC	-14.037000	6.320689	-9.157	.000
	N - O	-13.902059	2.928617	-19.572	.000
	OC - O	1.461033	5.933749	1.349	.188
**PW**	N - OC	2.49424	3.68674	2.789	0.013
	N - O	2.19400	3.98547	2.270	0.037
	OC - O	1.40690	4.47319	1.723	0.096
**PWe**	N - OC	-2.678933	0.56631	-18.321	0
	N - O	-2.465467	0.909158	-10.503	0
	OC - O	0.213467	1.071595	0.772	0.453
**HWe**	OC - O	0.019467	0.723094	0.104	0.918

Significant at the 0.05 level (2-tailed).
