# Supplementary material for: Genetic analysis of the “head top shape” quality trait of Chinese cabbage and its association with rosette leaf variation
Source: Hortic Res. 2021 May 1;8:106. doi: 10.1038/s41438-021-00541-y (PMC8087666; doi:10.1038/s41438-021-00541-y)
Supplement: Supplementary file 4 — Table S3 [file 41438_2021_541_MOESM4_ESM.pdf]

Table S3. Characteristics of F2-104 genetic map

Linkage group	Number of markers	Length (cM)	Density (cM)	Maximum interval size (cM)
A01	171	95.88	0.56	4.83
A02	193	88.49	0.46	1.69
A03	571	280.91	0.49	1.34
A04	413	196.93	0.48	0.94
A05	337	177.65	0.53	4.10
A06	330	125.94	0.38	2.47
A07	183	125.86	0.69	2.63
A08	361	108.28	0.30	1.28
A09	454	223.52	0.49	1.57
A10	181	99.44	0.55	2.72
Sum/mean	3194	1522.89	0.48	4.83
