# Supplementary material for: Genetic analysis of the “head top shape” quality trait of Chinese cabbage and its association with rosette leaf variation
Source: Hortic Res. 2021 May 1;8:106. doi: 10.1038/s41438-021-00541-y (PMC8087666; doi:10.1038/s41438-021-00541-y)
Supplement: Supplementary file 7 — Table S6 [file 41438_2021_541_MOESM7_ESM.pdf]

Table S6. LOD threshold value for QTL analysis with IM and CIM methods

Trait	IM LOD Threshold	CIM LOD Threshold	Trait	IM LOD Threshold	CIM LOD Threshold
**HTS**	3.5447	4.0279			
**OLL**	3.4663	3.9494	**HLL**	3.4929	4.0652
**OLaL**	3.6718	4.0484	**HLaL**	3.5275	4.1344
**OLvL**	3.5543	3.9902	**HLvL**	3.5664	4.0178
**OLW**	3.5412	4.0785	**HLW**	3.5945	4.1101
**OLA**	3.5408	3.8992	**HLA**	3.4478	4.0157
**OLPL**	3.4556	3.9984	**HLPL**	3.6145	4.1059
**OLPW**	3.4538	4.0307	**HLPW**	3.5503	4.0172
**OLPA**	3.5375	4.0340	**HLPA**	3.7510	4.0176
**PWe**	3.6009	3.9926	**PH**	3.4356	3.9598
**HWe**	3.6155	4.0279	**PW**	3.4882	3.9600
