# Supplementary material for: Genetic analysis of the “head top shape” quality trait of Chinese cabbage and its association with rosette leaf variation
Source: Hortic Res. 2021 May 1;8:106. doi: 10.1038/s41438-021-00541-y (PMC8087666; doi:10.1038/s41438-021-00541-y)
Supplement: Supplementary file 8 — Table S7 [file 41438_2021_541_MOESM8_ESM.pdf]

Gene ID	OC-seedling (1)	OC-seedling (2)	OC-rosette (1)	OC-rosette (2)	OC-heading (1)	OC-heading (2)	O-seedling (1)	O-seedling (2)	O-rosette (1)	O-rosette (2)	O-heading (1)	O-heading (2)	N-seedling (1)	N-seedling (2)	N-rosette-1 (1)	N-rosette-1 (2)	N-rosette-2 (1)	N-rosette-2 (2)
Bra000168	6.024164641	6.047903783	5.87444683	5.97004421	5.91496803	5.97508992	6.03119536	6.02080328	6.0452403	5.9454257	5.7375361	5.9620254	5.94845117	6.06259912	6.083269238	5.906600331	6.057209217	6.071643822
Bra000223	6.700454553	6.440529307	6.54023351	6.55470811	6.5070186	6.57049406	6.50296453	6.32100846	6.5567089	6.6932184	6.5504643	6.5165359	6.47721133	6.3820028	6.166476838	6.217193724	6.427276742	6.670330448
Bra000230	11.74802144	11.47457736	12.454084	12.4772436	12.7400783	12.8261599	12.1277641	12.2144591	12.549655	12.714399	13.083849	13.025233	11.8015432	11.9937001	11.74848137	12.76799701	11.38358734	13.30892845
Bra000288	10.10971883	10.27872978	10.0433805	10.0386849	10.1618787	9.91804206	10.4166288	10.4401544	10.489362	10.761189	10.352677	10.342145	10.5485185	10.8571049	10.77528219	10.93109416	10.17752578	10.09329299
Bra000289	6.345917039	6.385562309	6.46225051	6.39521172	6.60533251	6.61299267	6.13679577	6.07604585	6.1759121	6.2706484	6.1983214	6.051976	6.30181941	6.1885128	6.497177523	6.32173784	6.417109882	5.947636955
Bra000329	6.089154505	6.194680325	6.25193238	6.14034564	6.28556904	6.05219824	6.15912164	6.13565333	6.0311554	6.1684007	6.2074746	6.1099825	6.30425708	6.25167718	6.267629046	6.181546699	6.568906054	6.545368773
Bra000330	6.00636658	5.921958759	5.96127164	5.98536147	6.13921151	6.08695893	6.06712348	6.03137596	5.9329367	6.0503979	6.5017021	6.2416335	5.91067138	6.04716431	6.074371962	6.079302481	6.030448233	6.023368951
Bra000423	6.153892	6.153126327	6.20127167	6.0275555	6.20780936	6.20365786	6.05498549	6.08785016	6.006841	5.9616871	6.2201819	6.012816	6.00210277	6.04822629	6.143819069	6.088518417	6.23778595	6.211748624
Bra000861	6.74335738	6.635815516	6.43621968	6.66622415	6.26215537	6.28574196	6.44259533	6.36882314	6.3981417	6.2686278	6.2464287	6.1873281	6.52834393	6.44623775	6.032508809	6.128083406	6.380544337	6.670961348
Bra001150	9.002345837	9.072997875	8.96901034	9.04165243	9.10627533	8.73704617	8.95359785	8.97184235	8.9131718	8.9406147	9.0735684	8.9771758	9.16833266	8.92905465	9.014825942	8.982970518	9.495405604	9.376139869
Bra001163	6.420565809	6.415302211	6.31369357	6.27572314	6.2778743	6.33398793	6.47970566	6.6261347	6.624693	6.587378	6.4755107	6.439993	6.63057376	6.43401692	6.682844329	6.355568157	6.604642486	6.526930172
Bra001197	6.323096913	6.163783608	6.81670375	6.82871629	7.3802637	7.05623748	6.34766042	6.51692236	6.9446238	6.8063581	8.1706449	7.5946398	5.85268389	6.19402179	6.582184063	6.52013068	6.53319988	6.268206419
Bra001269	6.216931791	6.153604223	6.20126267	6.10390152	6.00994862	6.17740589	5.96878042	6.05974652	5.8410034	6.0132665	6.0537006	5.727476	5.87994254	6.18195514	6.102155063	5.903882364	6.057243005	6.010026251
Bra001296	6.335611941	6.18469281	6.29453054	6.30250531	6.28628615	6.29162403	6.41758686	6.30274787	6.3173532	6.2895562	6.3400281	6.3054721	6.47565383	6.38653931	6.307467384	6.285688206	6.463295696	6.467027664
Bra001297	6.221243465	6.12015553	6.2988653	6.15398356	6.10801153	5.96254993	6.28057614	6.37793482	6.3587005	6.4271225	6.4014223	6.4265197	6.2321445	6.41473365	6.313922466	6.338799157	6.605313214	6.40383526
Bra002001	9.862851462	10.00210345	10.0019748	10.1351402	10.5621445	10.5849884	10.5915278	10.2606211	11.013744	10.361816	10.614328	10.244623	9.58643557	9.42334418	9.36000879	9.257571416	9.648760799	11.42730381
Bra002015	6.2895511	6.144041744	6.23485837	6.20184402	6.03459135	6.17771962	6.24264341	6.26126451	6.1031358	6.2891047	6.1530417	6.1929777	6.28940815	6.28627706	6.212043721	6.141762581	6.304948448	6.273322928
Bra002016	5.831742375	5.88396767	5.81671589	5.85623155	5.76782102	5.75534775	5.81895773	5.72225905	5.6745209	5.7436324	5.8036097	5.8079251	5.81221454	5.78539168	5.736009619	5.795651335	5.848324148	5.798553444
Bra002017	6.404914083	6.471159775	6.68637726	6.55862341	6.53977614	6.40368294	6.01829764	6.29745095	6.1661767	6.1268997	6.0844047	5.9871308	6.30374056	6.27103964	6.507789023	6.299576044	6.828161899	6.375636018
Bra002020	5.995379895	6.046833272	6.15888698	5.96051399	5.76093632	6.29024589	5.93617871	5.95671466	5.9769067	6.171775	6.2022815	6.1664431	6.05362439	5.94123563	5.957330202	6.053384287	6.1174401	6.001966361
Bra002124	9.207651891	9.2493141	8.72417146	8.77534431	9.09442668	7.47836974	9.81005299	9.37231151	8.8028979	9.2658065	8.1846346	8.1358939	8.25945039	8.79935326	8.589349436	8.227654842	8.123397995	7.981468635
Bra002126	8.6813278	10.72652939	9.96946095	10.1368208	10.2666854	8.51720386	10.724687	10.8812232	10.615239	10.870035	9.6065824	9.4600474	10.1521301	10.5855361	10.24404331	9.834494641	9.198179315	8.886582769
Bra002134	6.318920789	6.459018011	6.53071717	6.22150495	6.65251292	6.42527845	6.2376538	6.16159221	6.440655	6.5176944	6.5440863	6.5462624	6.56597466	6.62091628	6.859604281	6.747268356	6.365420453	6.540450564
Bra002173	7.082816134	7.226000095	7.1218203	7.04265694	7.11919552	7.01397021	7.26037479	7.54711099	7.4554607	7.332582	7.0503654	7.1992937	7.29246019	7.60491539	7.143221697	7.20839535	6.528042855	6.83159384
Bra002654	7.428709909	7.539038441	7.06065541	7.75094857	7.1062135	7.4874825	8.05961599	7.42841072	8.0850509	7.2512225	8.0668146	7.772163	8.20103135	8.03945437	8.519987751	8.014585074	9.12852385	8.774952964
Bra002655	9.639549391	9.562195503	10.1837947	10.0543834	10.8334334	10.9051703	10.1086045	10.0635724	10.394067	10.394274	10.747031	10.641469	9.43896947	9.69854401	10.15731155	9.964319686	10.81895228	10.53855978
Bra002766	6.824197448	6.578674914	6.5111964	6.76486759	6.49991653	6.53668507	6.72562716	6.55412255	6.5409553	6.5525775	6.6587149	6.5893932	7.06461739	6.88195917	6.776510933	6.455557564	7.324721995	7.019929902
Bra002782	6.007173691	6.020680571	6.36585214	6.02292691	6.06701249	6.02786629	6.82086859	7.52194867	8.5191781	8.8735316	8.8099079	8.9437291	7.93553884	7.77663504	9.101161035	9.34512915	9.833083868	10.02884217
Bra003057	7.391812386	7.147423257	6.86041537	6.63005896	7.57265031	6.87914626	7.25640822	7.11383104	6.4252832	6.2279336	6.5378198	6.7182461	7.36978836	7.54420106	7.506579383	7.6684067	6.549358106	6.303657155
Bra003058	6.014316442	5.906547223	6.04138717	5.9630615	5.9362052	5.85125231	5.87284781	5.82755006	6.0718204	5.8682162	5.8830615	5.907743	6.00213085	5.97836922	5.892435616	5.949900077	6.177916361	6.037529399
Bra003067	6.168030091	5.980424838	6.12994619	6.12618255	6.14827567	6.10553345	6.27426684	6.29939384	6.2472058	6.1679593	6.2174263	6.1565972	6.30745196	6.30665715	6.135988987	6.108532052	6.52216044	6.455524104
Bra003154	8.67574026	8.819667914	7.79863346	8.48795062	7.5381801	8.23317471	7.3185096	7.00122611	7.0099903	7.0673984	7.1675165	6.9562984	7.63246172	7.51665749	6.694803554	7.187941145	6.614235959	7.802076342
Bra003187	6.561552143	6.444616998	6.13827017	6.21257324	6.14113558	6.24016001	7.42776645	7.37357369	6.1242053	6.2087981	6.2006547	6.2267875	7.15005979	6.96222405	7.575767704	7.144378933	7.172119304	7.142591625
Bra003201	7.997624577	7.865462893	7.80344106	7.63369719	7.3208563	7.40045834	7.6664703	7.3823301	7.3538694	6.9448106	7.1662779	6.9295549	8.11391757	7.9100531	8.123031431	7.573638698	9.322872965	9.154373969
Bra003202	6.341285172	6.226554345	6.39313884	6.33086731	6.30717419	6.3634622	7.28195023	7.28158918	6.9924738	7.0842033	6.8893122	6.9897553	7.79024952	7.50476712	7.467519285	7.176672296	7.523516271	7.221340785
Bra003287	6.609995318	6.491137361	7.85860255	6.96404495	6.37269803	6.79587287	7.20478811	7.08098427	7.4048768	6.8398306	7.2227422	6.9311746	7.26315905	6.86980336	7.323484336	6.646285631	9.481229943	9.438352627
Bra003294	6.294976728	6.499208162	6.23644324	6.39210963	6.2532709	6.19455554	6.1722464	6.22032639	6.179421	6.2248783	6.2997827	6.1678435	6.6845868	6.24888827	6.296293651	6.320265074	6.161974373	6.110054999
Bra003295	6.16408021	6.171679697	6.08706406	6.01051704	6.33352151	6.09126324	6.27120388	6.15063569	6.3281265	6.2900261	6.2248152	6.1919041	6.18895017	6.27739454	6.274095473	6.279084736	6.256888734	6.264852394
Bra003330	7.782839291	8.168893825	6.87530487	8.12472624	7.13913283	7.23922205	6.81764098	6.68664058	6.7387559	6.462176	6.661862	6.4715151	8.06593671	8.69141864	8.367504845	7.600924334	10.17172145	10.00002596
Bra003331	6.310147167	6.144780718	6.21497437	6.25559729	6.11303676	6.18125316	6.10984644	5.98113894	5.8921847	6.0091473	5.9173808	5.9299325	5.97657238	5.92660781	6.054965961	6.11562316	5.921603901	5.989196012
Bra003364	6.161956112	6.220861455	6.01678485	6.3242715	5.99934794	6.19417762	5.84211295	5.93358829	5.8938851	5.9853274	5.9025221	5.9310339	5.93685635	5.75533245	5.937667392	6.052309532	5.921921757	5.891621097
Bra003458	6.858426876	6.832664277	6.48257111	6.36208061	6.98687819	6.29316339	6.14952668	6.0732859	6.2530904	6.3159039	6.3229717	6.2732153	6.81274739	6.84642453	6.711875281	6.82970686	6.316935315	6.381047
Bra003461	7.065350976	7.107856209	6.66371281	6.6453035	6.99204874	6.7624494	6.90952797	6.9591588	6.9254558	6.7719498	6.8734636	7.0300264	7.24397576	7.25299789	7.126515656	7.330517021	7.279921163	7.154446331
Bra003469	6.072307984	6.121341842	6.22210282	6.15515261	6.17172066	6.17201964	6.08085158	5.97150194	6.1471155	6.1724886	6.3054691	6.3117346	6.29820849	6.59486835	6.42030506	6.481520771	5.981449805	6.074187815
Bra003474	6.449092374	6.630022059	6.47996084	6.60556226	6.42034725	6.32609928	6.8506909	6.55180885	6.9330124	6.5763972	6.4832104	6.6035305	6.6052315	6.54307287	6.379008251	6.642937871	6.477236575	6.654377443
Bra003478	5.957061517	6.022479473	5.84977905	5.95511775	5.92811339	5.96660933	5.87068783	6.0039266	5.9033245	6.0254438	6.0752864	5.8905333	5.83614719	5.84643906	6.127387124	5.870063586	5.8162176	5.807295936
Bra003483	6.614791063	6.521220355	7.21863182	7.51642568	7.67917885	7.28050186	6.78741036	6.57370356	7.5025908	7.4703063	8.0941617	7.7742384	6.73431112	6.69812775	7.15696858	7.647915028	7.004531607	8.064411087
Bra003484	7.730129915	7.637429565	9.44835592	9.05325024	7.9038266	7.80184539	7.02291168	7.20167639	7.611255	7.4039617	7.4753721	7.4569891	7.78561743	7.57246258	7.610877706	7.664299407	7.535289658	7.163052982
Bra003486	6.315610498	6.388715623	6.16103961	6.65757371	6.28384119	6.33651249	5.90850334	5.93696607	6.1080614	6.0472786	5.9693509	6.0302202	6.05412318	6.17504148	6.139793574	6.26539	6.049948184	6.273733593
Bra003495	5.964399633	6.060367606	5.90993167	5.91362577	5.98636201	5.88415116	6.11465875	6.05144688	6.0530893	5.9442339	5.9875809	6.0670114	5.88807037	6.02553545	5.896023932	5.999827102	6.001147184	5.962554436
Bra003531	10.75475545	10.54472893	10.6162464	10.1217378	10.2211839	9.86610359	11.2821618	10.5373899	10.496141	10.445569	10.347214	10.203231	10.1100018	10.3157494	10.31326339	9.865286539	9.807715589	9.33354906
Bra003542	5.984474436	6.161608791	6.04835445	6.16629551	6.21735613	6.01440792	6.03688537	6.108442	6.1734607	6.1303371	6.1086514	6.1373035	6.12034462	6.10619507	5.992476452	6.094636661	6.165607349	6.015841533
Bra003610	6.91713385	6.963460697	7.06608801	6.75155097	6.90164007	6.73915775	6.25289204	6.28361021	6.2461039	6.2521168	6.1195898	6.1709399	6.58990329	6.50588291	6.719144237	6.613409736	6.972250413	6.511276975
Bra003611	7.204122514	7.114918171	7.44027187	7.45388727	7.27283265	7.20162901	7.63404513	7.43828739	7.2010884	7.7765299	7.1537264	7.3109894	6.77576911	7.13729573	6.761457661	6.833857604	6.599089445	6.701113427
Bra003612	8.159886368	8.258016821	8.21140737	8.29578178	8.35967177	8.06312778	8.09359078	8.25125795	7.9761865	8.0671325	8.1087636	8.3080374	7.60555701	8.05430789	7.882869291	8.111946192	7.61797782	7.700757214

Bra003674	6.101000698	6.033622867	6.09836795	6.18594718	6.14129783	6.10679456	5.99067869	6.03332847	6.1721684	6.0852977	6.150951	6.1929094	6.03700758	5.99226836	6.06702226	5.990403548	6.200356039	5.98267401
Bra003695	6.571766929	6.581054635	6.4749043	6.4113081	6.31852984	6.33400664	5.89560109	6.15304685	6.0861063	5.9815017	5.8631848	5.9966531	6.14332375	6.149506	6.16657877	6.112576059	6.121707855	6.141053237
Bra003696	6.819896121	6.818762256	7.34798445	9.38631639	10.2175542	10.9892147	6.93815422	7.56855461	9.4186443	8.0324168	12.100824	10.993306	6.63047456	7.10146409	7.102297852	6.646327	7.218904316	7.517602579
Bra003990	6.243366514	6.057151419	6.0250222	6.1753969	6.08531549	6.02837934	5.98006685	6.06519522	6.0688627	6.0853722	6.053901	5.9914093	5.93896318	6.15922638	6.188630311	6.037689238	6.172257728	6.13671794
Bra004051	6.839513004	6.654358836	6.96607415	7.19008139	7.74036678	7.50219403	6.34665925	6.17573273	6.3988771	6.4101536	6.4794862	6.4271313	5.95900675	6.18295454	6.374824278	6.333138017	6.206929677	6.28083995
Bra004081	6.423169774	6.394842949	6.74086212	6.52122056	6.27958149	6.43383181	6.29187619	6.1829848	6.3224203	6.2930993	6.2356026	6.3848718	6.47979988	6.52779472	6.496063214	6.345473649	6.803250007	6.871845535
Bra004133	8.288581625	8.347444581	7.89070283	7.97582772	8.13201231	8.21687971	8.17662205	8.42929899	8.2292959	8.5285419	8.8360267	8.7643657	8.22708397	8.0401383	8.617227264	9.141472154	7.109617352	8.198837908
Bra004153	5.972253533	5.880650547	5.97402742	5.92680073	5.8190169	6.00057774	6.06634674	5.85168909	6.0790179	5.9102678	6.0442634	5.947191	5.94195034	5.91331863	5.858764427	6.013824289	5.901412499	6.121956257
Bra004157	6.175305534	6.197858646	6.1086892	6.17395022	6.042594	6.18449257	6.30707341	6.26220704	6.1480252	6.3061505	6.2172074	6.2021878	6.29859158	6.24702263	6.206611352	6.23604962	6.43925834	6.486128225
Bra004323	12.16791521	12.17426898	11.6580414	11.6243351	11.1341262	10.8946953	12.383146	11.9467059	11.600594	11.755587	11.045482	11.100293	11.6327228	12.0412611	11.65397439	11.32136248	11.39828781	11.52770708
Bra005072	6.086705901	6.046309252	6.18160214	6.13310231	6.12540745	6.09224853	6.07337585	6.0697004	5.9924546	6.0283454	5.9677017	5.8912939	6.22447162	6.16477635	6.055568531	5.936460143	6.147805054	6.026053617
Bra005073	6.539179781	6.423305142	6.37654795	6.43648001	6.70529026	6.55585203	6.22848171	6.15746344	6.1343429	6.2091466	6.0563274	6.1717068	6.40495185	6.2599521	6.45255054	6.469883198	6.250316117	6.392786878
Bra005449	10.12838121	9.921893048	9.39943531	9.28934162	9.86567852	9.51338903	10.1630221	9.91029734	10.113329	10.113312	10.002105	9.8403244	9.95996691	10.006549	9.789399673	9.736147694	9.525594999	9.641980979
Bra005450	6.167991416	6.230407204	6.12054661	6.07321506	6.20333938	6.04631889	6.2424674	6.20751685	6.1681566	6.2752919	5.9879021	6.1785235	6.20979265	6.18509532	6.265364543	6.198859288	6.726057829	5.947935869
Bra005681	6.040169774	6.112692497	6.24232712	6.00519325	6.02851907	5.87172656	6.05302806	5.92756542	6.0608503	5.9103512	6.0272746	6.0115458	5.91680686	5.9868397	5.898042139	6.028941004	6.030852431	5.975733026
Bra006334	5.91940176	5.739370006	5.87703448	5.7427736	5.68554303	5.76745237	6.08415595	5.86587953	5.7693326	5.9095606	5.964102	5.879644	5.89740929	5.87058463	5.944161541	6.109726316	5.972976025	6.029675776
Bra006335	8.489851447	8.306259749	8.08945114	7.64596729	9.1725461	8.31681098	8.04528529	7.74148754	7.6945581	7.4092053	7.120121	7.0551743	8.80362669	8.51116117	8.516734073	8.45864875	9.396271556	9.171916624
Bra006347	6.345888353	6.277314059	6.62551037	6.38466818	6.38849488	6.40200897	6.5633668	6.76053257	6.8373755	6.7482714	6.8056764	6.5576804	7.17079878	7.1434953	7.462007074	7.162267629	7.366701075	7.10933463
Bra006348	6.865944078	6.760602729	6.83858617	6.82554127	6.66496585	6.71560192	6.75506831	6.75288145	6.7740989	6.7259125	6.6958453	6.553409	7.01946261	7.16541332	7.086289806	6.871909111	7.495566603	7.593784968
Bra006447	6.766622883	6.710745831	6.74978212	6.51162614	6.76652805	6.51831258	6.93828768	6.97741896	6.8109157	6.9651002	6.9718193	6.7426755	7.20682546	6.90360759	6.893692997	6.714850955	7.526516477	7.454707733
Bra007140	6.35217212	6.259684287	6.15341769	6.17929401	6.22845242	6.15773089	6.42280026	6.42976792	6.269488	6.2739182	6.0683776	6.2600215	6.43628229	6.42460191	6.159926775	6.467603369	6.115929354	6.43997975
Bra007318	6.156879597	6.185312802	6.28190244	6.23776788	6.22944998	6.17683453	6.52185687	6.49679881	6.5702267	6.3674525	6.6284046	6.539351	6.36379594	6.2994509	6.176242722	6.229425523	6.254068336	6.228596525
Bra008817	6.100633635	5.999195611	5.97919562	5.95540266	5.97071782	5.97745728	6.09635327	6.0702963	6.0212767	6.0746007	5.9652988	6.0434778	5.87878552	6.01833635	5.949500335	6.031015743	6.055481965	6.057298946
Bra008847	6.103767022	5.97720374	6.12640658	6.00640175	6.1946512	6.14030494	6.19582687	6.18941086	6.2399993	6.3309419	6.2027728	6.230419	6.23859714	6.07361731	6.25219352	6.153324709	6.750384202	6.69682832
Bra008848	9.420029063	9.262794899	10.5673467	10.9904323	9.65828356	11.6203979	9.68221667	9.10866422	9.6899414	9.5526246	10.296541	10.201257	8.82814776	9.09175206	9.490373619	8.972217499	9.887727593	9.086586954
Bra008951	5.902426329	5.884686839	6.05567342	6.24124043	5.9577468	6.01986161	6.20879109	6.02247592	6.1561891	6.0788646	6.01533	6.303839	6.33521461	6.27945844	6.118790344	6.083906238	6.465239697	6.076302058
Bra008991	7.323698341	7.243909165	7.31491068	7.15894936	7.44584854	7.61659926	7.56207934	7.5489462	7.5426594	7.8785234	7.6875593	7.749897	6.91257745	6.88789803	7.230109368	7.004347564	7.334106491	7.24337824
Bra008994	6.134760273	6.059269617	6.21640562	6.0648109	6.24890223	6.04166431	6.29583998	6.36685809	6.4090801	6.3692569	6.3718612	6.3330627	6.28979159	6.03316963	6.335884885	6.223206478	6.245775958	6.054361465
Bra009060	7.60650749	7.607249835	7.49460602	7.33664725	7.87278066	7.3553419	7.51859669	7.6065096	7.5385781	7.8387615	7.5192851	7.7583954	7.5258374	7.79959426	7.831236612	7.881481196	7.429690646	7.657315699
Bra009061	6.595597462	6.730077592	6.32519321	6.72198394	6.26259258	6.51917352	6.44254807	6.29351096	6.3595134	6.3510579	6.3376549	6.2649766	6.77298781	6.50134095	6.401872691	6.34535925	6.554557498	6.423761838
Bra009062	7.374831137	7.1468662	9.50365128	9.57389203	7.98855884	8.97004257	7.42460776	7.05031437	8.7799308	8.3777413	10.062342	9.0594051	7.25548344	7.06141307	8.618710549	8.113112571	9.747868382	9.642806273
Bra009192	6.763122499	6.775981665	6.75928145	6.78745995	6.74844391	6.61561319	6.50716174	6.73944473	6.4597954	6.7084928	6.3341662	6.6367693	6.47335638	6.57011423	6.59389478	6.602248795	6.610625447	6.267797348
Bra009193	6.697059132	6.645936262	6.37614264	6.56658535	6.27893815	6.55150121	6.45444018	6.43026164	6.2378462	6.3871631	6.1845045	6.2843154	6.4713745	6.23526189	6.306112809	6.254488617	6.279680744	6.53952586
Bra009273	6.197330908	6.080922673	6.13010805	5.99007395	6.08178736	5.91669869	6.46750709	6.09569157	6.2086402	6.2222472	6.3161905	6.2174802	6.20613334	6.2565719	6.112067332	6.146262778	6.080778273	6.114693052
Bra009287	6.294443152	6.248138871	6.50911816	6.34410651	6.42468055	6.33879403	6.20023261	6.31637471	6.6632286	6.4247832	6.3810514	6.4804658	6.35077447	6.37801153	6.426927097	6.257735549	6.607337759	6.365553794
Bra009315	7.717228792	7.837713356	7.48903419	7.27530422	7.49324738	7.23685914	8.30272883	8.09201345	7.8478184	7.6030633	7.5169907	7.3195276	8.34816169	8.20162733	7.902289919	7.696522179	8.329926238	8.381251911
Bra009329	6.604076158	6.719366083	6.4974262	6.84649239	6.59446499	6.93006035	6.9232584	6.80042215	6.8047874	6.5969209	6.9000387	6.9625808	6.90512189	6.5474101	6.99759258	6.756727297	7.335125383	7.003978854
Bra009330	6.154114656	6.192412143	6.22745786	6.027881	6.22977516	6.17431809	5.97209162	6.17042443	6.0584373	6.2740434	6.1903462	6.1710855	6.09082358	5.92984029	6.080499216	5.996648828	6.43995559	6.599717116
Bra009332	6.115637371	6.199351802	6.11950112	6.13370938	6.19566474	6.23161129	6.02537152	6.12571192	6.1428013	6.2669676	6.1818555	6.1921371	6.09325468	6.10868314	6.190327874	6.038367367	6.334063314	6.179750306
Bra009348	7.994529012	8.216212691	7.34557828	8.19389316	7.23652055	7.85815234	8.35837615	8.00034733	8.3715516	7.6292179	8.1420575	7.9792061	8.43810529	8.20942711	8.359023942	8.067134459	8.623620063	8.126514235
Bra009349	8.301367417	8.508133337	8.25383851	8.02161131	7.92412056	7.48822596	8.39048685	8.55598458	8.0312086	7.9289204	7.7357059	7.7747579	8.97439474	8.69972124	8.352962281	7.976723493	8.913858107	8.644386354
Bra009351	8.620547447	8.855761232	7.76423345	8.64029292	7.66329978	8.71158094	8.83497196	8.29388898	8.8310797	8.3813585	8.7174027	8.4605464	8.86585773	8.58007649	8.839630418	8.565152286	9.051988238	8.616161421
Bra009442	6.161984311	6.137883199	6.26399445	6.22728411	6.23741876	6.12684456	6.04632057	5.99030775	5.9764135	6.0351135	5.9716887	5.9950399	6.06374465	5.98371041	6.1363111	6.001071889	5.97274745	5.992851991
Bra009443	8.20951791	8.290758453	8.55836594	8.35715337	8.38061848	8.69353512	8.20569048	8.46278884	8.1790549	8.3919587	8.0737425	8.1577303	8.61606459	8.40503195	8.680883597	8.802661741	7.90218548	8.176479409
Bra009445	5.928376917	5.950173627	6.08031772	6.06264901	6.12082964	5.80547563	5.8748469	5.82024625	5.9006075	5.8764191	5.7893094	5.6759068	5.89052502	5.80693493	5.899844294	5.786460183	6.080181809	5.965407218
Bra009511	6.290085919	6.240193893	6.14877651	6.12477197	6.40448894	6.18097923	6.22145614	6.20973878	6.2167767	6.0914378	7.3088754	6.199839	6.03520205	6.1574034	6.121287425	6.265934381	6.20558684	6.090891863
Bra009612	6.131015048	6.141494915	6.06898244	6.16009263	5.97085935	6.06069586	6.14379222	6.12308586	6.1344661	5.9810852	6.1029668	6.1992616	5.90366378	6.29375063	6.118987298	6.077948879	6.05580052	6.086508047
Bra009630	8.133989065	8.386176861	8.09969412	7.72180005	8.53739862	7.83436017	7.92103587	8.40446247	8.5630821	8.31346	8.2506719	8.1586923	8.71399756	8.42468341	8.569067187	8.527311243	8.16686743	8.099437036
Bra009633	7.609476285	7.607026081	7.58331964	7.39345391	7.35039041	7.18652753	7.01521946	7.17254896	7.1621419	7.0326459	6.765048	6.7012535	7.46034699	7.36481419	6.994073943	6.923010876	7.427222007	7.66768629
Bra009651	9.285798951	9.388544685	8.71735418	8.59893439	8.81790385	8.58006644	9.25300481	8.79915166	8.1655949	8.3867785	8.1179336	8.2407056	8.58775195	8.6045611	8.473332822	7.991035268	7.559776056	7.051193907
Bra009690	7.734424926	7.821687147	7.66595391	7.54219032	7.73512583	7.53674381	7.83097944	7.54658353	7.4688301	7.6545167	7.5703614	7.5593508	7.28706312	7.31189232	7.577734847	7.519855634	6.969507675	7.089910386
Bra009691	6.175339488	6.256184211	6.1592543	6.10524653	6.44473568	6.24575733	6.33639699	6.28687733	6.2729529	6.4557495	6.2229657	6.3613943	6.34514457	6.31652119	6.506729313	6.636777061	6.281587509	6.13101402
Bra009743	6.391156879	6.404497463	6.72638279	6.36820137	6.62631201	6.6677391	6.16634189	6.26578582	6.1636045	6.2109788	6.1323987	6.2833982	6.58888111	6.42580864	6.886450415	6.497491162	7.389558401	6.661282149
Bra009744	6.606968432	6.742775162	6.59234308	6.5685345	7.03309467	6.86393097	6.3470026	6.37915968	6.8537888	6.7394522	6.9046335	6.7765415	6.21655054	6.29693123	6.495193261	6.652087026	6.328668671	6.488848588
Bra009778	7.042329861	7.219912585	7.4316011	7.38771164	7.18179485	7.35816914	7.59900137	8.01375851	7.9101751	7.5840744	7.5020285	7.4445041	7.76505448	7.42592171	7.486116687	7.194423685	8.029919613	7.437575328
Bra009786	5.973718307	6.015025125	5.98418879	5.90720017	6.00441659	6.13922828	5.98682993	6.09157225	6.1014879	5.9702213	6.2348087	6.0999964	6.12619942	6.09793862	6.097206915	6.048459285	6.200505108	6.070316372
Bra009788	7.609288737	7.781143648	7.82027091	7.70649666	7.91715677	7.70655909	8.08372047	7.8734985	7.9942207	7.8166768	7.7544641	7.6847902	7.96290783	7.84229906	7.851017255	7.790369005	7.45196732	7.189726481
Bra009804	6.907893439	6.822906848	6.39800229	6.36340524	6.8801595	6.43860786	7.46641986	6.76459097	6.3280766	6.2677025	6.1887715	6.1516211	6.47927267	6.61656324	6.299154799	6.136502279	6.149528821	6.223954849

Bra009810	7.339836241	7.806127181	6.83259669	7.67369476	7.02896374	7.82835368	7.45249927	6.97714176	8.064407	7.1106298	7.7320927	7.6649816	7.96594078	7.96650858	8.719625858	8.408094806	8.50327459	7.810695488
Bra009814	7.78409604	7.845327522	7.10053986	7.59316958	7.36881722	7.74387008	7.80587337	7.835638	7.8243293	7.7168059	7.7107716	7.7948112	7.49306885	7.90959246	7.137088379	7.995336747	6.637815183	7.545706708
Bra009823	9.860305326	9.770312883	9.99503333	9.74563196	10.2771134	10.1100992	9.5210494	9.17312647	9.9767766	9.6287142	9.8805726	9.527395	9.07549912	8.91461839	9.471121268	9.329567211	9.159531712	9.005350368
Bra009941	10.07418689	10.19253164	10.1851138	9.95802528	10.0214265	9.6023296	10.0977906	10.0562196	9.9904892	10.027895	9.589779	9.5833438	9.82339405	9.76950683	9.791949873	9.599413536	9.162261218	8.712507613
Bra009973	6.206590225	6.051081263	6.23337859	6.18122397	6.18579271	6.03321829	6.13822994	6.1811086	6.1801424	6.1574769	6.1495957	5.9965978	6.17481203	6.2079544	6.270369035	6.213658569	6.126060193	6.292459056
Bra009993	9.161692737	9.614461556	8.75858379	8.4228262	8.92947683	8.51410299	8.74530906	8.83362756	8.2615195	8.5592415	7.8907349	8.2257958	9.34830736	9.11343415	9.289821288	9.199779447	8.886078552	8.611825468
Bra009996	6.829534968	6.970806292	7.05057825	7.03793768	6.91976711	6.80154793	7.91040298	6.56819356	7.4639648	7.2950855	7.9354827	7.7470153	6.59770299	6.82775818	6.372782293	6.544601979	6.390759551	6.525793199
Bra010000	6.098139454	6.090613583	6.31039588	6.25082	6.07606152	6.07344521	6.10819137	6.2047974	6.3330574	6.3217588	6.2791183	6.1678756	6.03471265	6.19413981	6.067884789	6.083331851	6.001769252	6.040278262
Bra010002	5.960984645	5.949928244	6.04421444	5.81602679	6.19262816	6.09901279	5.95809275	6.21652128	6.0017849	6.0218229	5.8915018	5.9505379	6.01994277	6.01092866	6.309226568	6.775583201	6.050027914	6.195730473
Bra010003	6.366735901	6.552692902	6.56384291	6.47604231	6.50066089	6.46120729	6.07800422	5.89433818	6.3073564	6.0688046	6.4017612	6.0180569	6.37924223	6.45492169	6.254042915	6.293485189	6.080007634	6.266295917
Bra010350	14.01117087	14.15991048	13.628561	13.9178144	13.1374946	13.1435996	14.4590694	13.4267409	12.665661	12.794865	12.23926	12.253522	13.2823126	13.789166	13.20835028	12.59613398	12.22761988	12.03351431
Bra010351	7.692443063	7.59753889	7.60814843	7.66302426	7.66704357	7.45672	8.37243247	7.64359079	7.8137927	8.0405787	7.8969876	7.7205496	7.01029072	7.32707158	7.541928467	7.529174712	6.840143862	6.796542547
Bra010713	7.287979703	7.083217249	7.09343398	6.6178997	7.76252643	6.97428472	6.80248179	6.82690862	7.4484873	7.4428795	7.5973167	7.267607	6.24424256	6.15648581	6.24933423	6.35466559	6.231948349	6.450112157
Bra010714	7.022587605	6.849600799	7.17125478	6.94364474	7.05258844	6.92887418	7.41040257	7.35426915	7.5227827	7.2559322	7.3444776	7.0701861	7.09745434	7.04159293	7.21453455	7.098613751	7.42007879	6.97147551
Bra010770	7.811906291	8.073659234	7.95864028	7.84967313	7.55314756	7.52974734	7.89971739	7.51492598	7.6030018	7.3165779	7.4439805	7.1405182	7.81424699	7.88246941	7.376782246	7.100488205	8.381677474	8.270920325
Bra010855	6.02716747	5.906769094	6.10119313	6.088723	6.09202979	6.04314591	6.14758388	6.08107946	6.0181914	6.1013131	6.1170667	6.1325586	6.05568775	5.98145044	6.098984106	6.008986448	6.038700055	6.243968348
Bra010856	6.94713555	6.612293464	6.61150947	6.53384072	6.64878898	6.63928799	6.85032162	6.75811623	6.7582256	6.7384149	6.7091509	6.5563328	6.72067276	7.06261296	6.614505162	6.95523322	6.625173264	6.956122843
Bra010958	8.508856839	8.354791254	8.46436872	8.3132705	7.79723915	8.02528347	7.29822784	7.48609878	7.2435778	7.2949237	7.4022697	7.1402526	8.88346071	8.56066966	7.877332404	7.704911345	8.612251699	8.063683178
Bra010959	9.033595338	9.135037326	8.93682704	8.74486212	9.01134091	8.68365475	9.12799119	9.32908472	9.0859448	8.9859032	8.8844427	8.9062789	9.47475634	9.21960033	9.316427594	9.437023393	8.848489927	8.512265288
Bra011041	6.291731531	6.201478721	6.18905584	6.26085825	6.34664233	6.27008183	6.18799639	6.26829181	6.326863	6.2964539	6.316611	6.2564692	6.19229827	6.34735675	6.4333511	6.247052143	6.610332317	6.591601183
Bra011064	7.810073134	8.06635066	7.56920823	7.5800427	7.88889057	7.53534664	7.15220348	7.36241766	7.2851781	7.4226561	7.3089459	7.4101261	7.56735456	7.68167406	7.700962183	7.690020701	7.555855725	7.402368913
Bra011066	6.654965417	6.617761616	6.86827257	6.48087703	6.59949468	6.49146744	6.33238174	6.38193555	6.265752	6.1532625	6.375632	6.2469081	6.56318278	6.77465649	6.696177665	6.462696589	7.412483224	6.979705248
Bra011135	6.025029149	6.024629375	7.08303362	6.6639783	6.0273897	7.46709712	6.0153577	6.23865894	6.0466457	6.0982591	6.8280911	7.5303443	5.99667759	6.01127561	6.195530868	5.992784922	6.284673269	6.396959069
Bra011270	7.465049215	7.638039115	7.59862731	7.74450962	7.83155848	7.94572694	7.62572989	7.48978622	7.6866774	7.9371418	7.7728189	7.9444036	7.27996156	7.55300182	7.670028445	7.864317515	6.916776557	7.25171342
Bra011272	6.403994019	6.451988856	6.56443493	6.39349187	6.43314347	6.24512515	6.25848865	6.25481138	6.0976546	6.2385167	5.8585786	6.1910918	6.15455608	6.42276595	6.33368938	6.198348796	6.211481458	6.271306376
Bra011371	7.136386056	7.216618405	6.94140095	7.10555065	7.1489044	7.23516076	7.50770422	7.4265237	7.3665609	7.6372901	7.4046205	7.703474	7.17134069	7.45274113	7.31802581	7.459346075	6.997623112	7.304835086
Bra011372	5.909453065	5.953213356	5.94558288	5.94758812	5.93669801	5.92716696	5.92602321	6.19782434	6.1504421	6.0508235	6.1479469	6.116127	6.37740699	6.40691482	6.167245791	6.344603396	6.089539484	6.714478803
Bra011380	6.019981611	6.067509666	6.2743179	6.16837455	6.24895027	6.11265422	6.18637368	6.18891928	6.1583237	6.26966	6.1147817	6.1145766	6.16242297	5.97301805	6.049332312	6.115953658	6.112273261	6.088146456
Bra011382	7.656205687	7.628524405	8.60302503	8.17437088	8.58694373	8.5067423	8.19009425	8.51858863	8.9145647	8.8551953	9.1191874	9.1837057	8.66626115	8.45732356	9.317602197	9.504567569	9.29004007	9.89488122
Bra011455	6.118631966	6.163117815	6.18769756	6.21154294	6.12181883	6.09161449	6.23678335	6.31919384	6.2075772	6.3226029	6.2975856	6.2855601	6.09048956	6.14336379	6.301083628	6.372861325	6.069870646	6.345338511
Bra011638	5.899357715	5.846693128	5.97558223	5.89679495	6.06863467	5.8704521	6.02386729	6.02681662	5.8970987	5.9033526	5.9525271	6.0100058	5.93338661	5.91215109	5.98286299	5.952597289	6.034326792	5.886989072
Bra011671	9.119901804	9.721754675	7.43648201	7.47231488	7.96024406	7.75614776	8.48998555	8.16882134	7.1022879	7.2422142	7.5427558	7.3744284	8.80047647	8.79877769	7.87309482	8.18757258	6.79733382	7.18811602
Bra011672	12.45889024	12.06593164	11.7030363	11.3759188	11.351537	11.2064355	11.2930234	11.2476542	11.436395	11.066183	10.992944	10.69161	12.6215616	12.3201626	11.29659225	11.59639325	12.29630028	12.41710896
Bra011936	6.644940871	6.83667547	6.4618389	6.21448631	6.43328818	6.38502412	6.70676867	6.5323707	6.4923264	6.3512387	6.2083181	6.4527354	6.34501819	6.44985975	6.361861995	6.373173339	6.278580993	6.39419072
Bra012062	7.113103829	7.141497957	6.50881156	6.78672704	6.47289638	6.61098695	6.71500034	6.62847338	6.7014955	6.4934494	6.3707832	6.5305959	7.06790745	6.81598066	6.723719267	6.750310172	7.269152887	6.775355864
Bra012144	6.175019045	6.170488372	6.08174991	5.98198008	6.19193813	6.06884153	6.51533725	6.3595338	6.2370158	6.3558924	6.4052625	6.4397893	6.17561058	6.45478665	6.212714716	6.436592775	6.121876129	6.341208024
Bra012145	6.601940197	6.52344899	7.05147561	6.90225006	7.36031463	7.0916562	7.0819915	6.95040948	7.5626505	7.7234929	7.5412818	7.7118231	6.91416512	6.94102715	8.016372548	7.883228758	7.242301618	7.103473497
Bra012336	5.958848539	6.022161825	5.93725272	6.01701847	6.04224105	6.04313425	5.99671685	6.08001459	5.9538105	5.9590879	5.9847758	6.0656059	6.08127858	6.15296615	5.945733876	5.912731068	6.045681194	6.196036408
Bra012337	7.020849409	6.962316862	6.64878055	6.97527205	7.07658058	7.12861397	7.4895706	7.35095929	7.7369774	7.7392957	7.9136289	7.4421118	6.90057197	7.15832236	6.584107263	6.788203821	6.532196112	7.084228404
Bra012352	7.90299916	7.799141248	8.79315346	8.59912795	8.15316031	8.09458888	7.38398482	7.45771449	7.2486654	7.1441795	7.5481058	7.2506923	8.09819076	7.89681525	8.470225196	7.789475135	9.470511283	8.763663551
Bra012354	6.116404913	6.10901558	6.09462385	6.06248487	6.01037639	6.02790652	6.08600768	6.26908118	6.1906209	6.1882948	6.2330831	6.0151078	6.11385944	6.18702036	6.161148426	6.048732091	6.061783779	6.123277037
Bra012393	6.375837102	6.251381758	6.58336956	6.47886414	6.74935345	6.51807533	6.47440934	6.4113162	6.5071375	6.6851406	6.6359146	6.7140493	6.30312181	6.45216597	6.530547494	6.830762702	6.236742252	6.528621604
Bra012396	5.880478039	6.009222536	5.86311831	5.98310182	5.83031359	5.97760048	6.17719881	6.13036637	6.0296017	5.9875178	6.1551038	6.1257997	5.96747702	6.01513322	6.117959816	5.992706887	6.139968528	6.026247086
Bra012417	7.000046439	6.973845411	7.00270624	7.10766093	7.01921372	6.97704334	7.31356034	7.0122838	7.0836695	7.1235084	6.8541812	6.9255527	6.94005636	6.92760382	6.908374065	6.837687323	7.298912925	7.353795381
Bra012465	6.349525178	6.211621657	6.50917039	6.21576599	6.21319799	6.241296	6.18687369	6.27975897	6.1755385	6.0806864	6.1974198	6.2868022	6.22141645	6.2294469	6.332151849	6.241710531	6.500769713	6.368688913
Bra012959	7.881653346	7.574148658	8.37811721	8.22777822	8.92080438	9.05927874	8.07508923	9.06917308	11.068834	11.15269	11.075623	10.199236	8.80291012	8.79112563	9.157413832	10.10250628	9.447053417	10.75017729
Bra012960	7.022573399	7.097310355	6.49023626	6.82388112	6.39506944	6.57612544	6.50591354	6.6096633	6.3757566	6.6348016	6.2711316	6.2955747	6.50194339	6.73978584	6.411935788	6.673678403	6.508339338	6.548069035
Bra013072	6.348461815	6.22315077	6.40106034	6.45884195	6.46829155	6.42193464	6.58047901	6.68016094	6.8887911	7.1011138	7.2082217	7.0057896	6.38730835	6.58143886	6.74261433	6.716319431	6.767966214	6.513276501
Bra013073	5.9777731	5.968933976	5.91857372	5.98751052	5.93797299	5.98885822	5.76426824	5.82756678	5.9225085	5.9897055	5.9112797	6.0990052	5.88297768	5.92878224	5.982830463	5.930378587	6.011088395	5.897398996
Bra013447	5.836175294	5.837249795	5.8382131	5.75185329	5.78869806	5.74145148	5.82871062	5.97932054	5.8750368	5.7917169	5.8998179	5.8329545	5.78988296	6.00825731	5.887178858	5.865732844	5.953491409	5.879296867
Bra013448	6.393640812	6.403858526	6.50220442	6.46838908	6.47858863	6.2835899	6.52080494	6.42768406	6.3024492	6.3024341	6.2430646	6.4024572	6.32088057	6.4085669	6.435449245	6.275674723	6.552599327	6.338132612
Bra013455	6.206465553	6.329135259	6.24126837	6.19395765	6.20831449	6.15688075	6.20137629	6.16198127	6.1693794	6.2291212	6.2798648	6.1581924	6.26186223	6.21501055	5.931731785	6.191721731	6.246858122	6.287848055
Bra013456	7.044693769	7.167823579	7.0776627	7.10230144	7.00775725	7.25971836	6.98082241	7.40416009	7.594858	7.9344836	8.135288	7.7611922	7.01595868	6.98019817	7.239217357	7.198279289	7.484942888	7.341717813
Bra013550	6.829765772	6.677930386	6.98082643	6.97027067	6.83289562	6.64725563	7.14177072	7.08105715	7.3107253	7.0498648	7.1374784	6.8601659	7.39410834	7.17679392	7.01374315	6.937250219	7.79809724	8.308053547
Bra013651	5.79659394	5.9573187	5.8550522	5.87925858	5.89861337	5.95585252	5.8354686	6.00046282	5.97025	5.9980335	6.1405321	6.0208921	5.90178049	5.92281394	6.03454868	6.005158024	5.940975469	5.821832592
Bra013788	6.395040498	6.696343582	6.54986886	6.50587414	6.33158019	6.37317479	6.52534504	6.5520531	6.4798324	6.3167997	6.3869827	6.2376158	6.43831433	6.62517372	6.46292408	6.346776196	6.51522644	6.439761991
Bra013927	7.50177925	7.396876242	7.26398729	7.21400557	6.96843151	7.36680972	7.59119259	7.27208059	7.553487	7.1056863	7.3506162	7.0077361	7.84576351	7.55302812	7.104021469	7.160967471	7.933520979	9.760690646
Bra013928	6.62304872	6.524611714	6.65736903	6.58615484	6.64912174	6.3861774	6.58898431	6.96514845	6.6191994	6.7879701	6.6728705	6.8918892	6.63084152	6.62025183	6.436719875	6.513163417	6.596393111	6.808362974

Bra013931	7.601261202	7.297885639	7.80261996	8.29865167	8.48410666	9.74525034	8.20566806	8.10071812	8.9169756	8.9887452	9.6738188	9.7219148	7.3539025	7.44044951	8.509495468	8.310154653	9.37585306	9.009767629
Bra014000	12.74967191	12.64539633	12.5578355	12.309136	12.8965562	12.5094299	11.5701824	11.7007927	12.124908	12.320362	12.175162	12.502372	12.3530479	12.5124807	12.5297734	12.59365676	12.07946649	11.9477231
Bra014001	7.373890819	7.570826769	7.29369656	7.48161336	7.63333259	7.51615689	7.86957907	7.56194523	7.9216034	8.085403	8.0522242	8.2905383	7.63475252	7.82321246	8.316371443	8.512513238	7.520386918	7.424909105
Bra014006	6.053832072	5.886178267	6.02916697	5.84918323	6.12590579	5.97591411	6.02914636	6.12030442	6.00105	6.1088423	5.9403372	6.0533498	5.97142952	5.96807631	5.991666916	5.854134134	6.03319339	5.894686916
Bra014010	6.398450887	6.418362421	6.15258254	6.17844071	6.12614278	6.02476344	6.42394912	6.25782205	6.1444976	6.0998778	6.1142117	6.0995898	6.29356868	6.40038215	6.335664854	6.220956706	6.563273919	6.411240217
Bra014011	10.269573	10.26262111	9.94881061	9.94211807	9.92702943	9.75813885	10.3054509	10.2338907	10.155091	10.2055	9.951625	10.10739	10.1485312	10.28845	10.02908917	10.04869046	9.618808291	9.74638425
Bra014024	11.4815887	11.31929566	10.779733	10.7625871	9.86554606	9.73862645	11.5864664	11.1773645	10.248775	10.35847	9.9602796	9.7945054	11.4598172	11.5672242	10.50983536	10.08543072	10.05044062	10.5287217
Bra014025	6.090956848	6.040799271	5.94509889	5.94562594	6.1624635	5.93281655	6.03733862	6.0915257	6.0840454	6.1663319	6.058282	6.0828935	6.01599054	6.01917552	6.053026518	5.896777674	6.165615479	5.971035074
Bra014028	7.204780766	7.244884878	6.86215994	6.99134195	7.43569214	7.03774759	6.95247922	7.19549821	7.0978296	7.0830901	7.0736484	7.2909296	7.15281068	7.18715105	7.124822862	7.439253473	6.717569468	6.75590379
Bra014029	8.998176176	8.999263607	9.22133496	8.83592243	9.93642323	9.02623539	8.33003502	9.31054239	10.383812	10.255448	10.818188	10.175413	10.0776023	9.92678448	10.68895612	10.55559563	10.52186291	10.30074631
Bra014030	6.160277709	6.127591954	6.08027508	6.25630651	6.07905055	6.21563032	5.95239635	6.18974099	6.0347101	6.0096991	6.1245932	5.9384875	6.28793056	6.01088083	5.998366125	6.053080738	6.306310403	6.159168539
Bra014031	5.816770095	5.92887446	6.00714307	5.79358009	5.89250004	5.9172256	5.96096855	5.97925082	6.0554121	6.0485074	5.9215556	5.9296499	5.91491025	5.96038961	5.96221477	5.909399825	6.015222897	6.003735467
Bra014035	5.980356691	6.189654763	6.04057366	6.10156205	5.96660018	6.08772433	5.83142709	5.95403531	5.8673808	5.9081829	5.8581172	5.80751	5.99900817	6.1245405	6.028245022	5.993263919	6.048532203	6.078204738
Bra014138	6.22100378	6.100429187	6.25346284	6.14713474	6.26161029	6.21301239	6.01904741	6.11738836	5.8907058	6.0375634	6.0076895	5.9780575	6.24197325	5.9751441	6.398830665	6.126584286	6.186068857	6.130321003
Bra014158	7.654191932	7.573065151	7.57087801	7.17037098	7.50180097	7.18512643	7.3106256	7.57074736	7.3399352	7.2791316	7.2605923	7.2512315	8.46275196	8.21367089	7.702014625	7.559844989	8.607463623	8.052375548
Bra014159	6.193417418	6.084701688	6.16451729	6.1731333	6.2485675	5.9692977	5.94713189	6.0676496	5.8945467	5.8060111	5.8555259	5.9419139	6.51712348	6.13367781	6.186921506	6.124780048	6.467802069	6.074703726
Bra014179	10.45187827	10.48296739	10.519595	10.8914257	10.8302289	11.1151159	10.5880268	9.91703675	10.659261	10.908374	10.844978	10.606566	10.321649	10.3789689	11.34547431	11.25585512	11.32718038	11.28121623
Bra014180	5.805701975	5.934763292	5.75552245	5.9265235	5.87290696	6.00849753	6.63317144	6.41929277	7.1329015	7.2310208	7.6895792	7.3967147	5.76743161	5.86318215	5.898838557	5.880202791	5.835574467	5.903512678
Bra014244	6.774760392	6.825718115	6.59575055	6.69175074	6.58720657	6.71929358	6.91536428	6.86911823	6.7621735	6.9116307	6.7609863	6.7578615	6.74838738	6.61925463	6.527363354	6.499367151	6.60366134	6.481852832
Bra014245	9.569312357	9.661678668	9.46345408	9.25072313	9.28696193	8.992507	10.0648462	10.1947892	9.6534586	9.618733	9.3447617	9.191957	10.248614	9.8368148	9.588706456	9.596455198	9.79670052	9.894973014
Bra014299	6.350887215	6.252919194	6.38337205	6.42091919	6.2775958	6.28910877	6.53589546	6.53245624	6.5335593	6.4353288	6.6126834	6.4104005	7.03690867	6.62056699	6.804076558	6.474159245	6.979795318	6.892474664
Bra014300	7.465388647	7.407022296	7.45736248	7.49712559	7.88407216	7.54260272	7.39170906	7.37881947	7.698885	7.9013706	7.7452865	8.1044523	7.10009841	7.30853425	7.496061203	7.885319849	6.759551633	6.979343347
Bra014308	6.60254259	6.606245016	6.41021285	6.83273644	6.18406238	6.23250459	7.45151832	6.99210732	5.9564786	6.1460239	6.0596688	6.1577386	6.50250279	6.53007975	5.994945108	6.122421248	6.133938574	5.93688058
Bra014309	6.321224902	6.246431067	6.22956103	6.38420184	6.18032558	6.20940476	6.28980914	6.28999402	6.1612215	6.2207898	6.250364	6.2353003	6.11984681	6.23086567	6.117841	6.102910508	6.380144191	6.381687769
Bra014719	6.830407844	6.935393556	6.72504105	6.79174615	7.11533618	6.96233899	7.05786704	6.86299082	6.7360373	6.9918347	6.9646945	7.1884588	6.93251172	7.08241418	7.271613992	7.286433161	6.679442807	6.747699388
Bra014879	6.629928442	6.538889715	6.3640125	6.54871317	6.39902411	6.62781154	5.96070364	6.23296897	6.1380058	6.2272876	5.8954329	6.0196357	6.07697051	6.09540394	6.216756366	6.171822253	6.108645965	6.09425306
Bra014889	6.665102545	6.844011859	7.11324415	7.05641383	6.86349486	6.78685193	6.02754581	6.06190048	5.8702801	6.2024305	5.9221947	5.9484765	6.5311945	6.29822349	6.590455805	6.333690109	7.31426466	6.664394655
Bra014890	6.500289342	6.361608959	6.7550389	6.43568603	6.43395756	6.63343012	6.41518265	6.62424036	6.8544667	6.6763014	6.8318225	7.29415	6.39132695	6.58496187	6.628722015	6.676022998	6.887011228	7.146884241
Bra014891	8.322697506	8.592984996	7.64830432	8.01464215	8.14878811	8.68065515	7.53094685	7.75593164	7.6682414	7.598803	8.1511011	7.8444603	7.67958005	7.82417058	7.55827691	7.71997899	7.649662774	7.900387331
Bra014955	7.526299315	7.182119652	10.4255489	10.0241854	8.99423721	11.1148589	7.95883481	7.67067368	9.2515771	9.0203633	10.58441	10.276438	8.13975623	7.83284688	9.703841685	10.33087514	10.62245918	10.98783926
Bra014969	6.30763588	6.262992288	6.31951487	6.27549941	6.27852271	6.25271658	6.50511083	6.87570761	6.8720731	6.4829332	6.5531588	6.4381449	6.91703136	6.74048573	6.557006289	6.496899858	6.879410312	6.753876642
Bra014971	6.013390137	6.167955181	6.07138985	6.19778583	6.05599752	6.00657552	6.11696769	6.23755078	6.0035866	6.07638	6.1353538	6.0933409	6.15961576	5.93742766	6.161297817	6.003286168	6.127640689	6.047036914
Bra014972	6.344689855	6.269358159	6.32628362	6.46224317	6.5976201	6.49643008	6.26664189	6.58142177	6.408308	6.6614389	6.713131	6.7323509	6.34682479	6.35630716	6.168202731	6.454555708	6.295266021	6.462243159
Bra015001	6.602038703	6.629983782	6.88813784	6.79584629	6.497621	6.46507341	6.8264032	6.80141053	6.5781449	6.6841353	6.6500841	6.629646	6.65947043	6.79097915	6.784846009	6.786152549	7.124351728	6.746739642
Bra015002	5.903927318	5.965567399	6.0801232	6.10084569	6.00572619	5.99096006	5.94026403	5.96023294	5.9683366	5.9544077	5.9809905	5.880982	6.0400149	5.9659838	6.063031776	5.933029805	6.121697846	5.848067909
Bra015052	6.34607907	6.328342886	6.53807584	6.40115525	6.43365452	6.72305478	6.61773657	6.48253622	6.6245651	6.5238809	6.6555979	6.9271046	6.46952224	6.91753821	6.43164251	6.689738531	6.372703808	7.291549169
Bra015053	8.697403109	8.567684861	8.9650323	8.21409729	8.27634598	7.78695087	7.80667979	8.11516501	7.5394177	7.546687	7.6135544	7.5649942	8.36747867	8.48975864	8.507258677	8.082551045	9.959243585	9.822761692
Bra015079	6.97091974	6.651269696	7.05712728	6.73675285	7.14241777	6.92586472	6.85358722	6.91536918	6.910979	7.0815526	6.9747111	7.0060765	6.81217749	7.00255166	7.019156481	6.954802757	6.504543598	6.677070754
Bra015080	6.577437975	6.528589177	6.40735283	6.58376049	6.33142181	6.6167197	6.15725269	6.21897256	6.2880274	6.2623686	6.196672	6.0977883	6.13040999	6.29949814	6.185188279	6.216236019	6.276981018	6.815841093
Bra015089	6.872245892	6.917699957	7.08844194	7.0694388	7.31746729	7.30033874	7.02119483	7.03414256	7.1920266	7.086218	7.6379768	7.4005761	6.72778822	6.82847299	7.318150993	7.354446697	7.011509077	6.755286786
Bra015090	7.204340768	7.23559673	7.59958704	7.31164743	7.43072098	7.28786111	7.72196072	6.82907421	7.3595749	7.0780347	7.2940046	7.0474871	7.75492223	7.60418126	7.409821929	6.980821093	8.09919181	8.288055385
Bra015095	5.954377085	5.964938021	6.05061757	5.98898377	6.16550158	5.93495898	5.98530425	5.93051765	5.8934963	5.9886884	5.9174511	5.9746386	5.9387303	5.95280948	5.853188421	5.87432054	6.112526278	6.077248335
Bra015141	7.256380026	7.14695166	6.94057752	7.10889879	6.86188109	6.79858479	7.29747697	7.57167071	7.6022576	7.3136622	6.9591431	6.8821395	7.53731495	7.41311135	7.054909546	7.20951775	6.998486499	7.213654262
Bra015522	6.157680299	6.15215599	6.25842895	6.17350376	6.30098314	6.08043681	6.08605312	6.05670351	6.2034481	6.0039969	6.0965321	6.1007836	6.31779146	6.10478497	6.509981273	6.086361157	6.397444395	6.132167828
Bra015533	6.101879301	6.165270704	6.13724462	5.943055	6.16326703	6.02537105	6.03019538	6.1202382	6.0186645	6.0747303	6.0491085	6.1473336	6.04138184	6.01893813	6.048759296	6.153255951	5.98780366	6.125854536
Bra015545	6.924094479	6.789870741	7.01194578	6.84980316	6.87154885	6.57322032	6.949308	6.88787526	6.7047039	6.7834806	6.7588136	6.8346348	7.18093446	7.08750953	7.006392926	6.825731612	7.838683315	7.775747596
Bra015546	6.356343358	6.461996538	6.59571869	6.80264231	6.68563757	6.70626035	6.42254733	6.51315812	6.5546654	6.8540299	6.6962352	6.8265266	6.80264434	6.57736482	6.776598134	7.081609458	6.562570711	6.614369526
Bra015550	9.530717504	9.567228987	8.60012229	8.66843435	9.04058471	8.8680549	9.45017115	9.0990542	8.2255015	8.3620432	8.8623125	8.3683047	9.26960149	9.33507948	8.841092059	8.86817883	8.50551289	8.122277778
Bra015551	7.292876642	7.008208928	7.10687813	7.61291992	8.64858191	8.70248612	7.54369863	7.49231086	8.9568886	8.9307857	9.6378239	8.9022114	6.93404642	7.21307398	7.478785167	8.710811072	6.442056676	9.138507654
Bra015552	6.150362919	6.317967322	6.16471581	6.2343574	6.18552986	6.09738958	6.35361823	6.07981875	6.3217708	6.2537982	6.2328418	6.2105054	6.07422646	6.13219045	6.13674583	6.174131598	5.969070164	6.127961016
Bra015577	6.366805784	6.52065436	6.29733758	6.50861393	6.40946127	6.43250707	6.15044841	6.46486727	6.2828378	6.4439681	6.3157232	6.2096231	6.36221118	6.3097682	6.070061789	6.183490227	6.300104312	6.33800247
Bra015586	6.611936249	6.282979741	6.70841297	6.73446483	6.55524128	6.57612224	6.20719545	6.35606449	6.2310985	6.2191481	6.2943467	6.2297778	6.27135158	6.54923937	6.301267831	6.850993389	6.100121534	6.455781832
Bra015587	6.023465897	6.136577603	6.1629571	6.17357059	6.18669898	6.06189573	6.07943606	6.13917586	6.0731858	6.1238311	6.1129359	6.3360371	6.14642375	6.01476768	6.047450697	5.982488582	6.143419352	6.067326274
Bra015751	6.774748427	6.628938987	7.28786423	7.33916783	6.57529395	7.79156955	6.82226398	6.82202449	6.6890867	6.8320367	6.9116929	7.1581229	6.82674804	6.59939521	6.746504002	6.568597111	7.001082884	7.03919875
Bra015752	10.54025794	10.52473961	10.2515685	10.154201	9.91300497	9.57819404	9.9562797	10.2993392	9.681141	10.00069	9.5367154	9.8544233	9.48021412	9.88392757	9.543232545	9.578859944	8.682039049	8.551190661
Bra016063	5.964459476	6.017487604	5.94900887	5.91611791	5.90780054	5.91815781	6.05175945	6.19204999	5.9772564	5.848626	5.9759972	6.0178502	6.21241546	6.01829142	6.032205469	5.993923757	5.897612932	5.805878132
Bra016064	6.241429058	6.229669383	6.07288816	6.266572	6.21186275	6.1297801	6.72479248	6.80712343	6.1923785	6.3240842	6.1332833	6.3540592	6.09540696	6.32423525	6.416043378	6.460262511	6.218484383	6.209111059
Bra016095	7.967259287	7.846287709	8.6412343	8.64196516	9.38098277	9.22406145	8.14417255	8.0583531	8.681049	8.6286468	8.9580402	8.4896715	8.17068719	7.88815628	9.265357219	8.913230194	9.46615685	8.872376465

Bra016326	6.393826838	6.301991601	6.44103133	6.33129521	6.43151642	6.35993369	6.37908023	6.39960989	6.3913403	6.373783	6.3805742	6.1448804	6.37939135	6.38150845	6.349423803	6.241584215	6.606342	6.395340291
Bra016327	6.015680049	5.932618179	5.92985997	5.91856433	5.98682076	5.92728444	6.24332475	6.14847693	6.2815105	6.1782043	6.1217691	6.1686336	6.23970505	6.1518681	6.004872694	6.223636827	6.301022142	6.2177914
Bra016439	6.782056176	6.730979036	6.67539369	6.86918809	7.08139362	6.90354484	6.8561383	6.50223842	6.8914045	6.8206457	7.0318513	6.8279466	6.64143053	6.62005004	6.894917234	7.076168084	6.829313636	6.727999626
Bra016508	8.162231231	8.222240356	8.64506326	8.65571884	9.29225725	8.87527716	8.49532796	8.44153474	9.0144665	9.1859929	9.3332814	9.3110383	7.98673214	8.29195648	8.934510087	8.812379777	8.528744131	8.7151619
Bra016509	6.38959644	6.15878211	6.50456877	6.75355315	7.20326563	6.83684329	6.25768626	6.26916835	6.5148909	6.6813786	6.5334415	6.6698595	6.15538337	6.1379648	6.350052481	6.536956811	6.328448776	6.076054449
Bra016600	5.874805932	6.024467981	6.00434081	5.95609963	6.04666188	5.95722973	6.40677402	5.98466279	6.1067995	6.1306532	5.9762556	6.1414901	6.80740448	6.94404672	6.871862976	6.980079673	6.512489191	6.392291393
Bra016601	6.940821838	6.922927112	7.08127057	7.51348383	7.55379354	8.01644059	6.54984124	6.65197147	7.6726073	7.9416331	8.1224123	7.8547736	6.23255198	6.21613183	6.155580734	6.542494973	6.065706411	6.27511363
Bra016602	5.987886038	6.033514727	7.33206393	6.9003545	5.92845194	7.39676654	5.99027498	6.18898492	5.8139446	5.796617	6.417023	7.8681634	5.88064022	6.00464296	6.008758744	5.882528454	5.998842745	5.944191743
Bra016603	7.912341066	7.741578226	8.39881722	8.18802609	8.11065782	8.52907306	7.74318468	7.65888887	7.9931055	7.8948509	8.0812879	8.1388808	7.99441589	7.85678516	8.142305617	8.149764974	8.063464925	7.803153338
Bra016865	7.310369596	7.228644123	7.96483112	7.68708421	7.76283085	7.95697898	7.02352387	7.15486747	7.779346	7.638729	8.1527272	7.8418028	7.4118096	7.41235728	8.94734693	8.931563331	8.169974047	8.547148815
Bra016866	6.547995506	6.401123304	6.37339522	6.27190404	6.1870368	6.18778625	6.57579931	6.44960058	6.4276437	6.5194787	6.2448444	6.3944597	6.6345579	6.49022622	6.268592729	6.354926623	6.304616871	6.284359304
Bra016936	9.280649708	9.105195226	11.2798746	11.575638	10.8263097	12.269531	9.07033541	9.45548078	10.544467	10.452621	10.543591	11.180149	8.72918922	8.95115765	10.61283962	10.5495983	10.28476127	9.647061594
Bra016937	7.140639099	6.922303488	10.4876662	10.6434534	10.0768271	11.8155297	7.75552942	8.47039034	9.9554439	8.411659	10.757362	10.651271	7.34534467	7.0526722	7.716188772	7.741848858	8.369636834	8.091115837
Bra016947	7.437097467	7.294774111	7.40433956	7.2494623	7.42258328	7.12233049	7.1281879	6.82377508	7.255793	7.2866276	7.7778395	7.9963405	7.27686805	7.23138526	8.633961822	8.473041694	8.320158775	8.261061584
Bra016948	6.20569831	6.181489343	6.23511481	6.14985203	6.42208754	6.27534793	6.10888294	6.13918098	6.2826275	6.0778739	6.2417088	6.2429218	6.03933846	6.29615435	6.487667273	6.324418924	6.987804187	6.674503437
Bra016950	6.441046247	6.550904899	6.30856135	6.60113552	6.59109643	6.68449182	6.7518846	6.64711204	7.0176373	6.9078063	6.8939416	6.9340331	6.37160519	6.54369384	6.426471036	6.467478226	6.469546122	6.63663017
Bra016955	8.476103376	8.381372848	6.9448865	7.03660229	7.11796428	7.00635868	8.59528525	7.64465561	7.0779111	7.1932751	6.8478717	6.9007984	7.41237571	7.69647104	6.748717022	6.830331684	6.26518122	6.616566265
Bra016956	7.216352872	7.188515994	8.47001318	8.21104263	6.55645825	8.59439808	7.0561446	7.07340637	6.8946953	7.0013807	8.3078984	8.5006654	6.69675917	6.59625841	6.692929841	6.595351119	6.96341635	6.731310553
Bra017274	6.585900258	6.490649204	6.47396356	6.23203627	6.35236441	6.29432095	6.03840902	6.06305663	6.0337244	6.1926669	6.1578677	6.190145	6.2892378	6.13110543	6.199685691	6.059744599	6.28616321	6.141075525
Bra017328	7.721761278	8.000888407	7.30512645	7.42729417	7.74703322	7.30736457	8.00007054	8.27566041	8.0466734	8.203896	8.0361219	8.1969317	7.51577965	7.772487	7.578578316	7.850503891	7.236455783	7.284064882
Bra017517	6.000288281	5.912873744	5.92805381	5.93500579	5.95194899	5.96632628	6.11230255	6.08897071	6.0737025	6.0546791	5.976757	5.97426	6.07128769	6.15086369	6.148104555	5.981618347	6.079047685	5.95243436
Bra017518	5.978845575	5.963845649	5.81049934	6.12826295	6.02728269	5.9412456	6.09287911	5.90589546	5.9137283	5.9224457	6.0255153	5.9862896	5.96626918	5.98207585	6.060718175	5.930359988	6.051442171	5.925930902
Bra017539	5.833099098	5.911231447	5.95869357	5.88985061	5.94532925	5.94116007	6.02205043	5.9424071	6.0839273	6.0036489	6.1391788	6.0001403	5.97040783	6.01441437	5.929146544	6.042474576	5.819273722	5.866977497
Bra017923	6.274564402	6.30784107	6.12585915	6.29256514	6.18811685	6.45957869	6.20013146	6.2212098	6.4932895	6.313049	6.873889	6.3863846	6.21213526	6.25899008	6.246693009	6.248974615	6.34211412	6.334506446
Bra017924	6.101107528	6.058554334	6.04540608	6.04306391	6.25625774	6.06486701	6.20020815	6.29098775	6.1879871	6.3096086	6.2677533	6.2502959	6.35318263	6.40538506	6.202838274	6.214541822	6.523855365	6.387831884
Bra017940	7.240678407	7.078294366	6.94601065	7.21907744	7.76227446	7.47018649	6.90367687	6.56367563	7.2665901	8.0470919	7.0651715	7.5959404	5.9444013	6.21640854	6.242230759	6.198506592	5.902834671	6.004988088
Bra017945	6.076438657	6.053038284	6.00797761	6.09015652	6.05289578	6.11157934	5.9990346	6.05514831	5.9927081	5.8441358	5.9824399	6.0443614	5.91328371	6.04701508	6.034036624	5.97303372	5.845841212	5.928216738
Bra017946	6.584281469	6.363413025	6.51032671	6.31053353	6.53740632	6.2872952	6.69766165	6.55049579	6.6321969	6.5635553	6.4898307	6.427984	6.75903397	6.59626666	6.869530213	6.694809656	6.82020462	6.418797046
Bra018007	6.050056243	5.921142536	5.98271401	6.05193401	5.98989174	6.07566842	5.92657118	6.00863095	5.8606078	5.9488603	6.0240031	6.0612177	6.07853847	5.86408043	5.923763841	6.118909056	6.056328649	6.156394326
Bra018008	9.70885908	9.708731954	9.30717796	9.24005763	9.49563394	9.42078994	9.55795998	9.65298223	9.4851286	9.4833882	9.5867045	9.3477077	10.0628521	9.93992323	9.695150041	9.838166085	10.09536492	10.51826324
Bra018009	8.673682217	8.763326738	8.62609257	8.14993033	8.5091236	8.0767734	8.51619414	8.90889598	8.4454386	8.2724578	8.4244376	8.3156258	9.43868752	8.91224229	9.072307337	8.952733978	9.001412849	8.880281276
Bra018016	7.37992658	7.480456854	7.16518161	7.17698874	7.37161052	7.18443157	7.61007096	7.68624277	7.5681337	7.5417874	7.4461946	7.5589775	7.38499516	7.42926567	7.645054233	7.693158176	6.79716347	6.782873589
Bra018019	7.022158408	6.961049756	7.26386629	7.01918546	7.35969855	7.2222316	7.52579346	7.35088891	7.7391262	7.4971789	8.0690604	7.846627	7.21374064	7.44790182	7.408279483	7.592376572	7.730635685	8.102488685
Bra018036	9.550726263	9.582338719	9.69119721	9.48577126	9.98105963	9.91878252	9.66358556	9.68406859	9.9821709	10.214344	10.035335	10.160989	9.75683987	9.82023608	9.929372218	10.03769517	9.909117776	9.778458252
Bra018037	7.228364945	7.142315134	6.50587239	6.92664402	6.71740374	7.22387253	7.57959011	6.97885448	7.5537284	6.7934655	7.5184163	7.2593302	6.75368524	6.71608652	7.2165485	6.940022228	6.979392111	6.668807348
Bra018042	6.885327471	6.976605698	6.47702015	7.21076752	6.9794147	7.64282826	7.17340883	6.77077514	7.5229919	6.8240272	7.7610831	7.8176992	6.83114843	6.93345101	7.344199956	7.384618668	6.794717852	6.571891768
Bra018055	7.249323657	7.139083236	6.85244925	6.93481489	7.21344571	6.67579471	7.07300909	7.55940431	7.3280433	7.4987434	6.9377753	7.1191112	7.1925958	7.12008304	7.10094504	7.322040087	6.858847543	6.804029071
Bra018057	6.951417546	6.90109993	7.77832616	7.68648674	7.01377756	8.07462515	7.21728708	6.90414353	7.1939841	7.4287464	7.4095392	7.8251063	6.61527861	6.65667741	7.509855174	7.119892044	7.233836572	6.941968149
Bra018058	5.99886843	6.037171971	6.12252439	5.98994085	6.11416937	6.07877276	6.11936465	6.06184514	5.9769502	6.1112711	6.0112571	6.2119126	6.1324204	6.05281659	6.196387654	5.971728947	6.397278388	6.107051041
Bra018072	6.005191954	5.959977159	6.10821878	5.9666871	5.97695069	6.10581817	6.03788157	5.86104021	6.1322162	6.0133863	5.9323215	5.9802823	5.95304588	6.04997828	6.005955173	6.011973765	5.96511418	6.262591063
Bra018073	6.502444051	6.521527201	6.67083439	6.38668717	6.52676639	6.44770607	6.25725598	6.55833631	6.3759442	6.285919	6.4138157	6.337838	6.56755113	6.41131986	6.596981325	6.46478909	6.637470635	6.348777282
Bra018084	7.475524144	7.48719073	6.76626607	7.00030047	7.53142046	7.41293081	7.6843493	7.01534841	7.3585461	7.7358532	7.7818486	7.4410759	6.91287068	6.94487922	7.052111336	6.97088079	6.881502845	6.869316502
Bra018085	7.061346606	6.845982496	8.31001617	7.78163563	7.22218998	7.8464392	7.78060468	7.81912648	8.2485087	7.9820828	8.0161071	8.0779484	7.62834128	7.4875288	8.102374903	7.49007389	8.825588143	8.256052276
Bra018123	9.254161336	9.114083013	9.03151723	9.05917297	8.65442422	8.59144797	10.0314945	9.62917713	9.4710352	9.9546714	8.9694942	9.1450654	8.4206428	8.66783498	8.41598685	8.388809475	8.086853953	8.364345052
Bra018124	6.165128538	5.957360935	6.10202798	6.11502304	6.07559283	6.08942683	6.12077496	6.15082121	6.2783354	6.1255769	6.1424762	6.1246503	6.13594522	6.08185699	6.164936616	6.103382487	6.235285701	6.031708507
Bra018125	6.338019377	6.809292938	6.8041189	6.58891309	6.8828459	6.6767613	5.93290807	6.01072376	6.0569629	5.8625286	5.9468793	5.9119214	6.3731074	6.28433469	6.513782834	6.578784673	6.254996902	5.968435836
Bra018149	12.03823563	11.86426415	11.7467491	11.1535873	11.50086	10.5803968	12.0946631	11.8524451	12.150276	12.437582	11.050587	10.891809	11.6935445	11.4562887	9.174141444	8.701071929	7.96333001	8.846698149
Bra018150	6.193013495	6.159550631	6.09692446	6.04401381	6.19051422	6.10657516	6.24828515	6.21040544	6.4723982	6.5964259	6.4005965	6.4094036	6.20129103	6.13339697	6.357479704	6.237494273	6.563777984	6.554218258
Bra018151	9.208942239	8.769368817	9.42414023	8.7496454	8.76128397	8.69694028	7.83049689	8.04469006	7.5169839	7.3808563	7.5089766	6.9778464	9.1137903	8.96695139	9.75215968	9.194763056	11.416964	11.76759191
Bra018159	6.309902822	6.43179681	6.22766295	6.1852525	6.22338729	6.20784672	6.12879754	6.24150451	5.9888265	6.1702846	6.158846	6.1400822	6.06326498	6.03422771	6.107335279	6.101151835	5.887191133	6.061656143
Bra018160	5.990360852	6.171959774	6.38245582	6.6354662	8.67055665	9.21244845	6.42450264	6.30364303	7.9854822	6.8609431	10.577271	10.864206	6.50503553	6.26088274	6.876175451	6.815262229	7.165523516	6.89811832
Bra018312	6.058696579	6.18195575	6.24744511	6.00542027	6.17394674	6.2078796	5.94413306	6.22107214	6.1248996	6.0193341	6.1280328	5.9432953	6.12560148	6.11724256	6.109960952	6.111994047	6.113535903	6.124272968
Bra018313	8.101337405	8.024277208	7.85186829	7.38746113	7.81740312	7.62277968	7.85196848	8.11159641	7.9263947	7.8253677	7.8152688	7.6752289	8.78126702	8.4667764	8.49078631	8.256814121	9.369701617	9.265308976
Bra019332	6.557837619	6.387236623	6.62895886	6.58497582	6.5507161	6.73103293	7.21160354	7.24190289	7.4345535	6.9946865	7.6047912	7.1269376	7.046093	6.91784696	7.066103349	6.86107483	8.067948373	7.581844257
Bra019593	5.972942063	6.167877048	6.15043862	6.0888181	6.03003803	6.09674291	5.86802308	6.0027751	6.1188354	5.915107	5.9044116	5.8446406	5.92668162	5.99141471	6.000976542	5.982122054	6.018815965	6.051299329
Bra019658	6.59080312	6.592588346	6.81144881	6.41466778	6.91344105	6.5473138	7.0365385	7.1369843	7.3982136	7.0999411	7.0175876	6.7839435	6.58901226	6.42069762	6.786257716	6.66704993	6.989373682	6.793839593
Bra019659	9.682909283	9.601428911	9.17994991	8.93907995	8.5705496	8.36145173	10.0753524	9.72097703	9.0876683	9.4050101	8.4930596	8.6600969	10.2622255	10.7845347	10.49627456	10.21339626	9.362387625	9.106172641
Bra019660	7.239548119	6.897284544	7.89939215	7.63498252	7.78624823	8.34163917	7.29110727	7.18977454	7.6222854	7.9723039	7.7892984	7.7768907	6.19214377	6.2943566	6.363976898	6.329180131	6.305079525	6.197721718

Bra019705	6.121030697	5.828318165	6.04079779	5.95710937	6.02561767	6.02616949	6.09011304	6.39769522	6.4338533	6.4564137	6.2320588	6.4674956	6.1487369	6.23439689	6.320343607	6.347058234	6.305480236	6.163877269
Bra019707	9.925944222	10.17058703	8.9742264	8.80571985	9.06790229	9.04921738	9.25848547	9.35146072	8.9630252	8.9168278	8.6972466	8.8078633	10.0375699	9.86497928	9.582280926	9.568322647	9.500134222	9.381160972
Bra019751	5.777151234	5.996253448	5.94855134	5.99776252	5.99121002	6.04219026	5.90344211	5.93111318	5.7777766	5.9466642	5.9650323	5.9968859	5.93152358	5.89752144	5.924452688	5.845677595	5.925748334	6.143110743
Bra019774	7.224138377	7.203060736	6.92095004	7.04865944	6.81710962	6.85874158	7.30064029	6.9174635	6.7883659	6.9349249	6.88474	6.94183	6.89743666	7.0131843	6.847266416	6.864975455	6.991675436	6.842313154
Bra019787	5.847129403	6.029945681	6.0075221	6.01288786	6.03606709	6.08835254	6.23553542	5.92471221	6.1179581	6.1644456	6.2519361	6.0757698	6.084848	6.05467581	5.955321875	6.017971031	6.256805744	6.182466085
Bra019819	6.259256628	6.21816894	6.26194472	6.12771625	6.17270302	6.17248113	6.20339136	6.33901424	6.3755881	6.2601529	6.2072432	6.2392305	6.34115075	6.19479924	6.37010944	6.228040764	6.745785898	6.201119438
Bra019824	5.814761157	5.857565899	5.9170763	5.85588485	5.87076474	5.79313596	5.88572508	5.88638101	5.9915607	5.9683078	6.0916315	5.8447661	6.09100403	6.04239915	5.98825793	5.919436538	5.963259134	6.143835317
Bra019828	8.405618495	8.524032554	8.25236251	8.15880086	8.57184702	8.44912303	8.71125647	8.63014038	8.5254408	9.0149166	8.4220724	8.6659387	8.29343679	8.44230354	8.386916982	8.379608339	8.698294744	8.873343785
Bra019829	7.723697743	7.629994077	7.75738474	7.64631784	7.41254125	7.8146558	7.50668417	7.47275866	7.3491529	7.3743405	7.4229461	7.3230783	7.46548808	7.27379674	7.709176974	7.448862528	8.249395822	7.656639354
Bra019830	7.540403824	7.506843028	7.60279457	7.53835332	7.48637955	7.48150222	7.47146463	7.38241493	7.0185347	7.1512551	6.9955169	6.9990689	7.0025756	7.12534341	7.248510468	7.240836183	6.669475689	6.457626243
Bra019843	5.76324076	5.889343121	5.90567737	5.81539587	5.7809027	5.9341113	6.02961847	5.82684327	5.8539893	5.8707128	5.8568098	6.0472737	5.90601127	5.88206826	5.859378807	5.851183015	5.980698598	5.898254078
Bra019844	5.892738582	6.080285762	6.04241157	6.00695406	6.07520037	6.04887782	6.14179641	5.94754084	6.0681726	6.0624974	6.1183092	6.1126766	6.1150217	6.0846424	5.96461054	6.015651261	6.029848783	6.154625979
Bra019885	6.257771543	6.167911991	6.13864093	6.09390434	6.27941844	6.1396003	6.30449586	6.27813228	6.1829666	6.1025687	6.2407154	6.1968	6.24837967	6.39045413	6.321029015	6.239539566	6.486140313	6.227399981
Bra019906	7.729083737	7.603965929	8.60948351	8.57111281	8.29652448	8.77579817	7.39072631	7.85882353	7.7975851	7.7611894	7.8470683	7.9628453	8.00887565	7.91784222	8.354898462	8.296599135	8.301154172	8.437996645
Bra019950	7.03062846	7.045144054	7.27153953	7.26786657	7.16515422	7.06031421	7.17595607	7.39486649	7.2350481	7.3382028	7.0697785	7.1824323	7.36682655	7.3215799	7.393595839	7.063818107	7.626755301	7.296736357
Bra019957	6.154316573	6.056283687	6.0960747	6.2374246	6.15769032	6.00459744	5.94402308	5.93367902	5.920695	5.9844583	6.0475721	5.9012169	6.05201526	6.04640906	5.99264318	5.885327087	6.04343486	5.884119506
Bra019958	6.428932327	6.419922777	6.2189708	6.43747461	6.05906518	6.2375147	6.44251596	6.48669424	6.3703389	6.1636447	6.3432915	6.2454097	6.37189355	6.27374925	6.539002237	6.407545848	6.695679529	6.334164094
Bra019970	6.172867889	6.246578341	6.18171754	6.18036198	6.33824787	6.19246512	6.37510269	6.57960021	6.5964778	6.345006	6.418566	6.325816	6.42561131	6.38905657	6.37048382	6.359514459	6.331365979	6.317897538
Bra019975	6.993708738	6.88691067	7.2301615	7.49968346	7.53239196	7.44925387	7.10289196	7.04093155	7.1837071	7.3579096	7.3963102	7.5069516	6.52774141	6.66202168	6.930132958	6.983824178	6.552555025	6.606851848
Bra020751	10.16344766	10.20532984	10.6324448	10.3177817	10.5646757	10.4086187	10.0321019	10.5025259	10.346618	10.467769	10.115852	10.490494	10.6581706	10.5318791	10.77252	10.82036374	10.52873065	10.22196028
Bra020868	7.0451506	7.132547457	7.87108881	7.62478187	6.84267312	7.37947477	6.52992873	6.39874404	6.2620806	6.4208887	6.270934	6.5080731	6.6490902	6.69772338	6.915846795	6.690167834	6.911076926	6.680520083
Bra020959	6.905527762	6.866743921	7.46171866	7.32435058	7.01237407	6.90980552	6.83589934	6.79093266	6.8861186	6.8115521	6.8987978	6.8250941	6.63393921	6.54250069	6.688457912	6.692390557	6.527358467	6.535877541
Bra020998	6.135731169	6.009715967	5.9715491	5.99513535	6.16942602	6.15205251	6.35655241	6.15224438	6.228741	6.3526711	6.2504357	6.3843709	6.27620507	6.06548043	6.125924421	5.987493469	6.129567579	6.165524585
Bra021048	6.125402216	6.051223184	8.79394406	9.11834383	6.48841759	8.46263638	6.03474118	6.15077214	6.5059675	5.9510507	7.2956153	7.5649572	5.99212299	6.05158189	6.12193006	6.260264568	6.228644592	6.10711839
Bra021049	6.045756018	5.870619691	6.24894845	6.51484009	5.88842019	6.5951624	5.97541593	6.03416295	6.1403609	5.9147765	6.3801904	6.9585384	5.95045634	6.06157736	5.845828242	5.982732644	6.187065453	5.937706672
Bra021699	6.565279715	6.315638396	6.44093481	6.63156843	7.66140641	7.86069148	6.15490994	6.42706455	6.816285	6.7018065	6.7402013	6.6630059	6.64228653	6.84981797	6.664761247	7.057074605	6.839384749	7.186168717
Bra021765	5.957091565	5.986752814	5.86737979	5.95836236	5.86151301	5.93043391	5.88552526	5.88401607	5.9452117	5.8164893	5.9807554	5.8372375	6.0206388	5.97500297	5.944897234	5.820821253	5.907184824	5.874713403
Bra021817	6.275526682	6.290023714	6.14426794	6.14892566	6.20687889	6.11425751	6.48613154	6.59406043	6.5781959	6.3974	6.524578	6.4232648	6.14740501	6.09366085	6.252262144	6.286802535	6.251314206	6.328066407
Bra021818	5.981032746	5.901227108	5.89787291	5.94319782	5.88570299	5.89877113	5.93204345	5.82376938	5.9567361	5.9196523	5.8475385	6.0937634	5.96573598	5.92047268	6.009348255	5.923629362	5.958131832	5.946229727
Bra022194	6.314610684	6.302253651	6.32339608	6.21126869	6.20702113	6.22896868	6.34285349	6.26294621	6.2206085	6.0247624	6.2413536	6.3239668	6.61415687	6.50908891	6.221970552	6.274999313	6.295439391	6.398662939
Bra022195	7.364420689	7.458246171	7.37131713	7.4565475	7.05728851	7.16580689	7.27305923	6.86774567	6.8206282	6.6594233	6.6836066	6.6217028	7.11442648	7.27884596	6.969331563	6.793426551	6.945247552	7.122657019
Bra022235	6.568929529	6.542468706	6.51327752	6.71126994	7.45458556	7.46074315	6.71247194	6.61605168	6.9526322	6.7307516	7.4700845	7.0540395	6.68257451	6.51210179	6.552995287	6.573206528	6.579094681	6.671920812
Bra022241	6.065661571	5.902878156	6.05053579	6.00866371	5.87482012	5.91384972	5.96413021	5.85334175	5.9905402	6.0287871	5.8919183	5.9199429	5.99226616	5.99645096	5.914054172	5.927746033	6.114349844	5.950951772
Bra022242	6.404458295	6.58301057	6.34543332	6.37687964	6.25825218	6.45666429	6.4260557	6.46633513	6.3143604	6.3357877	6.4781616	6.5267325	6.11898296	6.11960392	6.397880988	6.289694687	6.321833218	6.055546101
Bra022265	5.912395662	5.886946747	6.10957557	5.83387667	5.89677172	5.87614833	5.96511169	5.90839698	5.9993008	5.9542112	5.8334907	5.8573952	5.98705729	5.98455799	5.834117462	5.928498129	6.003618711	6.004728721
Bra022266	6.639155755	6.629751851	6.09760363	6.07463825	6.05372738	5.9543977	6.41334301	6.69228796	6.0577793	5.9932341	6.1140841	5.9417249	6.94845223	6.8942527	6.212813175	6.366092438	6.102561806	6.252321002
Bra022270	5.937215886	5.915183902	5.79695758	5.86092758	5.84036777	5.99132403	5.95003482	6.03634965	6.1171414	6.0651187	6.0160968	6.0275101	6.07657689	5.9553881	5.936622361	5.842040787	6.069239918	6.023157667
Bra023221	5.901989882	5.814737751	5.94359912	5.99409118	6.05453562	5.91524331	5.78540173	5.95723185	5.7302299	5.7471808	5.9070881	5.9277699	5.85197539	5.76122983	5.810850071	5.875600649	5.943250425	5.931229735
Bra023297	5.80516053	5.915591433	6.07070836	5.86983931	5.90713798	6.06214697	5.82818679	5.88263874	5.7897731	5.7506539	5.7922715	5.9178038	5.7728245	5.7670081	5.933393801	5.894441291	5.921662436	5.810218852
Bra023298	5.960768646	5.881804131	5.83080666	6.09219241	5.88999951	5.99069347	5.97623766	5.98192115	6.7539408	6.9807919	6.6256848	6.5604801	6.01962121	5.85499404	5.901374912	5.873753524	5.963006462	5.733031459
Bra023345	6.022681962	5.954110297	5.9299633	6.06229491	6.0751946	5.97042367	5.87648816	6.10737822	6.0321338	5.9283228	6.1412883	6.0685206	6.11489805	6.06733876	6.091709739	6.061219165	6.046759715	6.269429171
Bra023346	6.035003769	5.995873717	6.17315698	5.98912386	6.05647313	6.11593001	6.01443682	6.22123855	5.9825143	6.0543654	6.0525188	6.0681375	6.06568282	6.087259	5.985428049	6.068289407	6.082989079	6.156441239
Bra023348	6.022997224	6.109218964	6.12256653	6.07339017	6.08677233	5.98616029	5.98679861	6.06348331	6.0178651	6.0242561	6.0727518	6.0010374	6.11684714	6.08404375	5.894108754	5.998097288	5.938290031	6.126092145
Bra023694	7.65206524	7.93972168	7.1714589	7.22631895	7.34674984	6.90249351	6.49527939	6.52813418	6.4076766	6.40853	6.273195	6.4047875	6.43797702	6.53055255	6.363586724	6.412730862	6.09399277	6.138054261
Bra023750	5.846982494	5.99853083	6.03959474	5.96952538	5.97831637	6.09067959	6.03114386	5.81715699	6.0295853	5.9489577	5.9537533	6.0281662	5.94515114	6.00144534	5.967934945	5.82382098	5.915137895	5.889069491
Bra023751	6.093400407	6.24069776	6.15511595	6.1811673	6.19539006	6.17592842	6.40443329	6.49565159	6.5068874	6.7867932	6.6085021	6.5611917	7.50877609	7.37509816	7.523700121	7.987268585	6.746362473	7.157904402
Bra023789	8.415825056	8.390642068	8.62505559	8.39229552	8.06276615	7.94349267	7.07604892	7.00139362	6.8942549	6.944751	6.8409935	6.5947468	8.08151984	7.90154263	8.257280496	7.750490708	8.993205287	8.243984749
Bra023790	6.748991007	6.87745042	6.96335922	7.065482	6.62187564	6.81175403	6.40437032	6.34866571	6.3719445	6.3669329	6.4320614	6.4133195	6.52033086	6.57764676	6.268979366	6.233308979	6.529875373	6.551152471
Bra024186	7.334723295	7.312094703	7.18088524	7.26626901	8.23648001	7.14750912	7.3164074	7.64162189	7.4787145	6.6905412	6.8174	6.595789	7.4104656	7.07434888	7.158331377	7.018996861	7.154001293	6.588543356
Bra024241	7.465934592	7.276644898	7.77125952	7.23708061	7.35764161	7.31819872	6.60892652	6.67229277	6.4027097	6.5729003	6.5684875	6.5116308	7.42656719	7.28062609	7.330599542	6.952794809	7.805508431	7.139694212
Bra024289	7.542831927	7.299709786	7.28647445	7.30087483	7.2786818	7.20899766	7.27288508	7.32850492	7.4216263	7.1948115	7.0159	6.9488686	7.43915708	7.52340139	7.086067838	7.450098787	7.098002292	8.103443557
Bra024329	8.201219159	8.220891587	8.35306544	8.34172556	8.4790912	8.32128021	8.46622353	8.24524726	8.4875448	8.2773526	8.4203381	8.4348283	8.1217117	8.23180826	8.444259645	8.327505547	8.53875784	8.19530549
Bra024406	7.67404004	7.573736095	7.31922826	7.83147854	8.0924745	8.06592558	9.16220486	9.07642312	8.939369	9.0449346	9.1267164	9.2907322	7.34633274	7.70131492	7.592799401	8.238249442	6.558296865	7.117410292
Bra024407	7.137615513	7.410709312	7.46656616	7.37353803	7.10334151	7.0742554	6.05691308	6.06676601	6.0729246	6.2364457	5.9932099	6.1901437	6.33501013	6.27431957	6.75432152	6.757663921	6.563137098	6.504877624
Bra024408	6.384361041	6.353339436	6.25918053	6.33270195	6.34896116	6.32394367	6.6815169	6.6290654	6.54419	6.4343226	6.416421	6.405925	6.58844356	6.60231633	6.302283965	6.513380139	6.408130306	6.78430469
Bra024422	6.169729849	6.270149323	6.05888596	6.10224747	6.11600955	6.08895186	6.16875309	6.36486237	6.2920581	6.1283119	6.153751	6.0432748	6.3067972	6.18439799	6.219097001	6.143547673	6.046623977	6.128642832
Bra024439	8.984400658	9.268342539	8.77907501	8.51977756	8.29061808	7.93221941	8.85931717	8.89672104	8.4187589	8.4853206	8.0684449	7.9675623	8.74582497	8.57078535	8.109519805	8.319620358	7.866929668	8.02981981
Bra024485	6.113436842	6.163329419	6.01861459	6.08871321	6.11135811	6.12083171	5.91437721	5.94644031	6.1828163	6.063089	6.1890181	5.9860893	6.17149121	6.11286659	6.014779141	6.102730991	5.922797631	6.087332935

Bra024497	6.350648607	6.396997835	6.29563693	6.26039535	6.29325814	6.19679688	6.21818138	6.10691918	6.2263968	6.1546403	6.2053442	6.1755129	6.10559995	6.19504004	6.251480686	6.129631698	5.969032521	5.920892672
Bra024498	12.07101194	11.77403042	11.4644251	11.0979382	10.8969591	10.9246904	12.4683751	11.2016108	11.025601	11.267478	10.659528	10.508225	11.722561	11.9372096	10.89285109	11.31145231	9.76564763	11.36714636
Bra024768	5.834058617	5.87463823	5.95630017	5.89544782	5.83262537	5.85559278	6.01019267	6.07336448	5.9258514	6.0326664	5.9134946	6.041806	5.91130579	6.04233484	5.876732943	5.870395073	5.957611732	5.921447746
Bra024769	5.93414719	6.037699032	6.0855246	5.93385981	6.06636807	6.02685019	5.8966131	5.91426769	5.9114971	5.995464	5.992802	5.8736192	5.93354648	5.8630526	5.929536535	5.87801478	5.924175668	5.858648568
Bra024779	11.45400169	11.49260325	10.9916334	10.784655	11.1787734	10.9333603	11.7014906	11.2638218	10.952472	10.796424	10.57191	10.385138	11.358319	11.061754	11.07000804	10.82020581	10.98102025	10.78218216
Bra024814	7.352630737	7.19987594	7.37129646	7.47700881	7.38276351	7.41959472	7.1716662	7.18515477	7.0437439	7.0001966	7.0195426	6.9312318	7.53915375	7.43502021	7.13383077	6.920920872	7.707971398	7.567377283
Bra024818	7.642752081	7.414254447	7.48798943	7.21520812	7.70952087	7.35813327	7.53757936	7.71016271	6.777734	7.1733099	7.1823772	7.1955525	7.28941079	7.52272841	7.79039325	7.784204866	7.762097357	7.743780759
Bra024819	8.291308921	8.319216763	7.61838708	7.50785624	8.06097899	7.8654407	8.33056366	8.23859017	8.2108262	8.4455863	7.7025569	7.7611126	7.89063197	8.00938639	8.196283694	8.184300409	7.253400115	7.089425696
Bra024941	5.903962172	5.925172714	6.07428083	6.0109235	5.939577	5.95779513	6.01660468	5.96605635	6.0188053	6.0558866	6.0348125	5.9925993	6.0061354	6.14570834	6.049138018	5.939583451	6.010983304	6.031508353
Bra025016	7.461738335	7.89898937	7.69334274	7.42510842	7.90307694	7.66156279	8.14530585	8.1146069	8.0747225	7.7481756	7.8177312	7.610575	8.21373183	7.91503053	8.124507703	7.999632747	7.690597263	7.44224416
Bra025114	6.825466025	6.661874784	6.56430717	6.72625008	6.64196912	6.32913394	6.17965096	6.19060834	6.2003386	6.2537708	6.1399666	6.2295373	6.46803276	6.44356857	6.560761641	6.470168963	6.730687714	6.587498535
Bra025115	6.803108894	6.643687178	6.81849744	6.53193345	6.69297886	6.50084246	6.45968909	6.42643069	6.4252351	6.681895	6.399347	6.3054224	6.54194363	6.50966909	6.672561227	6.609410187	6.698328329	6.240623034
Bra025118	5.877483682	5.921654171	6.00698789	6.0062717	5.9917441	5.95182545	5.85321204	5.9990497	5.9193592	6.2010173	5.9255797	5.9265476	6.06140052	6.03321854	6.019359403	5.923048038	5.928998751	5.918480615
Bra025126	6.071895534	6.033369927	6.09703044	6.02453322	6.10940337	6.01678004	5.99742951	6.16899757	6.0758142	6.2507805	6.0274093	6.0657644	5.92750636	5.9183214	6.046737897	5.973447191	5.956552539	5.996496202
Bra025128	6.34988848	6.270544854	6.36488357	6.34692361	6.25498199	6.39868565	6.16519191	6.20351505	6.0704042	6.1956594	6.3097887	6.2520033	6.4388101	6.20831534	6.169577671	6.072288013	6.200439412	6.338204149
Bra025158	6.869536022	7.101372249	7.14643384	7.19528066	7.26377088	6.85486369	6.70719438	6.69701809	6.8672858	7.000043	6.7120635	6.8519671	6.75768557	6.89442955	6.775613252	7.077095981	6.544244067	6.558982285
Bra025159	6.91039387	6.923209522	6.75566022	6.96583228	6.80212549	6.80891284	6.50030822	6.5900645	6.4612073	6.5377645	6.6667783	6.6447453	6.15897988	6.58150923	6.706840027	7.050462877	6.293431526	6.161041439
Bra025165	9.514963114	9.330615763	8.27556642	9.38803447	8.00249069	9.33085209	9.91918584	9.44531681	10.19841	9.4577761	9.9135725	9.0330243	10.5209958	10.0784601	10.36136755	9.788716872	11.84021678	11.99701957
Bra025166	6.880120796	6.666465809	6.92906021	6.65919996	6.79153222	6.65724599	7.31904207	7.0639586	6.6159081	6.8280993	6.6862191	6.7181056	6.62781691	6.68119123	6.673551718	6.621033573	6.731813166	6.667484159
Bra025168	8.278532023	8.368093704	9.65111778	10.0174602	8.92745138	9.44128044	8.76031804	8.51709191	8.4660276	8.3126657	9.9254739	9.0077093	9.40542322	9.35255648	9.488639618	9.308121443	8.915726409	8.514874383
Bra025169	7.325317612	7.139712121	7.06414061	7.53532091	9.3617489	8.99689959	8.71789442	8.53735453	9.7105744	9.1491937	9.5065842	8.9445119	7.37684837	7.49161255	8.613125384	8.743445568	9.215112585	8.48881137
Bra025278	7.565255517	7.386534615	7.92984686	7.82928093	8.54594049	8.080802	7.91060574	7.85100842	8.174725	8.2919691	8.4822914	8.4239317	7.7077989	7.47041358	8.161825374	8.006510397	8.426297392	8.176620995
Bra025315	7.516440383	7.577329374	7.13188278	7.391639	7.64554129	7.9942577	8.17829584	7.71280373	8.1493215	8.3586661	7.8767535	7.9092802	7.39279572	7.42320714	7.726407996	7.749194277	6.842660988	6.860867012
Bra025337	6.27802212	6.195160523	6.15071016	5.98791163	6.26206098	6.14394695	6.44156036	6.25949635	6.2474841	6.2007848	6.2235171	6.2545095	6.21304831	6.23246796	6.447604198	6.2969174	6.282436255	6.246588543
Bra025371	6.373488655	6.531944005	6.46095454	6.36882056	6.49232426	6.60281052	6.41861649	6.34610941	6.1691288	6.3839089	6.2106086	6.373726	6.25409284	6.32450469	6.447076937	6.448497269	6.68832959	6.35351549
Bra025394	9.551255699	9.505247501	8.96534468	8.85767981	9.25713921	9.18453058	9.61430638	9.54680979	9.413946	9.0356878	9.4156547	9.3837489	9.65552359	9.69625549	9.867907643	9.991588368	10.27382735	10.24142295
Bra025681	8.971816511	8.19052072	9.76547359	9.10703391	8.13922574	8.51853341	8.43182131	8.11017558	7.4533711	6.7026691	6.8293867	6.961845	9.58160131	9.51575101	8.369856481	8.127547792	7.134542603	7.218797342
Bra025682	8.175881143	8.237018771	7.83844672	7.90785388	7.84447951	8.07166868	8.80261067	7.57038984	7.8591445	8.1468657	7.5376264	7.4729564	8.10090785	8.41251528	8.000500275	7.681614129	7.236541398	7.153760834
Bra025697	6.297685714	6.35984442	6.01503212	6.25496065	5.97689538	6.17360707	6.1626241	6.01351174	6.1130279	5.9450771	6.1790217	6.0611752	6.30782418	6.25365301	6.356586441	6.140898167	6.227937549	6.231106219
Bra025725	6.125192999	6.139458289	6.27230523	6.19152523	6.20876775	6.19546399	6.32769359	6.28644042	6.4413155	6.4766265	6.2533713	6.328115	6.29399521	6.10252627	6.270646738	6.416076543	6.360953707	6.327343344
Bra025730	7.093488418	7.423802168	8.99552915	10.228503	10.6418387	11.6517257	9.22145193	8.78178293	11.555577	10.240104	13.19062	11.944775	7.94294615	7.9141056	9.753055625	9.035344112	11.1695324	9.594351809
Bra025731	7.611668917	7.353151921	8.48959162	8.28425081	8.63120642	8.76126234	7.90528424	8.01509221	8.6364798	8.5601875	8.7686445	8.9748566	7.61190506	7.43689765	7.8720544	8.125679099	7.693696647	7.905991115
Bra025736	6.073567079	6.160811072	5.92550425	5.96224688	6.06557396	5.97625839	6.18482883	6.03412908	6.2138092	6.1277332	6.1212112	6.0593151	6.09084839	6.02039498	6.040656806	6.000608151	5.938780371	6.149685791
Bra025769	6.337217365	6.328321248	6.41960326	6.38350701	6.82091296	6.42847779	6.26594499	6.26882501	6.5317915	6.5198913	6.3758085	6.6045232	6.91001839	6.05812078	6.456736881	6.553613357	6.198153509	6.113515788
Bra025772	8.781549602	8.97880858	10.3706121	9.86583556	9.30773871	10.0646899	9.28711047	9.38927734	9.6691639	9.5995128	9.4316992	9.4920626	9.38878384	9.14128015	9.680502687	9.384188757	10.2837995	10.20489821
Bra025773	6.283184959	6.247034226	6.46634198	6.20530662	6.35856677	6.21732141	6.35879206	6.46788464	6.2737904	6.3336315	6.3647727	6.2348409	6.58640523	6.55201122	6.478752553	6.420567578	7.261885973	6.793017086
Bra025774	6.949963143	6.976933661	7.15320218	7.10796958	7.82675332	6.52446647	6.97150287	7.11639821	7.6221732	7.9372421	7.4937124	7.2625804	7.02950937	7.07308274	8.206338391	8.290393751	8.075478393	8.078929674
Bra025775	6.508344846	6.345628848	6.54124792	6.36679217	6.5370828	6.37911416	6.17306371	6.25090661	6.222815	6.1554193	6.1498877	6.137669	6.37738116	6.42845129	6.42293657	6.488438284	6.524110929	6.450245128
Bra025779	6.291990802	6.373122592	6.4493016	6.24797251	6.3724712	6.17582802	6.07510369	6.25119481	6.1420207	6.2533706	6.0450318	5.9886495	6.18103627	6.06167374	6.315892795	6.150882173	6.360720963	6.178639963
Bra025780	5.904180474	5.933189829	5.96072343	5.90121579	5.92463116	5.88064722	5.94581472	5.91384908	5.886884	5.9378974	5.7975641	5.9734118	5.99890672	5.80916573	6.017953911	6.031191258	5.919242688	6.010578138
Bra025787	10.53508631	10.56316999	10.1464723	9.82782418	9.76484299	9.47105693	9.75489433	10.2950162	9.9391286	9.6629584	9.3993032	9.1465504	11.0664666	10.9278495	9.418271811	10.1749397	9.225836959	10.29281807
Bra025820	7.941926803	7.831760662	8.13723059	7.96974399	8.41174702	8.14014421	8.21949791	8.11237806	8.383515	8.2614899	8.3216118	8.2475425	7.80432725	7.7189763	8.328000194	8.207462649	7.235813861	7.047603401
Bra025827	6.829523598	6.821911148	6.64566412	6.64441269	7.14978804	6.85583918	7.11720684	7.00699363	7.1130644	7.3377509	7.1591885	7.3956485	6.61551238	6.67285609	6.986131421	7.349437998	6.707949314	6.919264531
Bra025831	6.315656557	6.27162716	6.30894468	6.26026301	6.68775709	6.3321179	6.30976276	6.31152961	6.1405664	6.0708274	6.1136718	6.0667063	6.33310364	6.37665784	6.577070744	6.521001609	6.23261789	6.118295507
Bra025834	8.866099968	8.723445221	9.00208346	8.5896841	8.62818983	8.63152317	9.2661679	9.06042898	9.2122246	8.9093418	9.3454225	8.8813927	9.53628083	9.4082527	9.669866246	9.307353368	10.68039995	10.03759114
Bra025835	6.631510993	6.67298238	6.60091608	6.33118922	6.74545944	6.42759514	6.90124408	7.10123524	6.9327119	6.7622772	6.4392875	6.4076209	6.8942174	6.58006058	7.762727672	6.506659932	8.181653355	6.957856335
Bra025842	6.663485229	6.746928798	6.59218003	6.71455369	6.842011	6.76477817	7.482961	7.46439275	7.2576802	7.4448444	7.3020206	7.4078087	6.70392541	6.8496976	6.979783415	7.170316053	6.979415382	6.902771457
Bra025925	6.147547268	6.019487857	6.23898611	6.15237918	6.13644836	6.1155889	6.11047404	6.16777347	6.1759525	6.1795573	6.1737238	6.1437672	6.45080764	6.25652513	6.309782059	6.258368102	6.487280871	6.359280832
Bra025938	6.168666074	6.146390294	6.25414645	6.22645401	6.15850934	6.15795737	6.01979242	6.07876761	6.0278917	5.8789303	5.910871	6.0268032	6.19458102	6.21463454	6.136159484	6.156983253	5.978641035	5.938721772
Bra025951	6.23895928	6.195603763	6.22507096	6.23122196	6.19541502	6.29174501	5.96552078	5.90640913	5.8617763	6.0157435	5.9935421	6.0794441	6.10897555	6.00921017	6.134513577	6.173627264	6.104406933	6.017975196
Bra025952	6.397476988	6.30152374	6.56731797	6.52978292	6.59535516	6.58801446	6.63962893	6.5985143	6.4208138	6.6019837	6.5492752	6.6794471	6.44614019	6.40531313	6.547349867	6.693925619	6.505257585	6.678007271
Bra026010	6.008455864	5.969976382	6.01814456	5.97672502	6.04098191	5.99087336	5.97830733	6.13600862	6.0817206	6.1292939	6.1301736	5.9281906	6.08191692	6.18422892	6.137925094	6.128344604	6.195334963	6.160881424
Bra026011	7.608086727	7.194501259	7.58949039	7.1675255	6.84890558	6.74785424	6.86870479	6.8978037	6.53481	6.4425684	6.4149975	6.1257708	7.45894151	7.41978438	7.256397966	6.590155033	8.498938698	8.363227683
Bra026014	6.016572196	6.046171135	6.10437605	6.05818669	6.09680994	6.19356386	6.20071816	6.05953913	6.1787974	6.1218644	6.1414457	6.1210526	6.02068498	6.08416625	6.241582224	5.974683456	6.333023286	6.130146684
Bra026015	6.434780589	6.447866753	6.11859854	6.53281525	6.18062089	6.18683922	6.21264506	6.11767182	5.9892768	6.1818197	6.0845633	5.9980811	6.11534032	5.9275885	5.93505844	5.983306345	6.158899107	5.976943748
Bra026024	6.239015278	6.211322556	5.9244772	6.04181155	6.00168355	6.01576161	5.9659006	5.99153543	5.8898774	5.9155963	5.8602682	6.011336	5.94716662	5.96724679	5.922878662	5.98825721	5.788182378	5.930103127
Bra026025	9.825499015	9.785916554	10.2469641	10.2470943	10.295004	10.2493791	9.95228998	10.1165961	10.095	10.247403	9.9996008	10.111124	10.0175776	9.94188545	10.21123113	10.20189242	10.0495219	9.793061831
Bra026041	6.163242054	6.075834353	6.03806504	6.08116147	6.12433387	6.03928407	6.06415873	6.08404669	6.122045	5.9764747	6.1790759	6.2501205	6.18969222	6.08259123	6.204324964	5.98451341	6.271197102	6.133368918

Bra026055	7.975806818	8.703456619	8.05802164	8.54446928	8.25200927	8.33737973	8.4024637	8.418576	8.3210178	9.1122038	8.4200731	9.0127155	7.90479551	8.19645831	7.918869269	8.252409296	7.87129549	8.099178199
Bra026072	6.44416842	6.537961192	6.68952738	6.56468384	6.63873665	6.59230611	6.30695997	6.3439375	6.3134105	6.2823271	6.1674771	6.346505	6.23019596	6.19878774	6.199888443	6.370014972	6.130291444	6.259448862
Bra026078	7.043557227	6.703107084	8.21297021	7.59015516	8.47891617	8.40079219	7.62337996	7.48896887	8.2965926	8.2860752	8.2830912	8.359518	7.209228	7.21968884	7.745089152	7.382508358	7.896986987	7.641082728
Bra026094	5.876018349	5.725039344	5.95877849	5.86606331	5.95108248	5.91107244	5.80378702	5.97579263	5.995028	6.2069445	5.8532183	6.059139	5.94829739	5.91415162	6.061491065	6.195519264	6.100544587	6.028071029
Bra026108	6.311428532	6.193599435	6.37111247	6.24544643	6.19741034	6.30968691	6.22732154	6.2089719	6.2084685	6.2342013	6.386204	6.2044557	6.17440824	6.33008261	6.035428995	6.668878789	6.06866582	6.520130855
Bra026147	6.610061179	6.665503761	6.77964452	6.49948624	6.54585371	6.60418564	6.86590994	6.89799385	6.8767655	6.8357923	6.7898563	6.6495264	7.03171987	6.8554421	6.478338922	6.414442773	6.626779682	6.654084975
Bra026150	11.78516228	11.75029515	12.1346696	11.9501524	11.8622647	11.8492875	11.688301	11.5631101	12.09569	11.889812	12.082357	11.879967	11.7385908	11.5194433	11.85415831	11.81038684	12.15427866	12.01026792
Bra026152	6.328177903	6.164097124	6.19602795	6.14945089	6.07081513	6.13816191	6.19938032	6.39592358	6.4983506	6.1852295	6.3043489	6.3347483	6.31281445	6.2088532	6.340657136	6.20085437	6.643018945	6.641565834
Bra026183	5.98937587	5.925560834	6.17679274	5.82986961	5.93242425	5.98859979	6.07817729	6.05419972	6.1252095	5.8387716	6.0515603	6.0674636	5.95300615	5.91862789	5.959374668	5.974619855	5.993338354	5.985993624
Bra026314	12.76396606	12.904845	11.7305297	11.7626472	10.9891255	10.5065944	12.9733275	12.484604	11.192532	11.658271	10.824742	10.871527	12.4022213	12.9194431	10.80993739	10.57517531	9.428708701	9.170048789
Bra026320	5.945233774	6.029366641	6.11430347	5.84115243	6.01962735	6.01434653	5.95449921	6.23480777	5.9652876	6.1011968	6.0164582	6.0827309	5.94116074	6.04300089	6.058993971	5.826909615	6.036096195	5.952866424
Bra026321	6.988217874	6.886888278	6.73875859	6.62392969	6.72855072	6.58051539	6.87507597	7.3540305	7.5983272	7.373859	7.1849303	7.3828684	7.28501081	7.27147866	7.47573997	7.649194921	7.207393842	7.118584614
Bra026322	5.854758354	5.997934672	6.04285335	5.95841257	6.02215715	5.97489864	5.88951948	5.92948045	6.0250022	5.8161003	6.0285924	5.9396663	5.96489744	6.08568293	6.090835888	6.256822558	6.171105614	6.144074088
Bra026351	7.462800959	7.544519299	7.41864633	7.09844012	7.26889693	6.99314282	8.06118988	7.81894753	7.7660091	7.3333639	7.5222585	7.0491805	8.29304323	8.12451661	8.000663589	7.384163457	9.163132571	8.813276588
Bra026352	6.007270851	6.04523387	6.05360182	6.15971738	6.05122955	6.03777042	6.1742042	6.08378799	6.1804279	6.1032147	6.0864929	6.1393223	6.28503788	6.09634924	6.291020928	5.87952227	6.105025147	6.012674993
Bra026368	5.964586074	5.950892073	6.04042245	6.07483234	6.07001663	5.84575421	6.25814634	6.13359008	6.0418984	6.1591714	5.900917	6.0959728	6.16469833	6.13877744	5.983826357	5.986747352	6.032268806	5.803152064
Bra026369	8.665337448	8.440378	8.69481946	8.62027861	8.62188498	8.36105906	8.18467096	8.19393895	7.905517	8.0665256	7.4288462	7.6360128	7.85064232	8.0191711	7.591741728	7.834489393	6.608258297	6.855866043
Bra026385	7.769235751	7.747124481	7.48398156	8.2073518	7.55884028	8.21859786	7.97999708	7.61173396	8.0876707	7.9720109	8.7120612	8.3256815	7.50690501	7.51225613	6.834271276	7.686610228	6.422771831	7.554700147
Bra026525	10.53397333	10.57986319	9.63542172	9.55944316	8.67522345	8.82312444	10.1463892	10.4974503	10.1089	9.4937039	9.4653991	8.8744885	11.2097708	11.1045657	9.477517388	10.03484763	10.44777615	11.89894655
Bra026526	5.892382353	5.975039739	5.95030796	6.05169114	5.90739108	6.02308844	5.84786488	5.97444205	5.9380849	6.044384	6.0211228	5.8765941	5.87663556	5.83819693	5.882150946	5.980220045	5.730754665	5.891624321
Bra027149	6.718092777	6.654497584	6.49742782	6.74178933	6.32357001	6.76469474	6.60085323	6.39438011	6.4783661	6.4586581	6.4636645	6.3592667	6.66200622	6.63993422	6.265662075	6.225298362	6.301052662	7.528181383
Bra027187	7.745856141	7.003061528	7.25567315	7.1871453	8.14515658	7.67791145	6.54869315	7.26696687	6.7284181	6.2710491	6.9687238	7.1200655	8.11814269	7.96361322	8.258630575	8.777971416	7.407980498	7.15883412
Bra027203	7.918888775	8.058320685	6.72228811	7.14826303	6.80656348	6.86653007	8.57654173	7.36684572	7.2934885	7.0623678	7.2138311	7.2033511	7.72033458	8.10823677	7.870436885	7.509770344	7.910226333	7.502664233
Bra027245	6.484160912	6.589324437	6.87060427	6.70468184	7.03488038	6.84797036	6.69095048	6.72955721	6.8596138	7.2012394	6.825828	7.2454347	6.5401505	6.51353435	7.028470917	7.05125544	6.815110138	6.580098884
Bra027250	11.68918745	11.42612839	11.5734294	11.458528	11.2688408	10.9203642	12.688914	11.9686555	12.111324	12.574391	11.734002	11.967376	10.5317232	11.3299844	11.02242388	10.77709072	10.22537916	10.62078884
Bra027260	7.330847863	7.30972394	7.76447868	7.80503141	8.07170571	8.08315602	7.12576477	7.2076897	7.2818554	7.5990204	7.6779252	7.9119514	7.55023297	7.5012684	8.05917594	7.950838436	7.292811221	7.123336922
Bra027261	6.050836603	6.07719237	6.03906977	6.11999818	6.15678634	5.9696435	6.10668028	6.20734283	6.1354966	6.0907358	6.2086071	6.1959153	6.14996089	6.08870946	5.995050935	6.156866388	6.112597927	5.921038666
Bra027263	9.90823947	9.966558172	9.66298896	10.3868426	9.66869887	10.2713461	10.2038447	10.1175498	10.637583	10.20447	10.543715	10.135414	10.0228518	10.3490634	9.038793863	10.60205469	8.493238364	11.85770169
Bra027264	6.018859417	5.968025265	5.92687824	5.87479619	6.13442896	6.04483637	5.96250441	6.11397693	5.8241595	5.8545741	5.8245942	5.9557193	6.00180006	5.98583902	6.095112809	6.067084638	6.044394798	6.011071853
Bra027286	7.508217366	7.644338376	8.77791103	9.85257312	10.9929652	11.6956623	7.69323719	7.27714554	10.263568	9.3951994	11.227579	10.84639	7.63109286	7.4628162	8.892606164	9.284164548	10.17004925	9.889197733
Bra027289	5.954561974	6.004646064	5.98739175	6.01649728	5.99547165	5.92506209	5.83685849	5.97557798	5.8918387	5.9985511	5.9365531	5.9743635	5.9865984	5.85633811	5.936922545	5.976168299	6.016062877	6.007830639
Bra027290	9.615860406	9.517526044	9.85504203	9.2235666	9.75379476	9.2915152	9.45669887	9.54832686	9.4638426	9.1722154	9.3153459	9.0810043	8.22556353	7.86795975	8.233300433	7.991963636	8.677240695	8.690683239
Bra027302	6.485429695	6.560840665	6.17983475	6.18506461	6.31213212	6.29748788	6.79588587	6.91270811	6.8234509	6.6951206	6.6671321	6.39367	6.73149948	6.65105405	6.383161588	6.391854724	6.505328704	6.336500895
Bra027408	5.993124634	6.076119063	6.13118522	6.15890753	5.99743863	6.06597818	6.0005605	5.98463296	6.0381255	6.1018795	6.0271627	6.1500747	6.10056667	6.09141247	6.177950511	6.074050869	5.974103744	6.133260336
Bra027520	7.323975646	7.251918366	7.10186919	6.95086441	7.04680812	6.96960433	7.14635517	7.3488836	7.2523026	7.1256176	6.9365078	6.7750687	7.10812783	7.03051127	7.147484534	7.01425508	6.788108648	6.676351928
Bra027817	7.244797124	7.040103068	7.54208265	7.12632724	6.9998282	6.93723652	7.04426107	7.02005875	6.8163347	6.7177538	6.9279104	6.7161522	7.63682127	7.49994261	7.151094304	6.897263598	7.892866851	7.750068246
Bra027818	6.953444481	7.080717012	6.57874824	6.95913075	6.28874607	6.77941084	6.68011449	6.80584	6.5485214	6.4792024	6.6119011	6.4516908	6.97648567	7.03013443	6.516038281	6.55697569	6.879717256	6.792234479
Bra027819	6.067232732	6.003427526	6.03037233	6.0361005	6.02082605	5.95590601	5.92342102	6.08986248	5.9269679	5.9226763	5.9970778	5.9643806	5.9242197	5.94483965	6.100764182	5.910088261	5.967114829	5.954939412
Bra027890	6.275704116	6.051067193	6.32104909	6.10868017	6.28635636	6.18615642	6.31212966	6.29616784	6.3290082	6.2503208	6.2309796	6.2127919	6.27039998	6.29112799	6.375957772	6.251466688	6.300050121	6.267796716
Bra027891	5.935756578	6.03531738	6.13653203	5.94748607	5.90742312	6.07837504	5.93151397	5.92562253	5.9471631	5.9270229	5.9447001	6.0097038	5.81569898	6.085134	5.872703635	5.923472024	6.012232058	5.903707039
Bra027896	6.148201553	6.133752258	6.10333613	6.10003357	6.18440721	6.21685373	6.10807077	6.03887737	6.239879	6.4985694	6.7037701	6.4087479	6.09985849	6.14116085	6.272567203	6.198691268	6.173974994	6.303229673
Bra028379	6.749410129	7.109423722	8.1882636	8.38890411	6.79765188	8.45364626	6.46050907	6.81777491	6.5149993	6.8444632	7.4007511	8.5673825	6.55422191	6.64690973	6.8212771	6.849540195	6.588295037	6.469147571
Bra028380	7.952691562	7.901995875	6.85999104	7.498211	7.11785913	7.6133989	7.53647687	7.32239	7.3808146	7.2339927	7.2305897	7.3947558	7.54866298	7.9577954	8.076050478	8.019240852	7.916512015	7.743738595
Bra028407	6.213046902	6.345647769	6.39406834	6.29815073	6.25885954	6.20450712	6.01563407	5.92364985	5.9649627	5.8866795	5.9895058	5.9957087	5.86026317	6.08234204	5.910140331	5.842090674	5.962598704	6.041187458
Bra028411	11.85251987	11.8176042	11.2752993	10.992872	10.6058236	10.3330852	12.3727049	11.8249037	10.837083	11.038542	10.319604	10.124358	11.5378582	11.9402137	11.44852565	11.10934435	10.16211649	9.589214861
Bra028412	5.93599136	5.877316267	5.93477762	5.87285341	5.84082942	5.83941288	5.9120308	5.88292503	5.9320597	5.982595	5.8684335	5.9657056	5.86394035	5.84338199	5.896261428	5.864694356	5.985802078	5.754560559
Bra028460	6.307306805	6.20304948	6.27898781	6.12865173	6.15570354	6.21722188	6.38721853	6.29272355	6.3893669	6.3116505	6.1915371	6.2685031	6.41857358	6.28717457	6.221044505	6.305446099	6.098447185	6.2475877
Bra028518	6.507446489	6.631031096	6.66130839	6.37442543	6.77734055	6.54080311	6.89782058	6.79465267	6.6344007	7.0351389	6.668678	7.0567539	6.65043079	6.57515405	6.895353264	7.132403625	6.802229785	6.504418498
Bra028525	6.589754629	6.452642016	6.89687846	6.6054953	6.88794798	6.84492564	6.74351708	7.14439847	7.3032296	7.2766227	7.2590109	7.4451995	7.12531366	6.91312212	7.257224523	7.106314208	7.726475441	7.626384577
Bra028540	7.77331004	8.027775337	7.34995912	7.32541293	7.19283515	7.20546227	6.55232023	7.10676801	6.8469409	6.7402875	6.6386693	7.0734322	8.22648136	8.32091627	7.705644223	7.837758811	7.164306649	7.212539264
Bra029597	7.473397643	7.186899879	7.54517271	7.26098253	7.18408661	7.04743313	6.41106179	6.53292358	6.2239702	6.3456192	6.4186391	6.2398059	7.2396658	7.019725	7.727930341	7.104449196	8.913514778	8.041315358
Bra029713	6.482626901	6.396666132	6.29535817	6.13284107	6.33870918	6.19964671	6.76794111	6.9343947	6.6059765	6.454486	6.4115999	6.4470974	6.76003325	6.73812209	6.498806272	6.400971966	6.745047729	6.210837214
Bra029739	6.189540392	6.061915137	6.12640239	6.15983327	6.11356241	6.16426065	6.27563946	6.10359754	6.2337056	6.311104	6.2430797	6.4677576	6.14444018	6.1775171	6.231386647	6.325843268	6.40700147	6.472977459
Bra029803	8.10370798	8.313089572	8.02962174	8.06667223	8.88129378	8.715909	8.02520378	8.05600747	8.3486685	8.7952353	8.2736798	8.478907	7.82881627	8.12725463	8.468891787	8.728318139	7.987067678	8.3637908
Bra029846	6.198166383	6.266870421	6.18408526	6.21406512	6.35803466	6.26820477	6.2252111	6.00385232	6.18773	6.1340954	6.1729423	6.1636406	6.01550846	6.11562081	6.258997463	6.147568234	6.262586775	6.03383761
Bra029847	12.81133495	12.79622217	11.2467502	12.4714105	12.1224412	12.8802529	13.7939773	12.7708928	13.109699	13.0221	13.165113	12.656587	11.6477516	12.4068842	10.80392941	12.24795161	9.257274313	11.86470208
Bra029866	7.631388911	7.423105263	7.25251312	7.31595326	7.05702398	7.21702429	7.86074517	7.66423566	7.5342226	7.0811739	7.4334444	7.0969671	8.1969269	8.1046628	7.972984878	7.440439661	9.099089743	8.879509776
Bra029868	5.817212374	5.973006911	6.07264521	6.10444711	6.02044388	5.95182825	5.99326121	5.99013677	6.0669061	6.008588	6.0104769	6.0667845	6.02449951	6.10488557	5.817272142	5.944348356	5.994853003	6.061211228

Bra030055	6.506872328	6.540630123	6.66272261	6.48349153	6.58250302	6.59428887	6.68345711	6.77069063	6.5226769	6.7443314	6.4687979	6.6860796	6.67458711	6.68930245	6.838938006	6.834716553	7.171938572	7.005942431
Bra030114	7.676480858	7.741395672	7.39216385	7.35934316	7.40973168	7.1979577	8.65128383	8.75185344	8.4980818	8.6020491	8.1921772	8.3311703	8.38124068	8.64381146	8.594688766	8.604437338	7.846206987	7.53775234
Bra030118	5.915614024	5.965502405	5.97605197	5.94862441	6.01725928	5.99982762	6.0284891	5.98655353	5.9045614	5.991617	6.0391272	6.1229446	5.9476771	5.91811569	5.965322507	5.944084011	5.859587219	5.883249157
Bra030119	6.115498619	6.028835945	6.12896909	6.04832043	6.16641394	6.06618945	6.17095886	6.12880739	6.1645338	5.9786162	6.1566512	6.2500097	6.06637532	6.02933836	6.085943668	6.224573937	6.294378847	6.189906331
Bra030124	7.618806935	7.375686052	7.43392863	7.45606522	8.11870375	7.38087846	7.2203688	7.20072871	7.5273775	7.9282934	7.5911707	7.5181859	6.98626306	7.02146066	6.911373446	7.203981072	6.96258515	7.012093664
Bra030125	6.423271645	6.26062467	6.42126875	6.38521187	7.04448466	6.35219624	6.3462382	6.29719668	6.5588933	6.7880692	6.6851949	6.6848087	6.44603028	6.56219664	6.705329411	6.588092399	6.970530296	7.17774897
Bra030306	9.211612468	9.38616845	8.23396281	8.7472553	8.00559274	8.03950344	9.19878384	8.67407031	8.2559766	8.7991964	8.054976	8.2249585	8.02545769	8.42054068	8.087222957	8.118341415	6.858192182	6.941947198
Bra030307	6.629859453	6.55773693	6.52847811	6.59644895	6.88261002	6.89527373	6.73653386	6.648088	6.5153964	6.5870497	6.5695033	6.5425632	6.78313767	7.09870647	7.030119491	7.604245149	6.543664886	7.209372679
Bra030841	6.832645439	6.637840929	6.99312038	6.69001732	7.36731066	6.84999507	6.7920634	6.92383066	7.0866552	6.9453272	7.2190736	6.9687023	7.04290863	7.06867888	7.307135457	7.177371684	7.837292816	7.866262648
Bra030842	6.544162518	6.646578794	6.38600403	6.49110765	6.23115345	6.33119443	6.32182573	6.04145817	6.3067607	6.1972926	6.190777	6.2509548	6.36175053	6.3390548	6.067219954	6.121164153	6.124562618	6.098334225
Bra030949	6.155027008	6.193526458	6.32415573	6.24236263	6.27667425	6.24005306	6.10058849	5.92011788	6.0803791	6.0967582	6.0937194	6.0136941	5.9411599	6.06983579	5.979640505	5.976906143	6.085662681	6.071512125
Bra030966	6.614563703	6.632230202	7.13196961	6.83418086	7.20131682	6.72185609	6.81685833	6.73396756	6.9319626	6.6574695	6.6845691	6.5053929	6.3887341	6.28400416	6.396088558	6.385734011	6.533698512	6.654685403
Bra030971	6.073915179	6.214462994	5.95046233	6.31047388	6.09301979	6.21372979	6.12936468	6.33690433	6.2935969	6.1673729	6.1489426	6.2295784	6.10869523	6.13603456	6.199035	6.026949489	6.225919499	6.292893344
Bra030972	5.968134482	6.020699229	5.90813377	5.97949269	6.09288102	5.97842728	5.99710544	6.03790008	6.077527	5.9775784	6.0480871	5.9304559	5.90216408	6.02984105	5.926060378	6.102429397	6.233881951	6.094040272
Bra030975	6.417901302	6.186190083	6.15713995	6.12090341	6.10981681	6.16367837	6.22123189	6.50648939	6.2990167	6.1928235	6.2973243	6.3255706	6.5218893	6.40316476	6.476329824	6.357835264	6.770038598	6.256283483
Bra030976	6.117295889	6.084215672	6.20002183	6.24981082	6.03431184	6.16541894	6.15220228	6.05904444	6.0398281	5.9784244	5.9995212	6.03671	6.13163112	6.09900628	6.128113312	6.170862858	6.178138238	6.320681935
Bra031319	7.017004034	6.883012915	7.19692556	6.78302551	6.92993278	6.89053766	6.43974225	6.5980413	6.3722159	6.4057014	6.3432945	6.4067942	7.2817371	7.37912296	7.065751347	6.73126843	7.90199388	8.034634735
Bra031320	6.162097118	6.150575828	6.09049851	6.02099816	6.03681646	5.99887284	6.0282316	6.00432507	5.7969621	6.0803764	5.9830996	5.9598097	6.06316658	6.03848719	6.16397503	6.020655467	6.055577479	6.036339735
Bra031392	6.032536664	5.955965433	6.08772384	5.94673941	6.30828091	6.61553438	6.0777469	6.14684035	6.1999479	6.0535562	7.3452419	7.4923817	6.03112225	5.99336805	6.033779853	5.9061623	6.066508453	6.303552478
Bra031393	10.37721329	10.47543838	11.7539832	11.3649458	11.2811724	12.7669302	11.2208579	11.482743	8.3666631	6.988123	8.4588401	9.4174356	11.4929316	12.100804	10.94110829	11.88261494	10.062889	9.114525482
Bra031394	6.063715222	6.067057766	6.0607115	6.00273488	6.09860707	5.91936775	6.05945641	6.12478099	5.9752296	5.990546	6.0550238	5.9568936	6.10101836	6.0038383	6.107063802	6.048041326	6.030959172	6.086653065
Bra031395	9.020786512	9.073876194	8.84444593	9.13382056	9.11187427	9.27077098	9.30354448	9.49766724	9.2922156	9.7726216	9.7379969	10.051604	9.06462969	9.43880726	9.883842165	9.921571502	8.575496004	8.374163105
Bra032175	8.594086562	8.424303613	8.41845876	8.23935318	8.93098846	8.5411817	8.7945493	8.67230285	8.6180387	8.647594	8.6085872	8.3885843	8.53453387	8.24351719	8.422168028	8.099275321	8.283567501	8.352785597
Bra032701	6.405911611	6.440670685	6.50508731	6.1658055	6.45521668	6.29646835	6.36229658	6.61715149	6.6271757	6.4846579	6.2598539	6.646924	6.54856524	6.57615026	6.43636637	6.755947039	6.385095451	6.46820506
Bra032719	6.913255613	7.111227137	7.05269929	6.76543742	6.89152076	6.8871773	7.06884256	7.40864597	7.1099885	7.5464867	7.0738464	7.2323475	7.53255756	7.53394313	7.11620987	7.125180417	6.849630057	7.124838771
Bra032720	6.622295755	6.752120568	6.40563871	6.68896407	6.18396656	6.43880498	6.23952143	6.19440955	6.2487246	6.1774036	6.1656664	6.2268619	6.24424427	6.3712216	6.116154589	6.223007973	6.065614053	6.48830884
Bra032888	5.934615867	6.044691115	5.91762672	5.86858069	6.0085482	5.89041579	6.12960881	6.09450466	6.0254701	5.9985727	6.0540352	6.062939	5.99158264	6.04717635	6.094238303	6.014188087	6.096731826	6.086187722
Bra033877	5.987739528	5.979695746	5.92742297	5.91139131	5.94196419	6.03964096	6.12202958	5.97057813	5.9860344	6.0720621	5.9352061	5.9902924	5.98737427	5.9232362	5.989509215	5.987202998	6.025501334	6.031890989
Bra034681	6.325849584	6.419144205	6.27602702	6.32970229	6.21424971	6.22302619	6.0628909	6.06289408	6.0127682	6.0700229	6.1576282	6.2475862	6.03387448	6.18919231	6.160553423	6.163075993	6.173050742	5.97860584
Bra034682	6.098051684	6.163242425	6.28129346	6.04530809	6.28805718	6.12589555	6.19704113	6.23703208	6.1358012	6.35124	6.0565996	5.971716	6.5343055	6.13899228	6.006362309	6.181373204	6.076169115	6.031813092
Bra034691	7.6500797	8.08869233	6.74478258	7.75066432	7.2877591	8.70391571	7.28711784	6.95949099	7.5932786	6.7445306	8.8759556	8.0284889	7.50850907	7.71976417	7.65077192	7.619163527	7.431449239	7.368736267
Bra034734	7.146309301	6.986857814	7.20356226	7.2894743	8.01826297	7.72497198	7.30068136	7.41761204	7.5118308	8.083076	7.9410216	8.2139167	6.8324786	7.29303115	7.511195235	8.029915763	6.871974411	7.35650933
Bra034735	7.429482236	7.841965872	7.56189121	8.10918235	9.49426935	8.62997236	6.97575049	7.16270312	7.760907	8.1983106	7.9699081	7.8015128	7.20385982	7.29644406	7.003028723	7.226305625	6.845500117	7.16202077
Bra034743	6.186973983	6.083757938	6.12304682	6.1905233	6.07631678	6.0906281	6.30414654	6.25443804	6.2329544	6.124019	6.2389901	6.1674073	6.27646393	6.20655135	6.068526229	6.099190049	6.350641135	6.305726259
Bra034773	6.0800518	6.145245318	6.10714515	6.05786864	5.93600606	5.98899829	5.98114517	6.08194587	6.0587346	6.0920651	6.0712497	6.0031982	6.15498815	6.12303369	6.01372982	6.005216246	6.193270611	6.087143562
Bra034774	7.270761527	7.174668105	7.73265599	7.86295552	7.64723188	8.03912779	7.07808793	7.06225452	6.9853835	7.0227105	7.0085517	7.2964728	7.04454404	7.17677531	7.366387047	7.415971249	6.678927091	7.169320903
Bra034822	8.747180297	8.387089304	8.65192999	9.52997289	9.96938476	10.0135718	9.74639403	8.86999062	10.200956	10.269222	10.47267	9.7830923	8.2398692	8.42219159	8.953331838	8.838491982	8.846389983	9.065567722
Bra034823	6.414551426	6.430410228	6.51919725	6.5148188	6.67353806	6.68713155	6.77442915	6.7421736	6.6288231	6.8517702	6.918011	7.0958305	6.44652972	6.77974345	6.589106883	6.740554419	6.863378091	6.67913132
Bra034827	6.699552671	6.704467302	6.75722433	6.68168257	6.43924181	6.57901138	6.64234463	6.50167878	6.6923198	6.8450842	6.5038341	6.6002068	6.61861113	6.69496371	6.511495669	6.48594467	6.742576769	6.744100468
Bra034844	6.200514181	6.305899721	6.3463164	6.26031114	6.37468502	6.30220753	6.24454747	6.15586324	6.2429263	6.3162518	6.3641728	6.4673847	6.34839451	6.36473573	6.397229782	6.39296163	6.380175199	6.287668249
Bra034845	6.203393235	6.190924879	6.51259121	6.78671904	6.50935253	7.34340633	6.1391376	6.0850263	6.2528328	6.4486923	7.0983434	6.8910951	6.11728676	6.15247847	6.413579211	6.462052994	6.503029087	6.535481462
Bra034872	6.099268381	6.187832993	6.07928798	6.33444701	6.06365133	6.2881241	6.00276667	6.09631636	6.1389789	6.1274742	6.1462715	6.05459	6.18124221	6.15291538	6.259418208	6.038956199	6.028812855	6.058863948
Bra034875	5.96105309	6.073079262	6.24635854	6.03246951	6.09965362	6.20719771	6.1365799	6.15718379	6.1661178	6.2362217	6.0884194	6.392487	6.06836083	6.12324145	6.099554432	6.154747796	6.237868974	6.193303254
Bra034876	6.049001723	6.10317297	5.85714698	6.11775632	5.99214301	6.12962299	6.14721763	6.15309237	5.9769747	6.2066642	6.2974131	6.280751	6.31182815	5.99971685	6.456479685	5.917670002	6.150785629	6.065478906
Bra034878	6.559916592	6.402281552	6.25802652	6.0801273	6.36933816	6.30377004	6.43073801	6.94661082	6.6744784	6.7207878	6.534221	6.7072727	6.74354532	6.721971	6.703404859	6.893886959	6.392590603	6.516291951
Bra034880	7.1033614	6.966788157	6.57284668	6.52121426	6.7701534	6.79162744	7.02159823	6.70662708	6.7449391	6.8461544	6.6224653	6.6321467	7.10852514	7.23567425	7.133193473	7.529055363	7.255758858	7.155393066
Bra034897	10.61288779	10.41458589	9.20251052	9.21403462	9.17807535	9.19181553	10.0848887	9.78523871	9.2315367	9.3013338	8.8516619	8.9428228	9.93782958	10.2885929	7.842207264	9.09783139	6.289501484	7.821169781
Bra034898	11.41717597	11.42591999	10.2614611	10.8401634	9.70199216	10.3055705	11.1986145	10.972779	10.595202	10.592958	9.9739581	9.9542489	10.6289799	11.0648832	8.600308819	9.857747307	6.859185671	9.290553273
Bra034920	5.90568483	6.071710216	6.14540225	6.10601449	5.99760494	6.04813087	6.13332235	5.92796097	5.9494451	6.1394024	5.9938994	5.9928435	6.08306719	5.96896222	6.053912159	6.013028943	5.97325938	5.942486301
Bra035521	6.551167466	6.462365848	6.61641361	6.50283617	6.89520013	6.65717083	6.25578812	6.31418057	6.3390567	6.3572397	6.3239313	6.6131624	6.79523855	6.74098801	6.704251367	6.696626429	6.485253198	6.412622412
Bra035522	6.147967226	6.262824103	6.6080484	6.36569736	6.28813908	6.73467592	6.27853148	6.19135611	6.2119149	6.2335806	6.3162797	6.1809521	6.22372282	6.4568101	6.38672416	6.4489072	6.268008509	6.212639626
Bra035664	6.537565159	6.588495716	6.15474965	6.2616346	6.4139251	6.25537716	6.958329	7.11225763	7.000064	7.1751424	6.721956	6.8145135	6.58157121	6.78937446	6.617515943	6.761799736	6.401041932	6.323902255
Bra035665	7.115093714	7.085510046	6.63341377	6.96467088	6.80619702	6.89379332	7.11724253	6.4638624	6.8434623	6.8152508	6.6352795	6.662453	6.79815419	6.75027113	6.673290473	6.751878492	6.436617909	6.349348828
Bra036280	6.294472087	6.403134924	6.38373684	6.30842536	6.33123935	6.31907533	6.30366633	6.02953976	6.3793888	6.0480222	6.2338524	6.2322827	6.36246101	6.49612186	6.25539761	6.368632442	6.4070431	6.371882094
Bra036639	8.497763842	8.829388805	7.91272222	7.72512759	8.67950914	8.05516179	8.84711867	8.31172331	8.3208434	8.2386801	8.1580282	7.9736741	8.41175986	8.30039926	8.031004619	8.049340059	7.724270089	7.511446291
Bra036640	6.75959038	6.627045162	6.44688214	6.39774686	6.45939453	6.40384324	6.31872978	6.16674747	6.2768654	6.5533004	6.3389009	5.9631048	6.38177767	6.30822468	6.545660882	6.280028947	6.448892393	6.34758449
Bra036734	5.859147812	5.999296829	6.01595238	6.01391884	6.05225387	5.97060033	6.08856559	6.08668236	6.1902235	6.0275731	5.9630722	6.0910162	6.04576626	5.88376675	6.105295966	5.823264058	6.000355475	6.002941358
Bra036871	6.046131708	6.159450576	6.01969804	6.0329142	6.15926919	6.14738955	6.52102052	6.49176971	6.7310369	6.5422064	6.9227636	6.6894046	6.12584186	6.09215342	6.780992951	6.199607712	6.501859166	6.254507538

Bra037302	6.479046029	6.48211902	6.34011901	6.4376797	6.35301458	6.68751363	6.52766443	6.44444472	6.6159093	6.5913225	6.5742791	6.7764472	6.46792928	6.34799212	6.632161885	6.495073307	6.961181669	6.669289177
Bra037382	7.146224569	7.128764231	7.4376361	7.68679206	7.13388055	7.81258597	7.03771801	7.14673424	7.1129685	7.2609982	7.3424233	7.7271736	7.20955477	7.04609195	7.223175112	7.201601283	7.352036365	7.963976386
Bra037425	6.347238839	6.31679302	6.20488975	6.17446498	6.22527373	6.30417975	6.33056352	6.04992635	6.2380092	6.0605536	6.3203868	6.1747302	6.38942996	6.16907361	6.284043434	6.151112064	6.431549278	6.302450138
Bra037426	5.79791955	5.963431712	5.90596484	5.97938136	5.99448594	5.88447287	6.12639545	5.99225555	6.1318765	5.9429413	6.1099538	5.9245188	6.05135284	6.08115451	5.973075462	5.965305684	5.964184238	5.954949212
Bra037495	9.170343159	9.013322958	9.80340074	9.87252082	10.1309628	9.78757685	9.86288171	9.60548778	9.8101317	10.281613	10.304284	10.472217	8.74810872	8.88530913	9.438362103	9.527686136	8.906291322	9.375871105
Bra037514	6.73219489	6.658826946	6.64186037	6.68967933	6.59946937	6.50393522	6.54970979	6.40511534	6.3550248	6.5932356	6.3707341	6.3995128	6.82226807	6.8265883	6.442204099	6.63616842	6.591569452	6.398215082
Bra037667	6.791188085	6.740318955	8.04190246	8.58911386	7.90583217	9.21191724	7.11089611	7.01108402	7.9612436	7.9086015	8.8678135	8.6823161	6.88335598	6.89931304	9.057395151	8.866942386	8.206081938	8.009012278
Bra037668	6.264139461	6.207098123	6.11002127	6.13380556	6.22111664	6.10162555	6.20562357	6.43653142	6.0331038	6.326336	6.1753146	6.3163301	6.47506845	6.23538271	6.396201499	6.028773195	6.285738243	6.350046968
Bra038073	5.899917443	5.967851696	5.95994091	6.10229663	5.89616252	6.11596313	5.94654441	6.23099741	5.7413526	6.0003563	6.0557546	5.9290795	5.8580137	6.01047739	6.050422554	6.034491767	5.948480176	6.093122879
Bra038074	5.958602388	5.985474552	6.17428251	6.08097579	6.1305629	6.01560132	6.003604	6.19314671	6.0659725	5.9923008	5.9597618	6.1000563	6.16343499	6.0777645	6.047850702	5.925633586	6.218614633	6.030748204
Bra038075	6.130213311	6.020201933	5.97543656	5.92361303	5.96078129	6.04954128	5.99754033	6.06187891	5.8754562	6.0743058	6.0079425	5.9332071	5.92813927	5.91062565	5.997596856	6.020169104	6.047429887	5.988891675
Bra038076	5.935507438	6.017697151	5.93587397	6.17124614	6.02587482	6.09506007	6.08092543	6.09658421	6.1189279	6.0558263	6.0917374	5.9844999	6.11039337	6.11231184	5.9523001	5.937765358	6.03538157	5.907235273
Bra038077	5.952385958	5.909870008	5.8761907	5.97463405	5.99220235	5.84279405	5.93990436	6.02254298	5.8653742	6.0120342	5.9345758	5.9241346	5.75158024	5.9601418	5.847553174	6.062064006	5.908036536	5.974404725
Bra038094	6.02864461	5.962122418	6.13349167	6.08830915	5.98610988	5.96737885	6.04049913	5.97598132	6.2344545	6.3561247	7.1089513	6.7300489	5.92193381	6.09943471	6.431144122	7.165591968	6.10311711	6.057726212
Bra038280	6.189878124	6.187165217	6.26103371	6.11713152	6.33110391	6.19469994	6.46495875	6.34742083	6.3269718	6.3437289	6.3764391	6.5019566	6.33911489	6.26234943	6.286401914	6.319454663	6.366620311	6.335124259
Bra038426	6.051995544	6.184836224	6.28103538	6.21745775	6.16351687	6.10020037	6.37081401	6.16346094	6.251953	6.2074584	6.0172205	6.2220894	6.16917973	5.98925201	6.099602583	6.056721433	6.097452631	6.063123174
Bra038427	6.250599256	6.509490556	6.50641043	6.57604663	6.64288199	6.49464456	6.46614915	6.43862264	6.3517761	6.4020439	6.4478185	6.5097574	6.26430714	6.41188306	6.535373639	6.666320367	6.393291454	6.222639621
Bra038632	6.056107476	5.970861626	5.99406194	5.87487923	6.03657622	6.03348404	5.85746944	5.8877672	6.0278444	5.9797381	6.0597403	6.032676	5.93159293	6.09098016	5.945218346	5.886640841	5.93566642	5.956222748
Bra038643	8.409545754	8.307668524	8.89794799	8.79833826	9.57621192	9.33907453	8.46830053	8.6555516	9.1365105	8.6865465	8.8972849	8.5756974	6.77535528	6.90769794	6.703613979	6.499020673	7.116730541	7.246029793
Bra038644	6.189270137	6.140033339	6.42525398	6.29633496	6.25268426	6.14955534	6.15034261	6.05653851	6.2741593	6.3932242	6.2455075	6.1005317	6.97386044	6.49918183	6.650108828	6.314830568	6.732020067	6.658307025
Bra038848	5.944444833	5.913685499	5.95234285	5.99408761	6.00525091	5.88183283	5.96153175	5.99225761	5.9117793	5.9672581	5.8385724	6.1147754	6.03581327	5.85154206	5.923352362	6.005763263	6.002351764	6.073491227
Bra038849	6.556501263	6.516115936	6.60413954	6.5347661	6.74045975	6.77914988	8.16319023	7.75192033	7.7643042	7.679774	7.4381081	7.3668415	7.8565267	8.0125765	7.906124064	7.664516481	7.959389982	7.72836345
Bra038936	6.846162439	6.757438401	7.15965784	7.31708197	7.2628668	7.0632759	6.69498984	6.90932717	6.76482	7.088685	6.9003528	7.0506248	6.72011748	6.71952559	6.822529451	6.783613064	6.98705791	6.715153983
Bra038937	6.621619938	6.630278534	6.73680683	6.84084153	7.16256647	7.1876509	7.06942365	7.09451493	7.3240931	6.9884622	7.1730451	7.1320503	6.80865325	6.82080006	6.94953325	6.945071988	7.319542707	7.120110003
Bra038944	7.089368167	7.161145125	6.76372357	7.24724244	6.81290175	7.00160252	6.85099175	6.72593712	6.925478	6.7574979	6.7502846	6.7517686	7.02888788	6.98962683	6.823591386	6.88078159	6.994209052	7.256750978
Bra038953	5.955706541	6.008596657	5.97798604	5.95466362	6.00269965	5.95667855	5.87418698	5.99033457	5.8750334	5.9266896	5.8897696	5.9119242	5.88249424	5.9809367	5.946442046	6.036018658	6.056770446	6.040875007
Bra038954	6.251540957	6.211918736	6.15409541	6.14830267	6.18664282	6.17429232	5.94797332	6.07939071	5.9334635	6.0385544	6.0376481	6.1338528	6.0702517	6.01078966	6.057765147	6.041033984	5.971230012	6.017967615
Bra038959	8.323635231	8.326329268	8.48044623	8.09691379	8.1316369	8.00068487	8.07933463	8.1000588	7.973567	7.7334981	7.80894	7.6130193	8.20659741	7.9486386	7.641664577	7.464571519	8.039008637	7.917998158
Bra038960	6.374006866	6.241140192	6.39170261	6.40326762	6.54129626	6.38064224	6.50862607	6.66098762	6.737809	6.9069745	6.6512867	6.9359578	6.26442925	6.29443389	6.415630831	6.591347688	6.173903361	6.250972153
Bra039030	6.540878998	6.681941108	6.72267163	6.67053983	6.79697941	6.89656103	6.03592323	6.27872479	6.22575	6.2323024	6.3248292	6.3886356	6.7358635	7.01991531	6.763297251	7.204136838	6.499912749	7.05097591
Bra039420	6.599834857	6.440740029	6.09265267	6.23614411	6.14121503	6.14447618	6.1565228	6.07075942	6.208743	5.9864182	6.1758445	5.9750292	6.04821791	6.33767632	6.079483061	6.21971474	5.91108706	6.160671341
Bra039421	6.09426208	5.926132636	6.05566657	5.96631839	5.92892839	6.04266571	6.18275428	6.07468172	6.1029092	5.9780704	6.1250049	5.9973778	6.10466345	5.99504214	6.228625853	6.148082627	6.370194752	6.151065294
Bra039431	6.201512536	6.215542792	6.37651534	6.29676838	6.53053446	6.50038618	6.55563994	6.40893657	6.5818426	6.6598456	6.6157965	6.6271189	6.10037399	6.44766454	6.363514258	6.508811229	6.564932026	6.458457496
Bra039440	8.485769423	8.803919726	6.96735149	7.74702262	7.15428792	7.6422246	8.26840868	7.32007514	7.7721542	7.3132757	7.5523144	7.5474644	8.07925815	8.23702865	7.861579754	7.735713405	6.917346309	6.897911778
Bra039444	8.861130588	9.571066579	8.50931262	9.28522965	8.44028757	8.7899546	9.86288874	9.45762982	9.3331095	9.4369123	8.9796228	8.843474	9.15635255	9.63581508	7.857876267	9.125121249	6.512679772	8.07340914
Bra039445	6.847385118	6.954862177	6.81825189	6.58240307	6.90716394	6.70427436	7.0216538	7.08214629	6.9877296	6.9908778	7.0191373	6.8071171	6.7604998	6.75307276	6.79025057	6.865448442	6.53702742	6.59506979
Bra039446	7.551472512	7.713733387	7.09893768	7.11612063	7.03226138	6.76149966	7.48477801	7.89286984	7.3907572	7.110123	6.9058841	7.1825111	7.99561285	8.03940136	7.226838066	7.496947255	6.711633894	6.86231899
Bra039447	7.151468939	7.274335149	7.5900051	7.83205275	8.06332847	7.84249291	7.14462659	7.01624907	7.1434688	7.4646183	7.6359398	7.6719865	6.77431763	7.21322135	7.479322835	7.805059596	6.930300368	7.417553452
Bra039461	6.145125817	6.080386787	6.2400264	6.08997123	6.3011237	6.06179691	6.19025086	6.08838673	6.2953518	6.3048764	6.329542	6.2552856	6.46401312	6.20034637	6.576950305	6.429219553	7.16290833	6.75956264
Bra039462	6.346768792	6.288457015	6.47957355	6.39272602	6.56025422	6.61307182	6.74744074	6.6138682	6.8285176	6.9333115	6.7912156	6.9291429	6.54033799	6.52809184	6.356926463	6.816366311	6.21048219	6.416871553
Bra039477	6.02276327	5.910595402	6.0287624	6.00783079	6.01233613	5.92775083	6.06888364	6.04328794	5.8957628	6.044995	6.0160898	5.950384	6.00304422	5.96455358	6.013487126	5.911727115	6.143088025	5.949537287
Bra039548	9.05878979	9.004951417	9.47261806	9.03898316	9.46010774	8.86823895	8.50880905	8.80501413	9.0923913	9.1423291	9.1793858	8.9223897	9.7696205	9.4740154	9.719479451	9.689015461	9.879730147	9.821107211
Bra039627	6.72773972	6.783906484	7.36845255	7.30533111	7.66401486	7.4010234	6.99440391	6.8043925	6.7971885	7.335103	7.4986581	7.8272548	6.59356891	6.71854927	7.441162405	7.309280645	7.062253772	6.942651258
Bra040338	5.927888077	5.748272512	5.94168077	5.79144227	5.88596942	5.90810062	6.10191317	5.92298352	6.2167661	5.930478	6.1317561	5.9020143	6.00106657	5.93334821	5.816208104	5.835714889	6.046603999	5.98650323
Bra040339	6.246625554	6.342104921	6.33180472	6.11343819	6.20425203	6.21893036	6.3178126	6.15300565	6.2961238	6.2236813	6.1364196	6.2739173	6.13942124	6.36176134	6.233875178	6.187064029	6.562429227	6.449504925
Bra040340	6.154966726	6.198613066	6.20432086	6.13809927	6.12179257	6.18670453	5.91896281	6.09691703	5.8926087	5.9859639	5.8329285	5.9985379	6.01205606	5.85810751	6.032426851	5.947380206	6.142988463	6.08030766
